# The mass spectrometric intact transition epitope mapping method supports protein engineering of foldon trimer variants

**DOI:** 10.1038/s41598-025-28101-7

**Published:** 2025-11-22

**Authors:** Cornelia Koy, Timo Zimmer, Kwabena F. M. Opuni, Armin Geyer, Michael O. Glocker

**Affiliations:** 1 Proteome Center Rostock, Medical Faculty and Natural Science Faculty, University of Rostock, Schillingallee 69, 18057 Rostock, Germany; 2 Department of Chemistry, Philipps-University Marburg, Hans-Meerwein-Straße 4, 35032 Marburg, Germany; 3 Department of Pharmaceutical Chemistry, School of Pharmacy, College of Health Science, University of Ghana, P.O. Box LG43, Legon, Ghana

**Keywords:** Protein engineering, Non-covalent complexes, T4Ff foldon variants, nanoESI-MS, ITEM mass spectrometry, Binding strength analysis, Biochemistry, Chemistry

## Abstract

The mini-protein T4 Fibritin foldon (T4Ff) allows targeted protein–protein interaction studies by protein engineering which alters primary structure to influence higher-order structures. T4Ff variants revealed how non-natural N-terminally located amino acid residues with large π-electron rings affected trimer stability. Mass spectrometric ITEM-FIVE analyses determined apparent kinetic and quasi-thermodynamic properties of trimer dissociation reactions in the gas phase. Results presented here show that aromatic π-stacking stabilizes T4Ff variant homo-trimers which otherwise were less stable than the original T4Ff because of inner-chain amino acid exchanges. The T4Ff activation enthalpy of 63.9 kJ/mol dropped to 51.8 kJ/mol for its Gly10→D-Ala and Asp17→D-Phe-containing variant. This drop was counterbalanced by additionally placing amino acid residues with aromatic side chains at the N-termini. The diphenyl amino acid side chain-carrying variant is slightly more stable than the original T4Ff (activation enthalpy: 66.8 kJ/mol). ITEM-FIVE proved capable of determining non-covalent force differences with amino acid residue resolution.

**Supplementary Information:**

The online version contains supplementary material available at 10.1038/s41598-025-28101-7.

## Introduction

The primary goals of protein engineering are (i) to improve protein stability^1^, (ii) modify binding properties^2^, and (iii) alter catalytic reactivity, or (iv) selectivity^3^. The list includes gaining a deeper understanding of protein oligomerization^4^ by focusing on small building block units^5^ which have become of particular interest for the study of protein self-assembly processes^6^. The T4 Fibritin foldon (T4Ff) has been subject of targeted manipulations in protein engineering studies since long^7,8^. The T4Ff miniprotein encompasses 27 amino acids with a strong tendency to trimerize in a simple fold comprising a β-hairpin that is preceded by a type-II polyproline helix^9^. T4Ff’s folding kinetics has been considered as evolutionary optimized for fast and efficient trimerization of fibritin^10^, one of the T4 phage’s structural protein. T4Ff has often been used in biophysical studies, in cooperative protein folding and oligomerization studies^11^, and in analyzing the structural features of similar fibrous proteins fused to the foldon domain^12^. The extraordinary trimerization initiation potential of T4Ff has been made use of for engineering protein needles^13^, bio-nanomachines for punctuating host cells^14^ and larger self-assembling viroid units^15^. Moreover, T4Ff has been applied to engineer metal-chelating bipyridine units with a stereo-selective assembly due to the three-stranded T4Ff peptide arrangement with defined orientation^16^.

Solid-phase peptide synthesis allows the variation of the amino acid assembly in a peptide at will. But chemical synthesis comes with the problem of aspartimide formation at Asp-Gly dipeptide sequences^17^. The lability of Asp-Gly (DG) di-amino acid units, which had already been described for natural peptides and proteins^18^, affects first of all the proteins’ primary structures. In T4Ff Asp^9^-Gly^10^ terminates the PPII helix where Gly adopts positive torsion angles, which was mimicked by replacing Gly^10^ with D-Ala^10^. The second Asp^17^-Gly^18^ motif of T4Ff was exchanged by D-Phe^17^-Gly^18^ or by the Hot^17^ = Tap^18^ bicyclic dipeptide residue which consists of hydroxythreonine (Hot) and thiaproline (Tap).^19^ The chosen amino acid replacements facilitated chemical synthesis of T4Ff variants without drastically affecting T4Ff’s distinct backbone and side-chain features which are required for forming stable secondary structures as well as for self-assembly processes of T4Ff in high yields, *i.e.* for adopting the correct quarternary structure^19^. High resolution crystal structures of the chemically synthesized T4Ff variant trimers (2ww6 and 2ww7) show that they resemble the native fold of T4Ff trimers^20^. Other protein engineering efforts also result in simultaneously affected different protein structure levels. Thermal stability of T4Ff homo-trimers was enhanced by engineering a covalent interchain clamp between the three monomer peptide strands via triazido-functionalized trimesic acid scaffold attachment^21^. Similar trimer binding force-strengthening effects were supposed to be obtained by addition of large amino acid residues with aromatic side chains, thereby enabling π-stacking^22^ or gearing effects^23,24^ instead of covalently connecting the T4Ff monomer chains.

With the advent of electrospray MS (ESI-MS)^25^, mass spectrometric methods have entered the stage of higher order protein structure analysis, such as the determination of stoichiometries of protein–ligand and of protein–protein complexes^26,27^. Ion mobility measurement capabilities have been added to mass spectrometers and have been successfully applied for determining collisional cross sections (CCS) of bio-macromolecules, such as proteins^28^. Of note, CCS determinations had been applied to monitor effects of amino acid modifications on molecular sizes^29^. Novel developments and applications of mass spectrometric methods enable to quantify non-covalent protein complex binding forces and strengthen the understanding of protein–protein interactions in general^30^. And when combined with protein engineering they provide precise information about decisive property changes that had been achieved with the desired synthetic product^31,32^.

The mass spectrometric ITEM method uses stepwise CID–derived dissociation to generate survival yield curves which are then analyzed using Boltzmann fitting and its subsequent kinetic and thermodynamic interpretation. There are previous works where such treatment of CID measurements is accepted to calculate the thermodynamic functions^33^. ITEM (Intact Transition Epitope Mapping) enables to determine apparent kinetic and quasi-thermodynamic values, such as free energies and enthalpies of complex dissociation reactions in the gas phase. ITEM-ONE identifies peptide ligands of proteins by determining the mass of the complex-released peptide upon dissociation of protein complex ions in the gas phase^34^. Determining kinetic and thermodynamic properties, that is, quantitative traits of protein complexes, is possible using the ITEM-TWO method^35,36^. ITEM-THREE identifies unknown antigenic determinants on an antigen surface by mass spectrometric sequencing of the complex-released peptides, *i.e.*, the epitopes^37^. And the ITEM-FOUR procedure targets at fine mapping of binding regions, that is, the determination of recognition motifs within a bound peptide with amino acid residue resolution^32,38,39^. Recently, ITEM-FIVE (Intact Transition Epitope Mapping - Force Interferences by Variable Extensions) was introduced which is capable of determining the roles of chemically added linkers and functional groups which mimicked intrinsically disordered regions in a protein complex with respect to its non-covalent stability^31^.

Two questions were interrogated here using our recently developed mass spectrometric ITEM method: (1) can chemically engineered T4Ff variants form non-covalent trimers? and if so, (2) are the modification-carrying T4Ff derivatives forming weaker or even stronger bound trimers than the original T4Ff? Equipped with the ITEM armamentarium we engaged in the mass spectrometric analysis of trimer stabilities of T4Ff (foldon 0) as well as of the trimer stabilities of chemically engineered T4fF variants (foldons 1–6). Foldons 0–6 were synthesized by solid phase peptide synthesis. Foldons 1–6 contained C-terminal carboxamide instead of carboxylic acid and D-amino-acids at positions 10 (G10a) and 17 (D17f). Foldons 2–6 were engineered to possess in addition artificial N-termini. Foldons 3–6 have Gly1 removed. Foldons 5 and 6 contain N-terminally located amino acid residues with increased aromatic side chains.

## Results

### Molecular characterization of the T4Ff homo-trimer and its variants

Chemically synthesized T4Ff and its variants (Table 1; Fig. S1) showed that they predominantly contained mixtures of monomers and homo-trimers. Interestingly, the expected multiply protonated trimer ion signal series is extremely narrow and consists of only one charge state with measurable abundance using the chosen solvent conditions. Foldon homo-trimers were represented in the nanoESI mass spectra by their quintuply protonated ion signals and the monomers with doubly and triply protonated ion signals with different ratios of ion signal intensities.

**Table 1:** Amino acid sequences and molecular features of T4Ff (foldon 0) and its variants (foldons 1–6).

Foldon no. / symbol	Amino acid sequence ^a)^	Atom no. ^b)^	*m/z* (exp.)^b, c)^	*m/z* (calc.)^b, d)^
0 /	H-Gly^1^Tyr^2^IPEAPRDGly^10^QAYVRKAsp^17^GEWVLLSTFL-OH	1299	1848.94	1848.75
1 /	H-Gly^1^Tyr^2^IPEAPRDala^10^QAYVRKphe^17^GEWVLLSTFL-NH_2_	1332	1875.99	1875.80
2 /	H-Gly^1^tyr^2^IPEAPRDala^10^QAYVRKphe^17^GEWVLLSTFL-NH_2_	1332	1875.95	1875.80
3 /	H-Tyr^2^IPEAPRDala^10^QAYVRKphe^17^GEWVLLSTFL-NH_2_	1311	1841.69	1841.58
4 /	H-tyr^2^IPEAPRDala^10^QAYVRKphe^17^GEWVLLSTFL-NH_2_	1311	1841.78	1841.58
5 /	H-Nal^2^IPEAPRDala^10^QAYVRKphe^17^GEWVLLSTFL-NH_2_	1326	1862.01	1862.00
6 /	H-Dip^2^IPEAPRDala^10^QAYVRKphe^17^GEWVLLSTFL-NH_2_	1338	1877.92	1877.61

^a)^ single letter code, except for positions which were exchanged by non-natural amino acid residues. D-amino acids are shown in small print. Superscripts indicate amino acid positions ^b)^ trimer, ^c)^ 5 + ion signal, ^d)^ calculated from sequence.

**Fig. 1:**
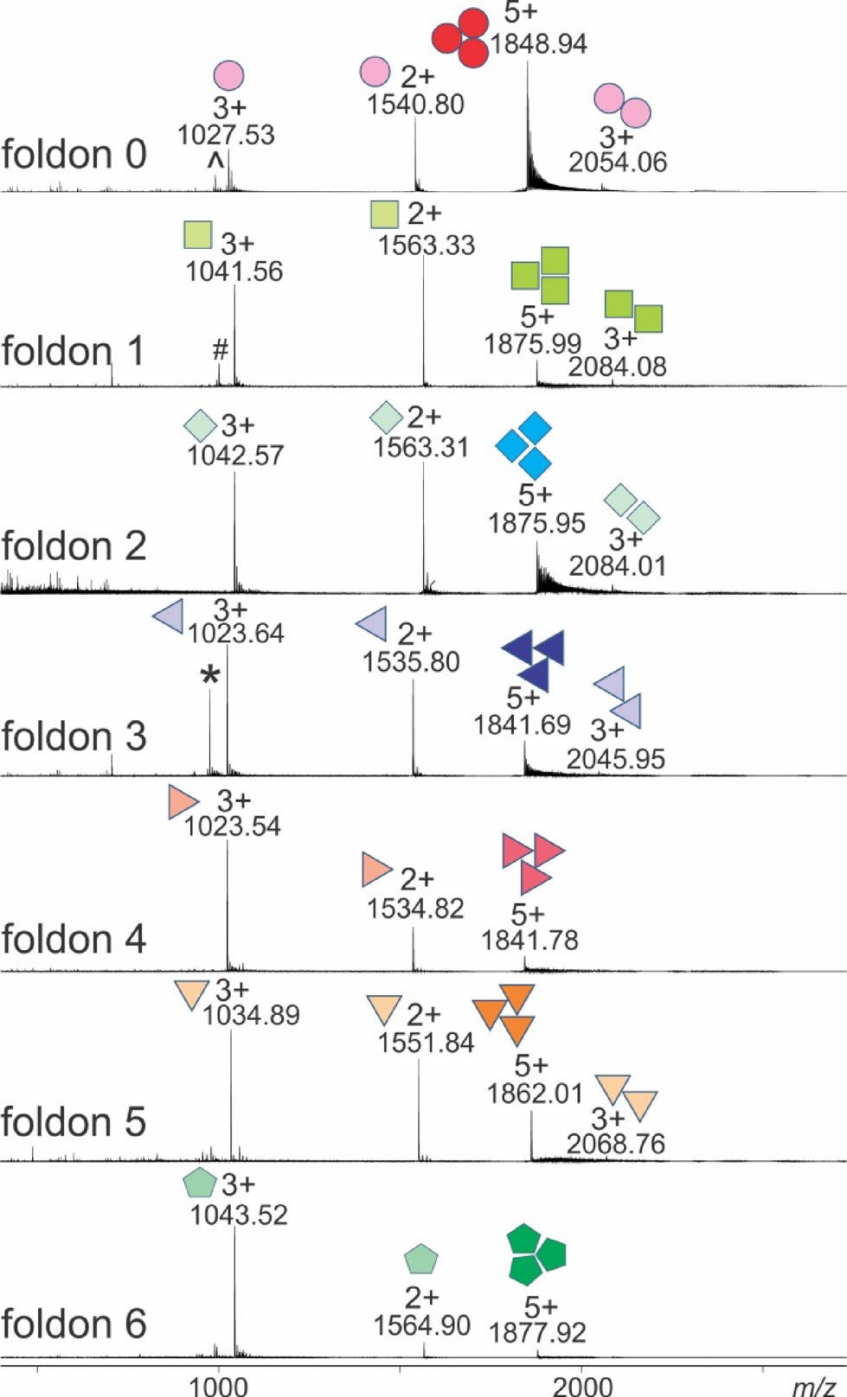
NanoESI mass spectra of trimeric T4Ff (foldon 0) and chemically synthesized foldon variants 1–6. Multiply charged ion signals of trimers, dimers, and monomers are assigned with *m/z* values and charge states. Symbols indicate oligomeric states. Ion signals of truncated monomers are labeled with symbols (see text for explanations). Solvent: 50 mM ammonium acetate, pH 5.5.

In case of foldon 0 (T4Ff), the 5 + trimer ion was present as the most intense ion signal in the spectrum. Traces of dimers were also found in the mass spectra by weak triply protonated ion signals (Fig. 1).

Foldons 0, 1, and 3 contained in addition to the respective monomers and trimers fair amounts of truncated monomeric peptides. According to their *m/z* values the truncated foldon 0 monomer lacks the L27 residue which was proven by mass spectrometric fragmentation of the triply protonated monomer ion (Fig. S2). Truncated foldon 1 lacks the K16 residue (Fig. S3), and in truncated foldon 3 the f17 residue is missing (Fig. S4). However, trimers with truncations were not detected. Only, trimers of foldons 0 and 2 contained sodium adducts in larger quantities as was judged from the series of satellite ion signals on the high mass sides of the quintuply protonated trimer ion signals (Fig. 1).

The trimer ion signals proved by their experimentally determined *m/z* values that all complexes consisted exclusively of homo-trimers which matched with the expected amino acid compositions (Table 1 and Fig. S1). After inspection by nanoESI-MS, all foldon-containing solutions were considered suitable for subsequent ITEM-FIVE analyses since the requested homo-trimer ion signals have sufficient strength.

### ITEM-FIVE analysis of dissociation reactions of the T4Ff homo-trimer and its variants

**Fig. 2:**
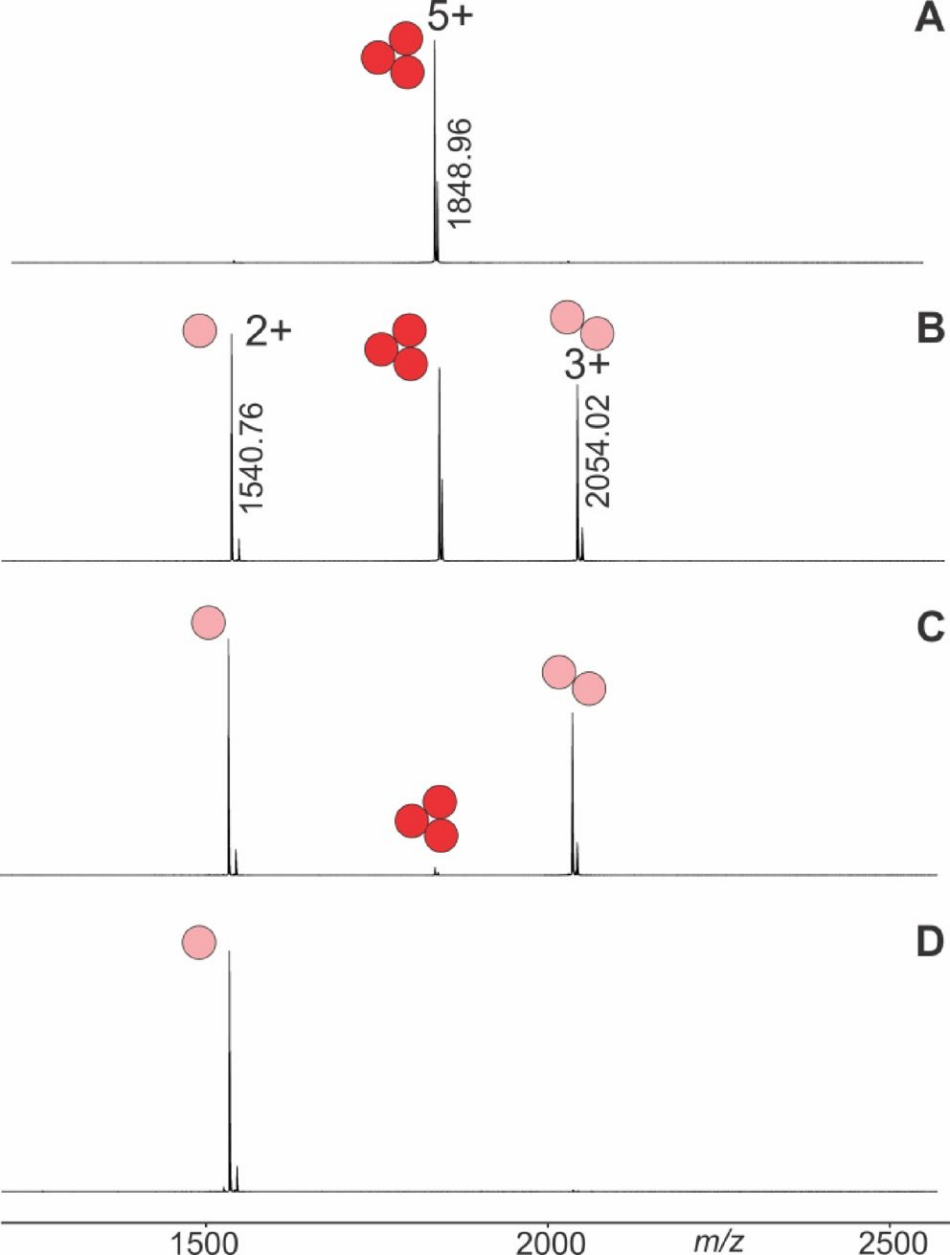
Nano-ESI mass spectra of T4Ff (foldon 0) with increasing collision cell voltage differences (∆CV). (**A**) 0 V, (**B**) 15 V, (**C**) 20 V, (**D**) 40 V. Charge states and *m/z* values of ion signals are given. The quadrupole was set to isolate the 5 + trimer ion. Solvent: 50 mM ammonium acetate, pH 5.5.

ITEM-FIVE investigations started with electrospraying foldon-containing solutions and by setting the first mass filter (quadrupole) to selecting only foldon trimer ions for transmission into the instrument’s collision cell. In case of T4Ff (foldon 0), the only ion signal which represented the homo-trimers was the quintuply protonated ion signal at *m/z* 1848.96 (Fig. 2A).

**Fig. 3:**
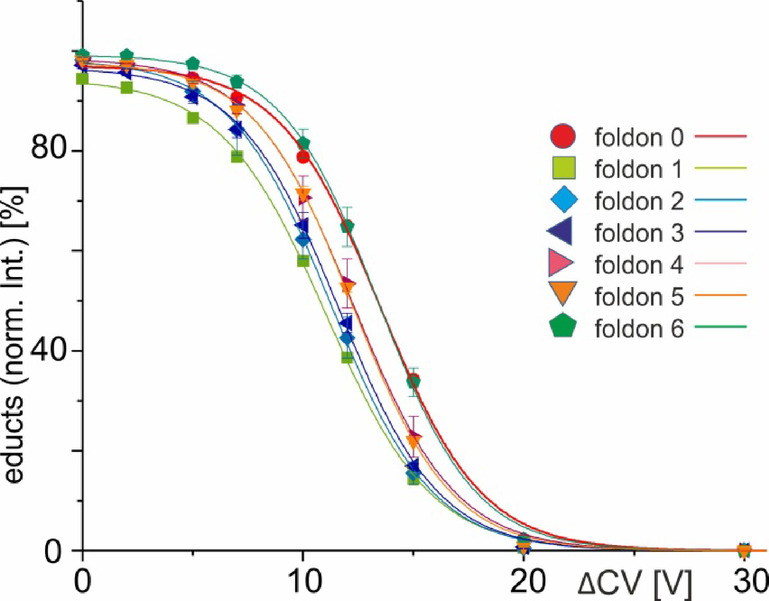
Courses of normalized foldon 0–6 trimer ion intensities plotted as functions of collision cell voltage differences (ΔCV). Each data point is the mean of two independent measurements and standard deviations are shown by vertical bars. Curves were fitted using Boltzmann functions (cf. Table S2). For symbol assignments see also Table 1; Figs. 1 and S1.

After mass filtering, this was the only remaining ion signal in the mass spectrum because the energy in the collision cell was too low to initiate complex dissociation. T4Ff homo-trimer dissociation was observed by increasing the collision cell voltage difference (∆CV) in a step-wise manner and at 15 V (Fig. 2B) the mass spectra showed ion signals of surviving trimers (educts) flanked by ion signals of dimers and monomers (products). At 20 V the trimer ion signals were barely visible (Fig. 2C), leaving mass spectra with dominating ion signals of dimers and monomers. Finally, at 40 V, the only ion signals which remained visible in the mass spectra were those of the monomers (Fig. 2D). Independent measurement series of complex dissociations were recorded twice for each foldon variant (Tables S1–S7). ITEM-FIVE experiments were, one after the other, performed with all other foldon variants providing very comparable mass spectra to each other within the respective ∆CV settings (Figs. S5–S10). Plotting the courses of normalized educt ion intensities as functions of ∆CV provided sigmoidal shaped curves with Boltzmann characteristics for all foldons (Fig. 3).

Interestingly, the ∆CV_50_ values dropped from 13.5 V for T4Ff (foldon 0) to 11.0 V for foldon 1 and gradually increased again with each foldon variant with the next higher reference number providing somewhat higher ∆CV_50_ values until up to 13.4 V for foldon 6 (Table S8). Approximating the tangent lines at the ∆CV_50_ values of all the Boltzmann curves (R^2^ = 0.999 for all foldons; cf. Table S8) and upon transforming ∆CV values to reaction temperatures (applying formula (5) see Supplement) enabled drawing of Arrhenius plots (Fig. S11, applying formulas (1) and (5)) and Ellingham diagrams (Fig. S12, formulas (3), (4) and (5)). The $\:{\text{k}}_{\text{m}\text{g}}^{\#}$ values were calculated from formulas (2), (5) and (6). The kinetic values ($\:{\text{l}\text{n}\:\text{k}}_{\text{m}\text{g}}^{\#}$) followed in all Arrhenius plots linear courses when plotted against $\:\frac{1}{{\text{T}}_{\text{c}\text{o}\text{l}\text{l}}}$ and T4Ff (foldon 0) and its variants (foldons 1–6) lined up in nearly parallel lines (according to formula (1)). Likewise, the pseudo thermodynamic activation free energy values ($\:{{\Delta\:}\text{G}}_{\text{m}\text{g}}^{\#}$), followed in all Ellingham diagrams courses with almost parallel lining up of linear courses of all foldons when plotted against $\:{\text{T}}_{\text{c}\text{o}\text{l}\text{l}}$ (according to formula (4)). From these experimentally determined data points on the respective lines were extracted the apparent kinetic and pseudo thermodynamic properties of the foldon 0–6 trimer dissociation reactions by linear extrapolation to ambient temperature (Figs. S11 and S12), representing conditions of complex dissociation without external energy contributions, such as vibrational excitation of molecules and ion acceleration (Table 2).

**Table 2:** Apparent kinetic and pseudo thermodynamic properties of foldon 0–6 trimer dissociation reactions in the gas phase.

Foldon / symbol	Amino acid sequence ^a)^	k^#^_m0g_^b, c)^ [Ø]	K_D_^#^_m0g_^b, c)^ [Ø]	ΔG^#^_m0g_^c)^ [kJ/mol]	ΔH^#^_m0g_^c)^ [kJ/mol]	T_amb_ΔS^#^_m0g_^c)^ [kJ/mol]
0 /	H-**Gly**^**1**^**Tyr**^**2**^IPEAPRD**Gly**^**10**^QAYVRK**Asp**^**17**^GEWVLLSTFL-OH	3.61 × 10^8^	5.81 × 10^–5^	23.9	63.9	40.0
1 /	H-**Gly**^**1**^**Tyr**^**2**^IPEAPRD**ala**^**10**^QAYVRK**phe**^**17**^GEWVLLSTFL-NH_2_	6.61 × 10^9^	1.06 × 10^–3^	16.5	51.8	35.3
2 /	H-**Gly**^**1**^**tyr**^**2**^IPEAPRD**ala**^**10**^QAYVRK**phe**^**17**^GEWVLLSTFL-NH_2_	3.60 × 10^9^	5.80 × 10^–4^	18.5	56.3	37.8
3 /	H-**Tyr**^**2**^IPEAPRD**ala**^**10**^QAYVRK**phe**^**17**^GEWVLLSTFL-NH_2_	2.64 × 10^9^	4.25 × 10^–4^	19.1	56.7	37.6
4 /	H-**tyr**^**2**^IPEAPRD**ala**^**10**^QAYVRK**phe**^**17**^GEWVLLSTFL-NH_2_	1.17 × 10^9^	1.89 × 10^–4^	21.2	60.0	38.8
5 /	H-**Nal**^**2**^IPEAPRD**ala**^**10**^QAYVRK**phe**^**17**^GEWVLLSTFL-NH_2_	1.22 × 10^9^	1.97 × 10^–4^	21.1	61.1	40.0
6 /	H-**Dip**^**2**^IPEAPRD**ala**^**10**^QAYVRK**phe**^**17**^GEWVLLSTFL-NH_2_	3.47 × 10^8^	5.60 × 10^–5^	24.6	66.8	42.2

^a)^ single letter code, except for positions which were exchanged by non-natural amino acid residues. D-amino acids are shown in small print. Superscripts indicate amino acid positions. ^b)^ dimensionless number, ^(c)^ m0g: **m**ean charge state with **0** external energy contributions in the **g**as phase.

Positive $\:{{\Delta\:}\text{G}}_{\text{m}0\text{g}}^{\#}$ values indicate that complex dissociation reactions in the gas phase are not spontaneous (endergonic) and positive $\:{{\Delta\:}\text{H}}_{\text{m}0\text{g}}^{\#}$ values mean that complex dissociations are endothermic. The positive $\:{\text{T}{\Delta\:}\text{S}}_{\text{m}0\text{g}}^{\#}$ values show that entropies increased during dissociations of the homo-trimer complexes. The drop of $\:{{\Delta\:}\text{H}}_{\text{m}0\text{g}}^{\#}$ from 63.9 kJ/mol for T4Ff (foldon 0) to 51.8 kJ/mol for foldon 1 is explained as being the result of exchanging the native amino acid residues G at position 10 and D at position 17 to D-amino acid residues a and f, respectively. This drop in enthalpy of activation is counterbalanced by the introduction of large artificial amino acid residues with extended side chains at the N-termini of the foldon variants. Due to the expanded aromatic ring systems they are capable of exerting intercatenane π-stacking interactions. The $\:{{\Delta\:}\text{H}}_{\text{m}0\text{g}}^{\#}$ value of foldon 6, 66.8 kJ/mol, is slightly above the value from foldon 0 (Table 2).

### Structure analysis and molecular dynamics of the T4Ff homo-trimer and its variant foldon 1

Molecular modelling and comparison of the 3D structures of foldon homo-trimers was done with the aim to estimate stability differences and was based on the atom coordinates of foldons 0 (1rfo) and 1 (2ww6) which had been determined by X-ray structure analysis. A HADDOCK score of below − 100 a.u. indicates reliable 3D structure models of the “docked” homo-trimers, which was expected since the input data were directly taken from the X-ray structure data files. Interestingly, docking modeling revealed a significant difference in the van der Waals energies which clearly favored foldon 0 over foldon 1 whereas with the other energy terms, such as electrostatic energy the opposite resulted (Table 3).

**Table 3:** Structure model characterizing metrics of the T4Ff foldon 0 and foldon 1 trimers.

Foldon / symbol	pdb file	HADDOCK score ^a)^ (a.u.)	RMSD ^b)^ (Å)	vdW en. ^c)^ (kJ / mol)	el. en. ^d)^ (kJ / mol)	des. en. ^e)^ (kJ / mol)	RV en. ^f)^ (kJ / mol)
0 /	1rfo	-130.0 ± 18.7	13.4 ± 0.2	-368.6 ± 33.9	-444.3 ± 166.9	-115.9 ± 25.5	293.3 ± 44.9
1 /	2ww6	-155.3 ± 3.2	9.0 ± 2.7	-317.6 ± 40.6	-919.6 ± 222.2	-161.9 ± 14.2	116.7 ± 41.0

^a)^ a.u.: arbitrary units. ^b)^ root mean square deviation (RMSD) from the overall lowest-energy structure. ^c)^ van der Waals energy. ^d)^ electrostatic energy. ^e)^ desolvation energy. ^f)^ restraints violation energy.

The calculated van der Waals energy differences correlate with lesser intercatenane hydrophobic contacts of the homo-trimers’ carbon atoms (Table 4 and Tables S9 and S10) in foldon 1 (18%; 69 of a total of 380) as compared to those of foldon 0 (28%; 162 of a total of 579).

**Table 4:** Comparison of non-covalent force interactions within Foldon 0 and Foldon 1 homo-trimers.

Force type ^a)^	Foldon 0 /			Foldon 1 /		
	Intra (%) ^b)^	Inter (%) ^b)^	Total (%)	Intra (%) ^b)^	Inter (%) ^b)^	Total (%)
van der Waals	417 (72)	162 (28)	579 (100)	311 (82)	69 (18)	380 (100)
Hydrogen bonds	232 (90)	25 (10)	257 (100)	194 (92)	17 (8)	211 (100)
Salt bridges	10 (56)	8 (44)	18 (100)	9 (43)	12 (57)	21 (100)

^a)^ numbers of interactions between carbon atoms only. Percent values are shown in parentheses. ^b)^ intra: intracatenane; inter: intercatenane.

Since hydrophobic interactions exponentially decay with atom–atom distances, the intercatenane Cα atom distances were investigated (Tables S11 and S12) and showed that the sum of the distances between all the Cα atoms in the homo-trimers was smaller for foldon 0 (364.12 Å) as compared to the sum of Cα atom distances for foldon 1 (379.62 Å), indicating that the foldon 1 homo-trimer is slightly less compact than the foldon 0 homo-trimer. The difference in sizes of the homo-trimers is confirmed by the solvent accessible surface areas (SASAs) which in case of foldon 0 is 4305 **Å**^**2**^ and 4824 **Å**^**2**^ in case of foldon 1 (Table S13).

**Fig. 4:**
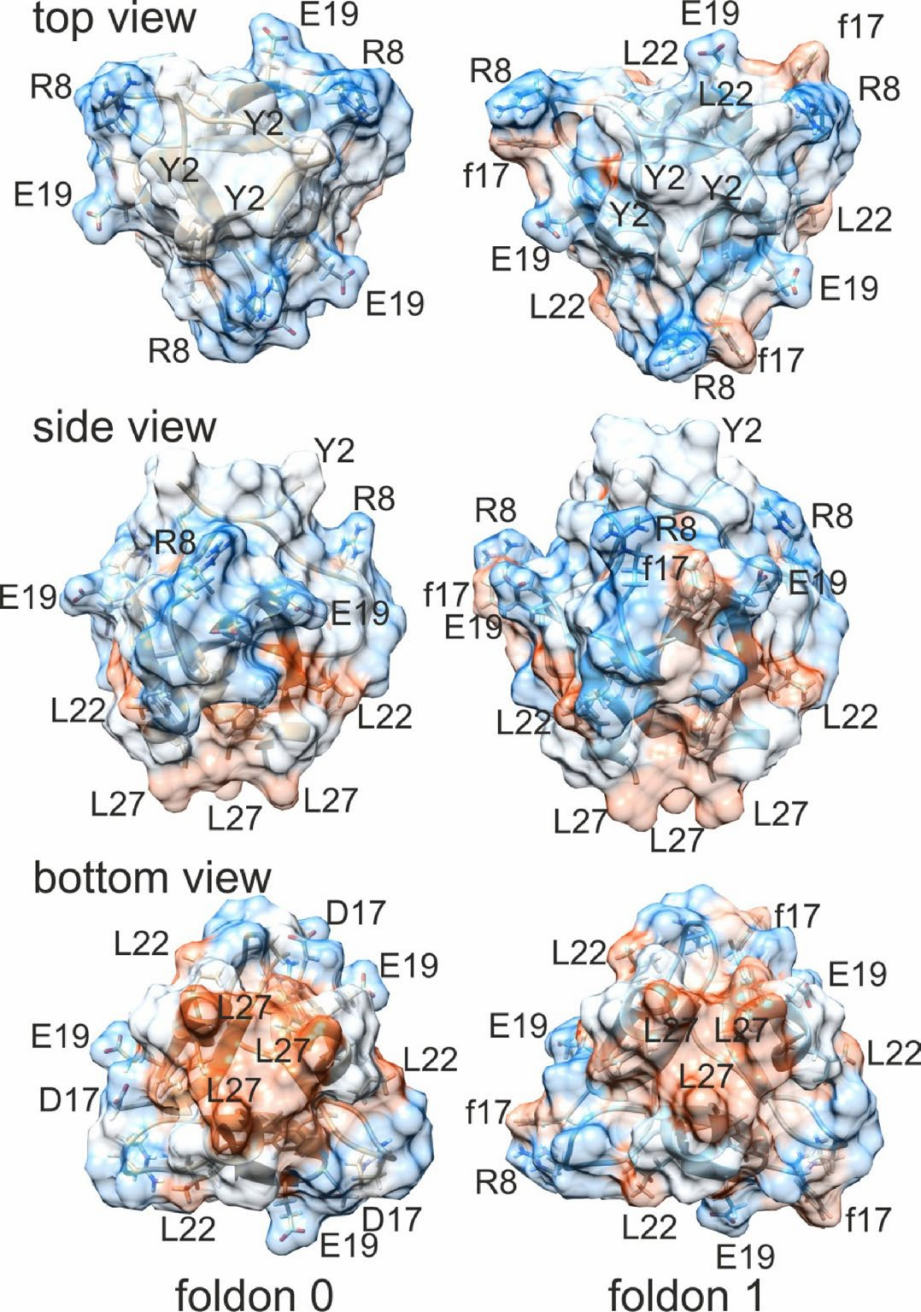
Surface views of T4Ff (foldon 0) and foldon 1 trimers’ hydrophobicity profiles. Amino acid residues are shown in single letter code. D-amino acids are written in small letters. Reddish colors indicate hydrophobic residues; blueish colors hydrophilic residues; white colors non polar residues.

Next, the distribution of hydrophobic patches on the foldon homo-trimer surfaces was visualized by displaying the van der Waals surfaces (Fig. 4) and showed that when viewed from side the supramolecular surface of foldon 0 might be split into roughly two hemispheres where the top halve, *i.e.*, near the N-termini is fairly hydrophilic and the bottom halve, *i.e.*, near the C-termini is rather hydrophobic. Both, top view and bottom view confirm the dipolar hydrophobicity distribution. In addition foldon 1 shows a disturbance of the more or less in halves split hydrophobicity pattern which is caused by f17. The clearly hydrophobic side chain of f17 stands out on the otherwise hydrophilic “upper” foldon trimer hemisphere. Moreover, the side views and top views of foldons 0 and 1 also show that the Y2 residues of each monomer are arranged differently. Whereas the Y2s’ aromatic side chains in foldon 0 are attached flat on the supramolecule’s surface, the phenyl rings of the Y2 residues in foldon 1 are oriented in a stand-up fashion above the supramolecule’s surface and assume a propeller-like triangular ring-centered but inclined π-stacking orientation (Fig. S13).

It needs to be mentioned that the numbers of intercatenane hydrogen bonds in foldon 0 are roughly the same as in foldon 1, *i.e.*, 25 (of a total of 257) in foldon 0 and 17 (of a total of 211) in foldon 1 (Table 4, S14, and S15). The numbers of intercatenane salt bridges in foldon 0 are 8 (of a total of 18) and 12 (of a total of 21) in foldon 1 (Table 4, S16, and S17), respectively, which also seems neglectable for *in-solution* stability estimations.

### CCS analysis of the T4Ff homo-trimer and its variants

The differences in intercatenane Cα atom distances and SASA between foldon 0 and foldon 1 trimers triggered the analysis of gas phase collisional cross sections (CCS) of all the foldon variants which are included in this study. Based on the travel wave drift times with nitrogen as carrier gas, the CCS for the T4Ff (foldon 1) homo-trimer,* i.e.* the 5-fold protonated ion, has been determined to be larger than that of the foldon 0 homo-trimer 5 + ion (Table 5). This increased CCS of foldon 1 stands in agreement with the larger calculated SASA and expanded intercatenane Cα atom distances. The similarity in size of Foldon 1 and 2 is explained by their difference lying just in the stereochemistry of the Y2 residues of the monomers. In foldon 1 the Y2 amino acid residues are in L configuration and in foldon 2 in D configuration.

**Table 5:** Ion mobility drift times and CCS values of foldon 0–6 homo-trimers ^a)^.

Foldon / symbol	Amino acid sequence	dt ^b, c)^ (mean) [ms]	dt std.dev. [ms]	FWHM	^TW^CCS_N2_ exp.^c)^ (mean) [Å^2^]	^TW^CCS_N2_ exp. std.dev. [Å^2^]
0 /	H-**Gly**^**1**^**Tyr**^**2**^IPEAPRD**Gly**^**10**^QAYVRK**Asp**^**17**^GEWVLLSTFL-OH	10.8	0.1	1.9	1160	3
1 /	H-**Gly**^**1**^**Tyr**^**2**^IPEAPRD**ala**^**10**^QAYVRK**phe**^**17**^GEWVLLSTFL-NH_2_	11.1	0.0	1.5	1172	0
2 /	H-**Gly**^**1**^**tyr**^**2**^IPEAPRD**ala**^**10**^QAYVRK**phe**^**17**^GEWVLLSTFL-NH_2_	11.0	0.1	1.5	1169	5
3 /	H-**Tyr**^**2**^IPEAPRD**ala**^**10**^QAYVRK**phe**^**17**^GEWVLLSTFL-NH_2_	10.9	0.1	1.2	1164	3
4 /	H-**tyr**^**2**^IPEAPRD**ala**^**10**^QAYVRK**phe**^**17**^GEWVLLSTFL-NH_2_	10.9	0.1	1.6	1164	2
5 /	H-**Nal**^**2**^IPEAPRD**ala**^**10**^QAYVRK**phe**^**17**^GEWVLLSTFL-NH_2_	11.0	0.1	0.8	1169	5
6 /	H-**Dip**^**2**^IPEAPRD**ala**^**10**^QAYVRK**phe**^**17**^GEWVLLSTFL-NH_2_	11.3	0.1	1.5	1177	3

^a)^ all foldons were dissolved in 50 mM ammonium acetate-buffer, pH 5.5, c = 0.1 mg/ml.

^b)^ centroided 5 + ion signal.

^c)^ mean of two independent measurements. See drift time profiles in Fig. S14.

Upon removal of G1 from the monomers the homo-trimer ion sizes slightly decreased. Foldons 3 and 4 differ again in the L or D configurations of the Y2 amino acid residues of each of the respective monomers. With increasing the side chain sizes of amino acid residues at each of the monomer chains’ N-termini, *i.e.* at positions 2, and with simultaneously abstaining from the N-terminal G1 amino acid residues, the homo-trimer ion sizes increase again. The 5 + ion of the foldon 5 homo-trimer which carries a naphtyl amino acid residue (NaI) in position 2 is, nevertheless, not larger than the quintuply protonated trimer ions of foldons 1 and 2 which, unlike foldon 5, both carry the G1 amino acid residue. The 5 + ion of the foldon 6 homo-trimer which lacks G1 but contains a diphenyl side chain-carrying amino acid residue (Dip) at position 2 is clearly the largest of the entire series.

The configuration of the Synapt G2S mass spectrometer allows the recording of ion mobility distribution in parallel for each ∆CV step and shows that the foldon trimer, dimer, and monomer ions do not significantly change their drift times (Tables S19 – S32), *i.e.* their CCS, upon increasing the collision cell voltage differences. CIU-IMS analyses provide complementary qualitative fingerprints of conformational changes. While experimentally determined CCS stand in agreement with individual amino acid compositions of all foldon variants, substantial drift time changes have not been detected during trimer dissociation of the investigated foldon variants.

## Conclusion

Courses of foldon trimer dissociation reactions were investigated after electrospraying the supramolecular multiply protonated ions into the gas phase. Differences in trimer stabilities in the gas phase were correlated with structural features. Compared to the T4Ff homo-trimer stability, foldon variant homo-trimers dissociate earlier upon introduction of D-amino acid residues. But complex stability increases again with larger N-terminal amino acid residues with artificial space-demanding side-chains. The most important forces which in solution keep the foldon homo-trimers together are hydrophobic interactions. The hydrophobic binding strengths are significantly larger in foldon 0 than in foldon 1. This predominance of hydrophobic interactions of foldon 0, as compared to those of foldon 1, is of much lesser importance in the gas phase. By contrast, the π-stacking related stabilizing forces in foldon variants 5 and 6 seem to remain in the gas phase and were directly quantified by ITEM-FIVE analyses. The ITEM-FIVE results show that N-terminal π-stacking counterbalances lesser foldon trimer stability which is caused by primary structure strengthening but quaternary structure destabilizing amino acid exchanges.

## Discussion

Mini-proteins, such as T4Ff have become modules for “protein design by pieces” to create more complex and longer proteins or supramolecular protein complexes because of their well-defined tertiary structures and remarkable stabilities^40^. It has been shown that T4Ff trimers can be stabilized not only by N- or C-terminal chemical cross-linking^21^ but also through enhancing non-covalent interactions which were introduced by targeted amino acid substitutions^41^ or through chemically added extensions^31^. Our results stand in line with previous studies^24,41^ and show that a reduction of trimer stability of a T4Ff variant which contained non-natural amino acid residues could be restored by introducing amino acid residues with large aromatic side chains, thereby adding aromatic ring staggering effects to increase non-covalent binding forces.

Because the drift times of ions have been found to be remarkably stable ^42^, changes of proteins’ CCS values upon increasing the voltage differences in a mass spectrometers’ collision cells have been introduced for studying gas phase unfolding processes^43–45^. CIU was shown capable of revealing unique “fingerprints of unfolding” of protein complexes where interaction sites on proteins had been probed with sets of synthetic peptides as ligands. With protein complexes of nearly the same sizes unfolding dynamics were investigated to distinguish ligand binding characteristics^46^. While CIU has been applied successfully to assess protein–ligand binding properties, its underlying concept relies on irreversible protein unfolding shortly before, during, or shortly after release of ligands from the complex. Direct characterization of protein quarternary structure characteristics has been reported upon observing protein complex dissociation by SID^47^, CID^48^, ECD/ETD^49,50^, and UVPD^51^. More or less simultaneously and based on CID-derived processes, the ITEM approach for determining binding strengths of protein complexes by mass spectrometry has emerged which has turned out to be of particular interest to protein chemists because of ease of handling and unsurpassed sample consumption, requesting only minute volumes of analytes (typically a few microliters with just micromolar protein concentrations)^34,52^. Distinctiveness of the ITEM methodology to comparable approaches ought to be mentioned. To calculate the thermodynamic functions from CID measurements, rather than using the ΔCV_50_ voltages, is driven by the need to come up with the gas-phase dissociation properties unrelated to a specific mass spectrometer setting (electrospray conditions, ion charge and initial activation). The matter is still debatable, and can only be resolved when a number of similar measurements on the same samples on a variety of different mass spectrometers will result in the same kinetic and thermodynamic values. As things stand now, it is only prudent to assume that the absolute values of those constants and functions may be instrument-dependent. It is important to acknowledge the conventionality of the absolute values in Table 2, as they have not been reproduced independently on another MS instrument. Because of this yet unresolved controversy, we use the words “quasi” and “pseudo” for description of the calculated values. Notwithstanding that, the ITEM method works when used on the same instrument to address the differences in stability of different protein complexes. Extrapolation of $\:{\text{l}\text{n}\:\text{k}}_{\text{m}\text{g}}^{\#}$ and $\:{{\Delta\:}\text{G}}_{\text{m}\text{g}}^{\#}$ to ambient (room) temperature had been done in other cases where experimental data had been difficult or impossible to obtain, *e.g.* to gain insights into long-term performance or reaction rates under normal conditions, or when transitions at lower temperatures were rare^53,54^. In addition, one has to keep in mind that the correlation of the differences in the apparent kinetic and quasi-thermodynamic constants with the expected structural changes in the foldon variants 2–6 is only postulated from general considerations and CCS measurements. In contrast, CSS correlations of foldons 0 and 1 are supported by 3D structure data. Taken together, the ITEM-FIVE method has proven useful for the investigation of subtle binding strength differences in the T4Ff variants’ trimers, i.e. the quarternary structures which were coming along with these amino acid exchanges.

Because mini-proteins serve cellular functions, *e.g.* to cope with stress^55^, and because they have become of interest as scaffolds, *e.g.* for drug candidates^56^, investigating their chemical variants’ higher order structure and stability differences has become of general interest. Examples for the exploitation of foldon trimerization capabilities were given by endeavors during SARS-CoV-2-targeting vaccine development, where cloning a foldon sequence to the vaccine target gene was considered a helpful asset in RNA-derived vaccines^57^ since it was known that trimerization of the spike protein induced a significantly higher titer of neutralizing antibodies^58^. The BNT162b1 RNA vaccine candidate which carried the foldon sequence fused to the spike protein´s receptor binding domain went into clinical trials^59–61^.

In sum, with increasingly sophisticated protein engineering goals which reach up to the quarternary structure level methods for the determination of non-covalent forces with amino acid residue resolution which are easy to handle, consume little material, and provide the necessary higher-order structure-relevant information within a decent period of time are highly desired. ITEM-FIVE mass spectrometry has shown to meet the need.

## Materials and methods

### Preparation of T4Ff, foldon variants, and stock solutions

Foldon 0 was purchased as a lyophilized TFA salt (peptides & elephants, Heringsdorf, Germany). Foldons 1–6 were synthesized according to published procedures^24^, and were provided as lyophilized TFA salts. Protein powders were stored at 4 °C until further use. Stock solutions contained foldons dissolved in 50 mM ammonium acetate, pH 5.5. PH 5.5 was chosen to stay compatible with previous NMR investigations^24^. Typical protein concentrations of the foldon 0–6 stock solutions were between 0.23 and 0.32 µg/µl. Stock solutions were stored at 4 °C until further use.

### Protein concentration determination

Protein concentrations of foldons 0–6 stock solutions were determined using the Qubit^®^ Protein Assay Kit (Life Technologies Corp., Eugene, OR, USA) as published^31^. From each foldon stock solution 2 × 3 µl were diluted with 197 µl, each, of the Qubit^®^ working solution. After incubation periods of 15 min, protein concentrations were read using the Qubit^®^ 2.0 Fluorimeter (Invitrogen AG, Carlsbad, CA, USA).

### Preparation of nanospray emmitters

NanoESI emitters for off-line measurements were prepared in-house as previously described^36^. Briefly, borosilicate glass tubes (BF 100-50-10) of 0.5 mm inner and 1 mm outer diameters were used to produce the emitters using a P-1000 Flaming/Brown Micropipette Puller System (Sutter Instrument, Novato, CA, USA). Emitters were gold-coated under argon atmosphere with the SCD005 Sputter Coater (BALTEC AG, Balzers, Liechtenstein) by setting the following parameters: current 20 mA, sputter time duration 180 s, 5 cm working distance of the emitters to the gold foil target, and argon gas pressure 0.5 mbar.

### Off-line nanoESI-MS instrument settings and data acquisition conditions

Prior to nano electrospray mass spectrometry aliquots of the appropriate foldon 0–6 working solutions were prepared by diluting foldon 0–6 stock solutions to final concentrations of 0.1 µg/µl, each, with 50 mM ammonium acetate, pH 5.5. For off-line nano ESI measurements, a volume of 3 µl of the respective foldon 0–6 working solution was loaded into one gold-coated nanoESI emitter using a microloader pipette tip (Eppendorf, Hamburg, Germany). Mass spectra were acquired on a Synapt G2-S instrument (Waters MS-Technologies, Wilmslow, UK). Instrument calibration was performed with a sodium iodide solution with a concentration of 2 mg/ml, dissolved in isopropanol/water (50:50 v/v).

All mass spectra were acquired in positive ion mode applying a mass window from *m/z* 400 to *m/z* 8000. NanoESI measurements were performed with the following instrument settings: capillary voltage: 1.0–1.2 kV, sample cone voltage: 13 V source offset: 12 V, source gas flow: 0.0 ml/min, cone gas flow: 9 l/h, helium cell gas flow: 40 ml/min, IMS gas flow: 20.0 ml/min, trap collision voltage for nanoESI-MS analysis: 0–2 V, source temperature: 40 °C; trap gas flow: 2.0 ml/min, reflectron grid 1.449 kV. Data acquisition and processing were done with the MassLynx software version 4.1 (Waters MS-Technologies, Wilmslow, UK)^31^.

### ITEM-FIVE experiments

ITEM-FIVE experiments started with isolating the quintuply charged foldon trimer ion signal as the respective precursor ion^31^. Then the collision cell voltage difference (*ΔCV*) in the trap cell was increased stepwise from: 0 V, to 2 V, 5 V, 7 V, 10 V, 12 V, 15 V, 20 V, 30 V, 40 V, and 50 V. At each *ΔCV* setting mass spectra were accumulated for 2 min and scans were combined using the MassLynx Software version 4.1 (Waters MS Technologies, Wilmslow, UK). Two measurement series were recorded. Mass spectrometry data have been deposited at the ProteomeXchange Consortium’s PRIDE repository^62^ with the dataset identifier PXD064233.

### Mass spectral data analysis and calculation of apparent kinetic and quasi-thermodynamic values

From each mass spectrum one list with mass/intensity pair data was copied into a text file and then imported into the OriginPro 2017G software (64 bit) (OriginLab Corporation, Northampton, MA, USA) where it was further processed to eliminate baseline and noise signals. Then, the processed mass list data were converted to a Microsoft Access 2010 database from which data of pre-defined target masses were extracted with a threshold of ± 0.5 Da. Target masses were selected to represent the most characteristic ion signals in the respective mass spectrum: educts were represented by the trimer’s 5 + ion signal and products by the dimer’s 3 + ion signal and the monomer’s 2 + ion signal. In this way, the mass/intensity pairs of interest were isolated from each combined mass spectrum of foldon 0–6 trimers (educts), dimers, and monomers (products) at each *ΔCV* setting. Ion signal intensities were normalized by setting the sum of all remaining target mass ion signals to 100%. Thereafter, normalized ion signals of two independent measurement series from all the components of interest were averaged and graphs of normalized intensities against *ΔCV* were plotted. Courses of educt ion intensities were fitted as Boltzmann curves with regression coefficients of 0.99 using the OriginPro 2017G software (OriginLab Corporation, Northampton, MA, USA)^31,36^. Based on the Boltzmann curves and upon transforming the *ΔCV* axis to one of temperature dependence, the corresponding Arrhenius plots and Ellingham diagrams for every foldon were generated. The principles on which the calculations of $\:{\text{k}}_{\text{m}0\text{g}}^{\#}$, $\:{\text{K}}_{\text{D}\:\text{m}0\text{g}}^{\#}$, $\:{{\Delta\:}\text{G}}_{\text{m}0\text{g}}^{\#}$, $\:{{\Delta\:}\text{H}}_{\text{m}0\text{g}}^{\#}$, and $\:{\text{T}{\Delta\:}\text{S}}_{\text{m}0\text{g}}^{\#}\:$are based on have been published elsewhere^35–37,63^. All formulas and coefficients which were used for calculations were either taken from textbook or from literature^33,64,65^ (see Supplemental equations).

### Ion mobility analysis of the T4Ff homo-trimer and its variants

Ion mobility measurements were performed using a Synapt G2-S instrument (Waters Corporation, Wilmslow, UK) equipped with a travelling wave ion mobility (TWIM) cell. Nitrogen was the neutral buffer gas for all TWIM experiments. All mass spectra were acquired in positive ion mode applying a mass window from *m/z* 400 to *m/z* 8000. Instrument settings were as follows: capillary voltage: 1.2 kV, sample cone voltage: 13 V, source offset: 12 V, source temperature: 40 °C, trap collision energy: 2.0 V, pusher: 1900 V, puller: 1370 V, reflectron grid 1.451 kV, source gas flow: 0.0 ml/min, cone gas flow: 9 l/h, nano flow gas pressure: 0.2 bar, helium cell gas flow: 140 ml/min, IMS gas flow: 50.0 ml/min, trap collision voltage: 2 V, trap gas flow: 5.0 ml/min, IMS wave velocity 1600 m/s, wave height: 30.0 V. Data acquisition, arrival time determinations, and processing were done with the MassLynx software version 4.1 (Waters MS-Technologies, Wilmslow, UK^31^. TWIM calibration was done with bovine serum albumin, myoglobin, cytochrome c, ubiquitin, and insulin using published CCS values (Table S18)^66,67^ and applying the published algorithms for logarithmic fits^66,68^ as well as for linear fits^69^.

### In-silico structure analysis of the T4Ff homo-trimer and its variant foldon 1

The T4Ff (foldon 0) atom coordinates (1RF0.pdb) from each chain of the trimer were separated from each other and saved as three independent PDB files which were used as input files for HADDOCK v2.4 (Utrecht, The Netherlands) docking calculations^70^ with keeping the same configurations as in the crystal structure. Similarly, the foldon 1 atom coordinates (2WW6.pdb) from each chain were used as input files. HADDOCK v2.4 clustered 200 structures into one or more clusters, representing either 100% or less of the water-refined models and generated structure model metrics. Note that currently the maximum number of models considered for clustering is 200.

The distances between the Cα atoms of one and the same amino acid in all the three chains of the T4Ff (foldon 0) trimer were determined using ChimeraX v1.8 (2024-06-10, San Francisco, California, USA)^71^. The measured distances were averaged and standard deviations determined. The same procedure was followed for foldon 1. The average distance values for each of the amino acids were compared for estimating similarities and/or differences between foldon 0 and foldon 1 trimers.

The intercatenane and intracatenane interactions (salt bridges, hydrogen bonds, van der Waals forces) were determined for foldon 0 and foldon 1 trimers, respectively, using the Protlnter (protein interaction calculator 0.9.2; Max Planck Institute for Evolutionary Anthropology, Leipzig, Germany) program^72^. A distance of ≤ 4 Å was chosen to determine the amino acid residues which made intercatenane contacts^73^.

The SASAs of foldons 0 and 1 were calculated using an in-house bioinformatics tool, “EpiMED-Surf”^73,74^. The PDB files of the individual trimers were used as input files and the SASAs for each atom, the hydrophobicity of each residue, and the per-atom charge and radius were computed. The differences of the SASAs between foldon 0 and foldon 1 were calculated for all amino acid residues.

The foldon 0 and foldon 1 3D structures were visualized using the UCSF ChimeraX v1.8 molecular visualization software^71^.

## Supplementary Information

Below is the link to the electronic supplementary material.

Supplementary Material 1

## Data Availability

The mass spectrometry data have been deposited to the ProteomeXchange Consortium via the PRIDE partner repository with the dataset identifier PXD064233.

## Abbreviations

a: D-alanine
a.u.: Arbitrary units
f: D-phenylalanine
ASA: Accessible surface area
CID: Collision-induced dissociation
CIU: Collision-induced unfolding
CCS: Collisional cross section
*Δ**CV*: Collisional voltage difference
Dip: 3,3-diphenyl-L-alanine
ESI: Electrospray ionization
ECD: Electron capture dissociation
ETD: Electron transfer dissociation
FWHM: Full width at half maximum
IDR: Intrinsically disordered region
ITEM: Intact transition epitope mapping
ITEM-TWO: ITEM - thermodynamic weak-force order
ITEM-THREE: ITEM - targeted high-energy rupture of extracted epitopes
ITEM-FOUR: ITEM - force differences between original and unusual residues
ITEM-FIVE: ITEM - force interferences by variable extensions
Hot = Tap: Hydroxytheronine-thiaproline bicyclic dipeptide
*m/z*: Mass-to-charge ratio
Nal: 3-(2-Naphthyl)-L-alanine
PDB: Protein database
PRIDE: Proteomics identifications database
Q-ToF: Quadrupole time-of-flight
SID: Surface-induced dissociation
T4Ff: T4 fibritin foldon
TIC: Total ion current
TWIM: Traveling wave ion mobility
UVPD: Ultraviolet photodissociation
wt: Wild-type
