# Supplementary material for: The mass spectrometric intact transition epitope mapping method supports protein engineering of foldon trimer variants
Source: Sci Rep. 2025 Nov 22;15:41467. doi: 10.1038/s41598-025-28101-7 (PMC12644675; doi:10.1038/s41598-025-28101-7)
Supplement: Supplementary file 1 — Supplementary Material 1 [file 41598_2025_28101_MOESM1_ESM.docx]

**The Mass Spectrometric Intact Transition Epitope Mapping Method
Supports Protein Engineering of Foldon Trimer Variants.**

Cornelia Koy ^1)^, Timo Zimmer ^2)^, Kwabena F. M. Opuni ^3)^, Armin Geyer ^2)^, Michael O. Glocker ^1)^

1) Proteome Center Rostock, Medical Faculty and Natural Science Faculty, University of Rostock, Schillingallee 69, 18057 Rostock, Germany

2) Philipps-University Marburg, Department of Chemistry, Hans-Meerwein-Straße 4, 35032 Marburg, Germany

3) Department of Pharmaceutical Chemistry, School of Pharmacy, College of Health Science, University of Ghana, P.O. Box LG43, Legon, Ghana

**Supplement**

Supplemental equations page 1

Supplemental figures page 2

Supplemental tables page 14

# Supplemental Equations

1) **Arrhenius**

$\ln k_{\mathrm{mg}}^{\#}=- \frac{{\Delta G}_{\mathrm{mg}}^{\#}}{R}*\frac{1}{T_{\mathrm{coll}}}+ln A$ (1)

2) **Eyring-Polanyi**

$k_{\mathrm{mg}}^{\#}=\frac{\kappa k_{B} T_{\mathrm{coll}}}{h}{*K}_{D mg}^{\#}$ (2)

3) **van’t Hoff**

${\Delta G}_{\mathrm{mg}}^{\#}=-R T_{\mathrm{coll}}\ln K_{D mg}^{\#}$ (3)

4) **Gibbs-Helmholtz**

${\Delta G}_{\mathrm{mg}}^{\#}={\Delta H}_{\mathrm{mg}}^{\#}- T_{\mathrm{coll}} {\Delta S}_{\mathrm{mg}}^{\#}$ (4)

5) **Conversion of** $\boldsymbol{\Delta CV}$ **to** $\mathbf{T}_{\mathbf{coll}}$ ^33^:

$T_{\mathrm{coll}}={T_{\mathrm{amb}}+\frac{x m e}{3N_{\mathrm{at}}k_{B}}*\Delta CV}$ (5)

6) **Law of mass action**

$K_{D mg}^{\#}=\frac{f(products)}{f(educts)}$ (6)

$f(products)=\frac{h(products)}{h(total)}$ ∗ 100% (7)

ℎ(products)+ℎ(educts) = ℎ(total) ≙ 100 % (8)

7) **Boltzmann fit equation**


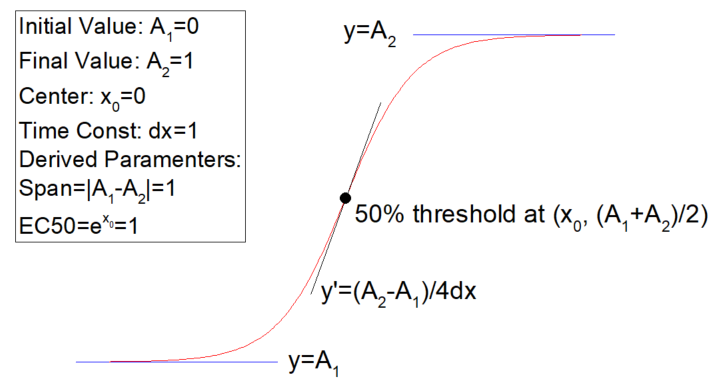


$y=\frac{A_{1}-A_{2}}{1+ e^{\frac{(x-x_{0})}{dx}}}+A_{2}$ (9)

equation of the tangent line:

y = b ∗ x + c (10)

Slope of tangent line:

$b=\frac{A_{2}-A_{1}}{4dx}$ (11)

Intercept of the tangent line with the y – axis:

$c=\frac{A_{1}+A_{2}}{2}$ - $\frac{A_{1}-A_{2}}{4dx}$ ∗ $x_{0}$ (12)

**Symbols**

$T_{\mathrm{coll}}$ : protein-ligand temperature during collision in the collision cell

$T_{\mathrm{amb}}$ : absolute ambient temperature, 298 K

$R$ : gas constant

$h$ : Planck’s constant

$\kappa$ : transmission coefficient; is equal to 1 as the products do not re-cross the transition state

$x$ : conversion factor

$=$0.24 for proteins ^64^

$=$ 0.9 for peptides ^65^

$m$ : abundance weighted mean charge state of multiply charged and accelerated protein - ligand complex ions

$e$ : fundamental charge

$N_{\mathrm{at}}$ number of atoms in the complex

$k_{B}$ : Boltzmann constant

$\Delta CV$ : collision cell voltage difference

ℎ(products) : height of apex of Gaussian fit of multiply charged product ions

ℎ(educts) : height of apex of Gaussian fit of multiply charged educt ions

# Supplement Figures


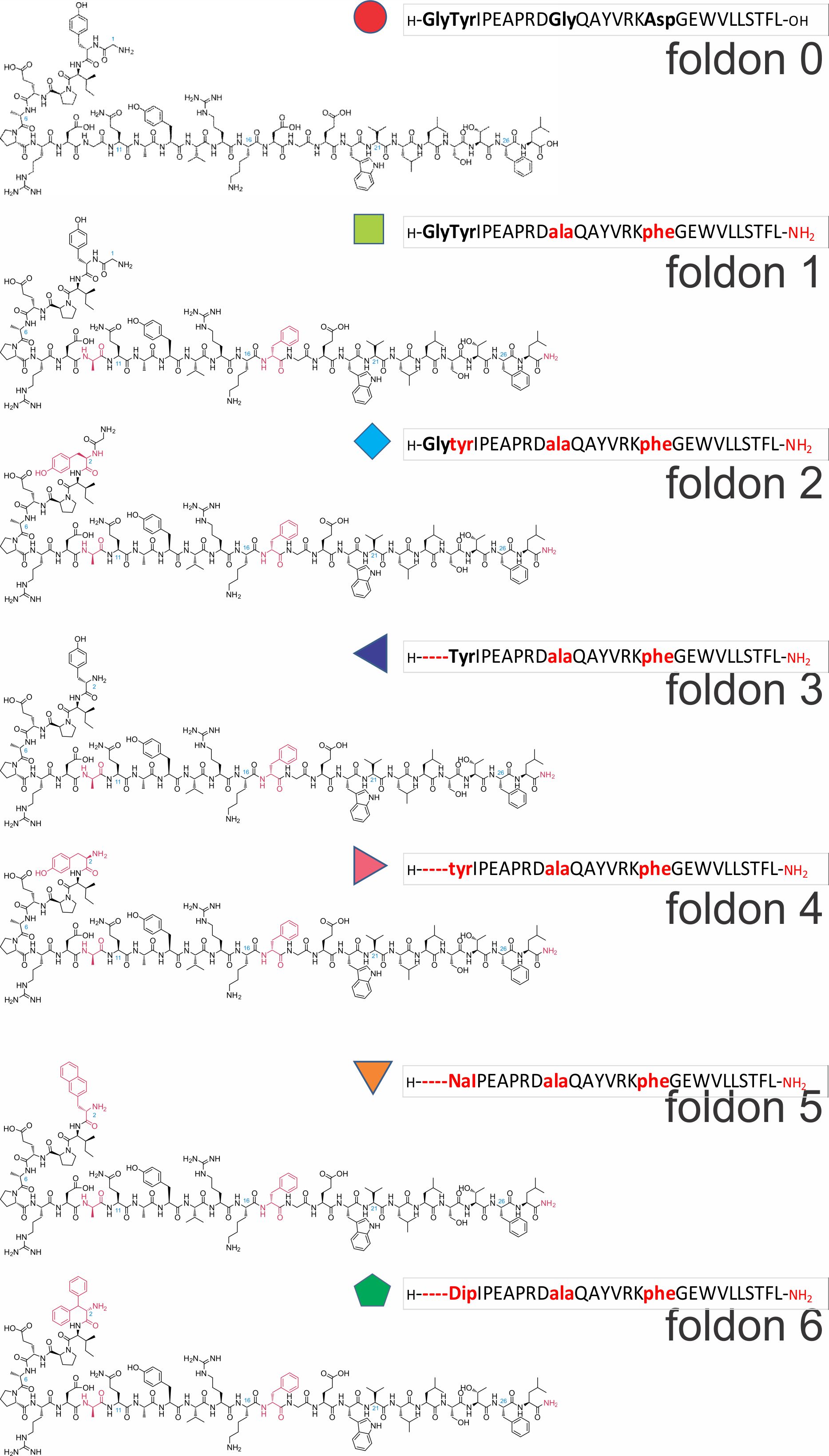


**Figure S1:** Chemical structures of T4Ff foldon and synthetic variants. Amino acid sequences are written in single letter code except for amino acid residues which are altered in the synthetic foldons. Exchanged amino acids are printed in red and bold and in the three letter code. D-amino acid residues are written in small letters.


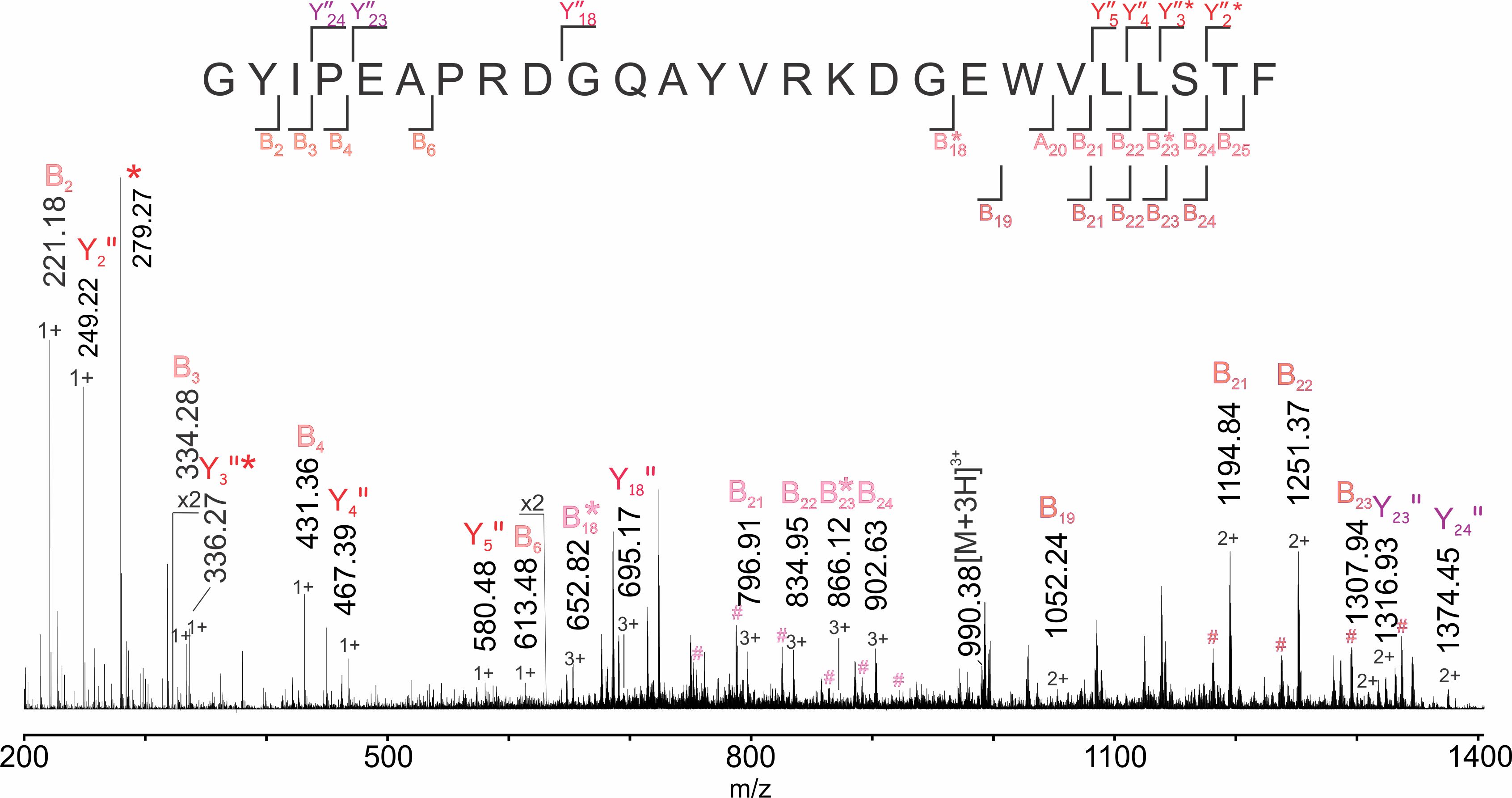


**Figure S2:** MS/MS spectrum of truncated foldon 0. The triply protonated molecular ion at *m/z* 990.38 served as precursor ion (cf. Figure 1). Selected ion signals are labeled. Y’’-type ions and B-type fragment ions are shown. # indicates A-type ions, * labels ion signals with loss of H_2_O. The insert shows the deduced amino acid sequence of the precursor in single letter code as well as locations of peptide bond breaks.


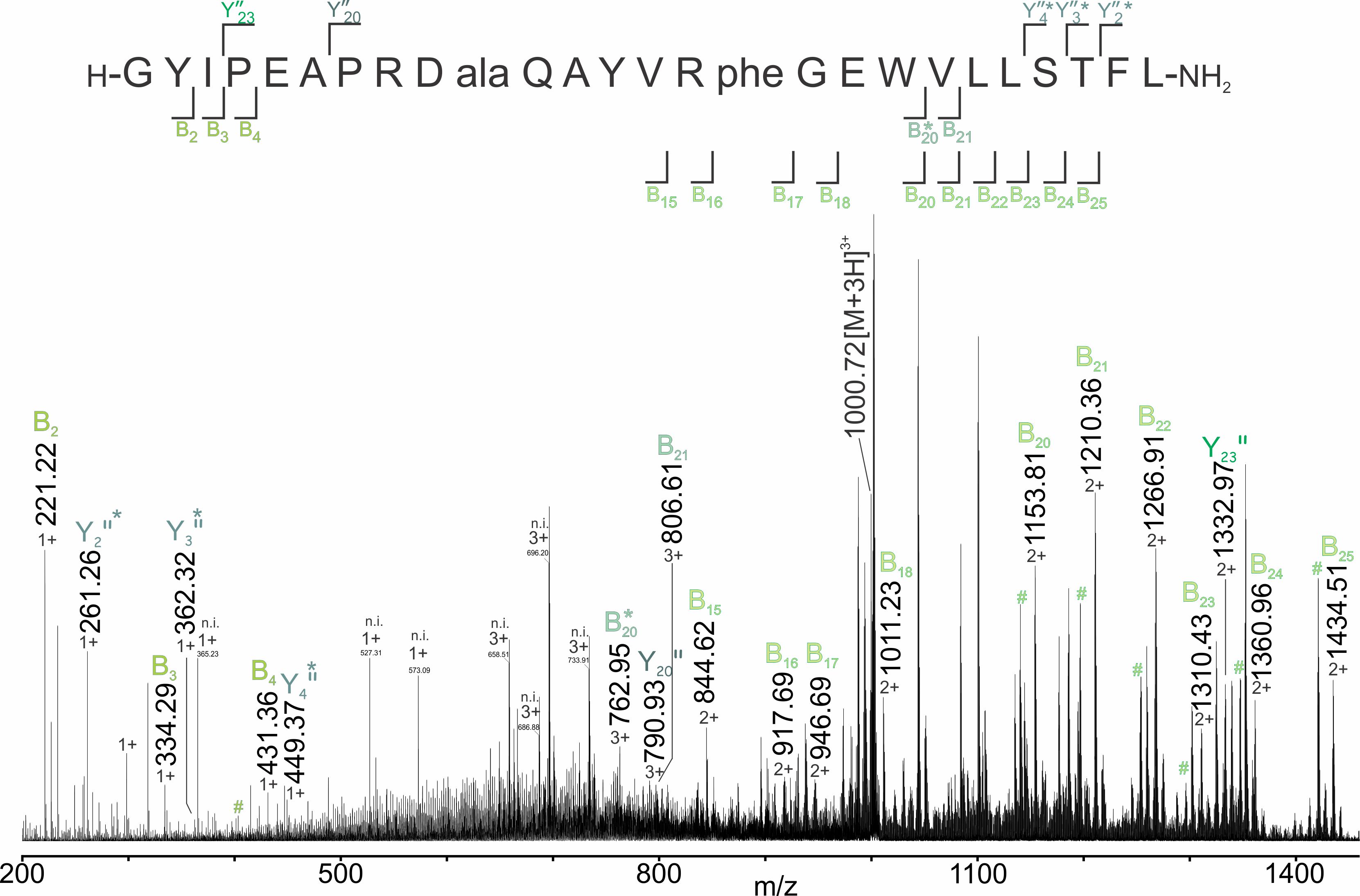


**Figure S3:** MS/MS spectrum of truncated foldon 1. The triply protonated molecular ion at *m/z* 1000.72 served as precursor ion (cf. Figure 1). Selected ion signals are labeled. Y’’-type ions and B-type fragment ions are shown. # indicates A-type ions, * labels ion signals with loss of H_2_O, n.i.: not identified. The insert shows the deduced amino acid sequence of the precursor in single letter code as well as locations of peptide bond breaks.


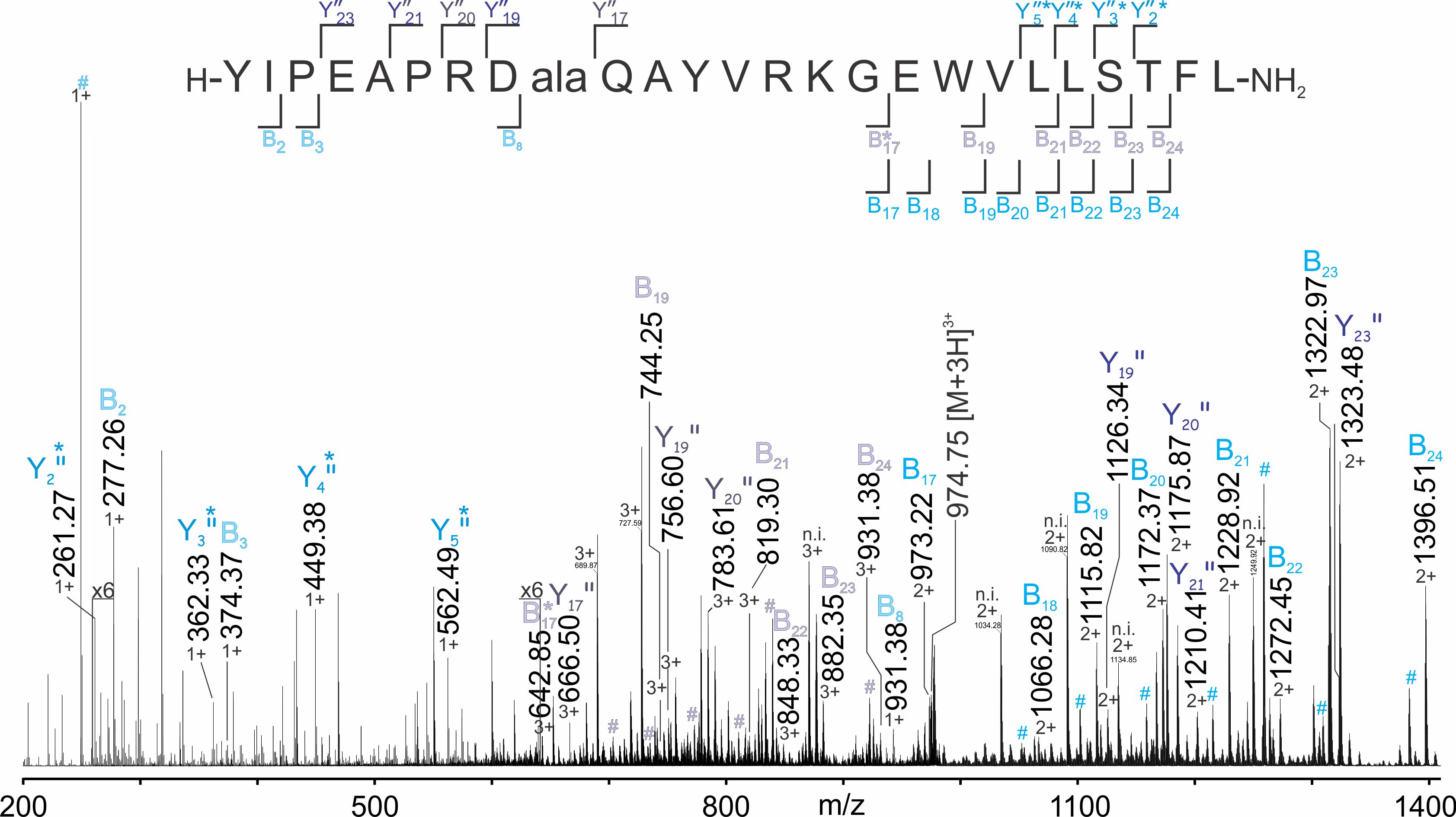


**Figure S4:** MS/MS spectrum of truncated foldon 3. The triply protonated molecular ion at *m/z* 974.75 served as precursor ion (cf. Figure 1). Selected ion signals are labeled. Y’’-type ions and B-type fragment ions are shown. # indicates A-type ions, * labels ion signals with loss of H_2_O, n.i.: not identified. The insert shows the deduced amino acid sequence of the precursor in single letter code as well as locations of peptide bond breaks.


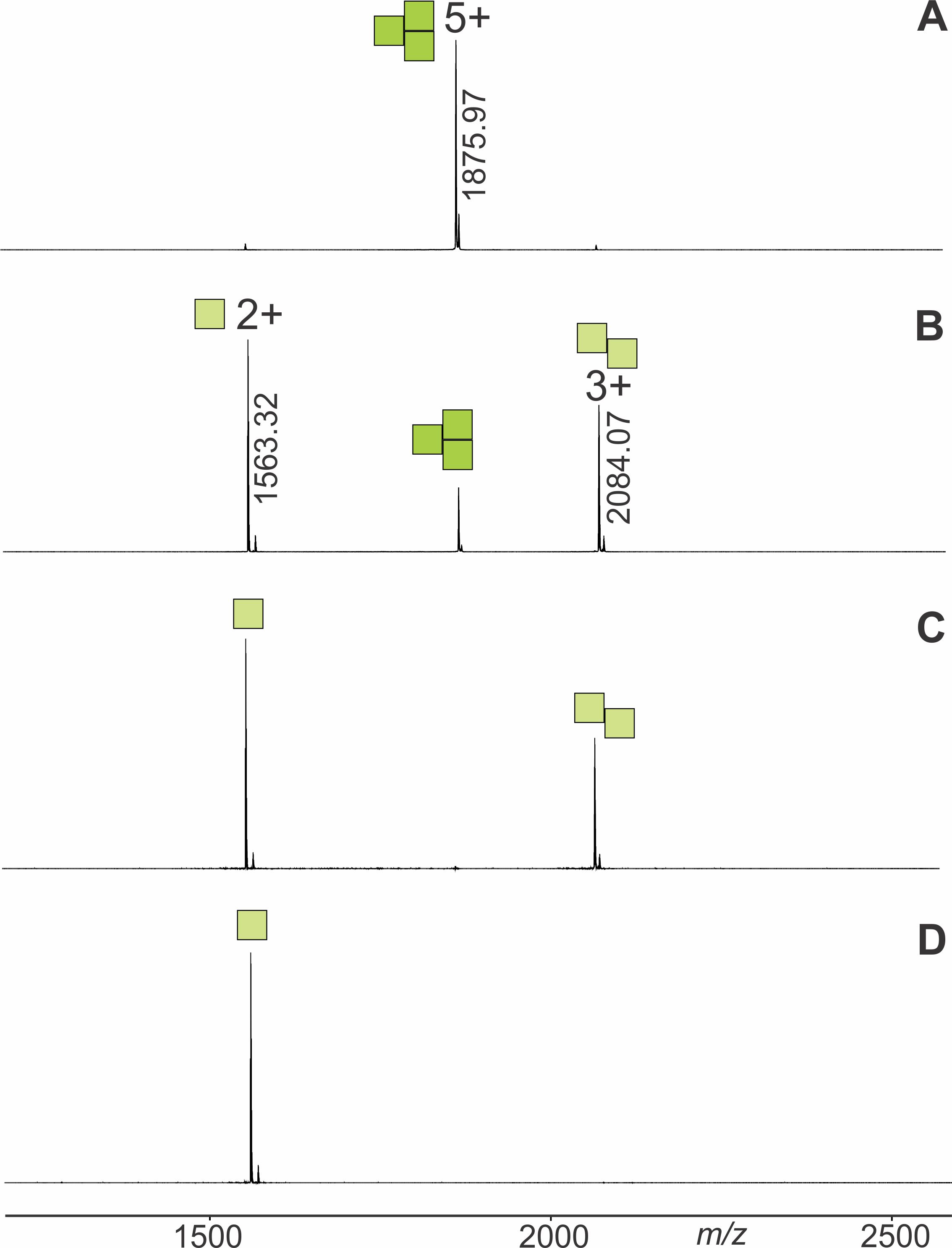


**Figure S5:** Nano-ESI mass spectra of foldon 1 with increasing collision cell voltage differences (∆CV). **A:** 0 V, **B:** 15 V, **C:** 20 V, **D:** 40 V. Charge states and *m/z* values of ion signals are given. The quadrupole was set to isolate the 5+ trimer ion. Symbols indicate multimeric states. Solvent: 50 mM ammonium acetate, pH 5.5.


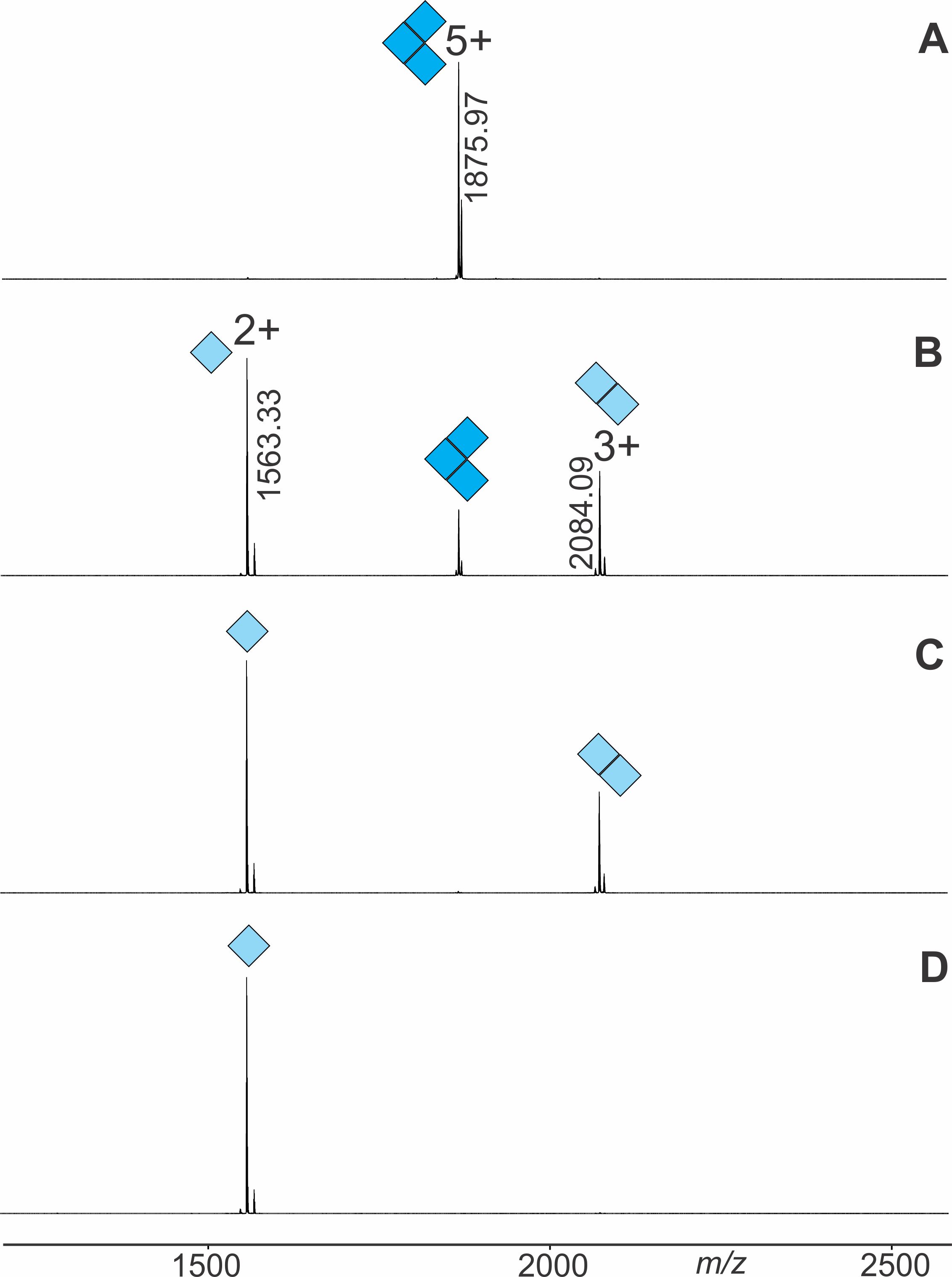


**Figure S6:** Nano-ESI mass spectra of foldon 2 with increasing collision cell voltage differences (∆CV). **A:** 0 V, **B:** 15 V, **C:** 20 V, **D:** 40 V. Charge states and *m/z* values of ion signals are given. The quadrupole was set to isolate the 5+ trimer ion. Symbols indicate multimeric states. Solvent: 50 mM ammonium acetate, pH 5.5.


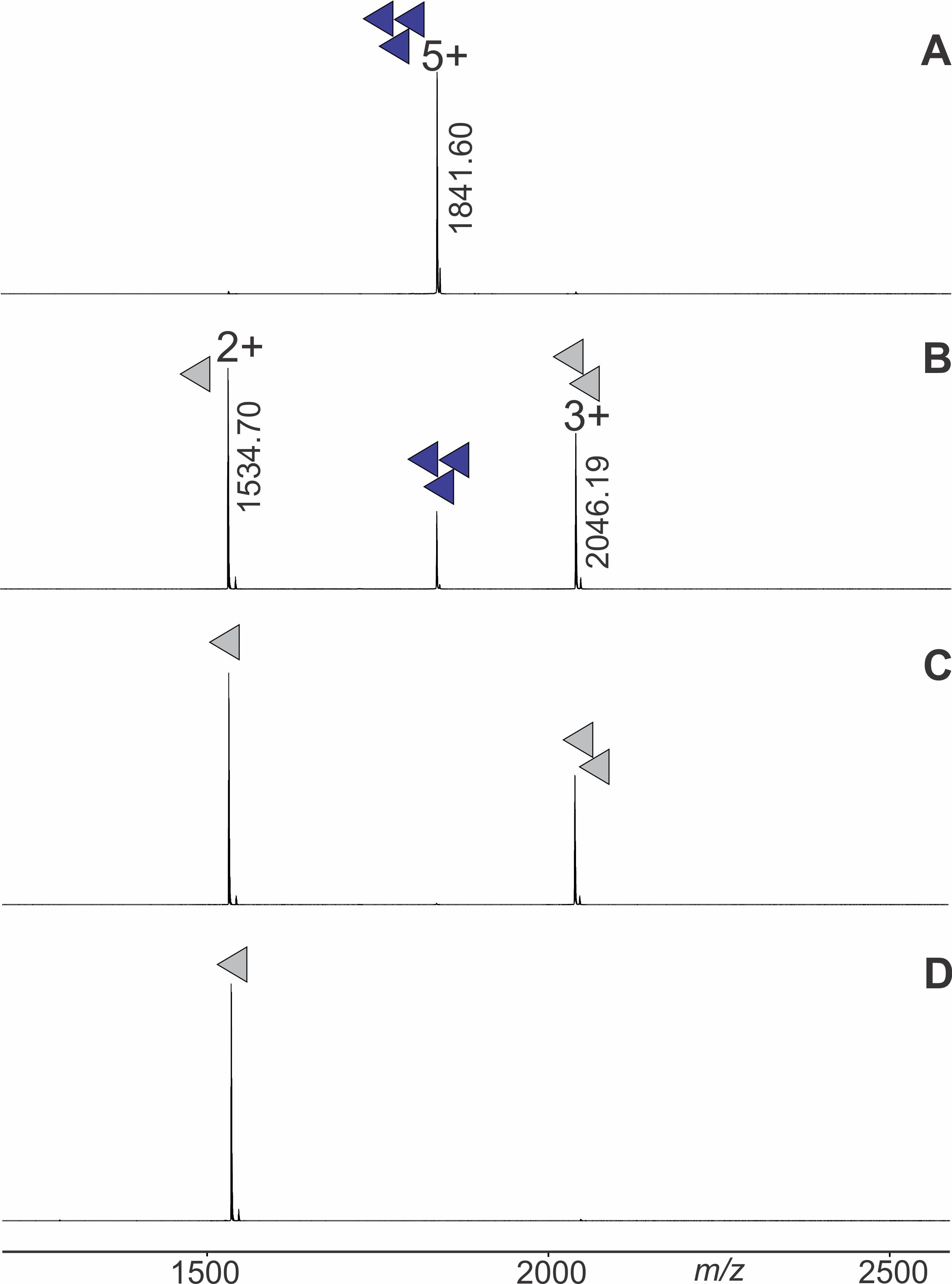


**Figure S7:** Nano-ESI mass spectra of foldon 3 with increasing collision cell voltage differences (∆CV). **A:** 0 V, **B:** 15 V, **C:** 20 V, **D:** 30 V. Charge states and *m/z* values of ion signals are given. The quadrupole was set to isolate the 5+ trimer ion. Symbols indicate multimeric states. Solvent: 50 mM ammonium acetate, pH 5.5.


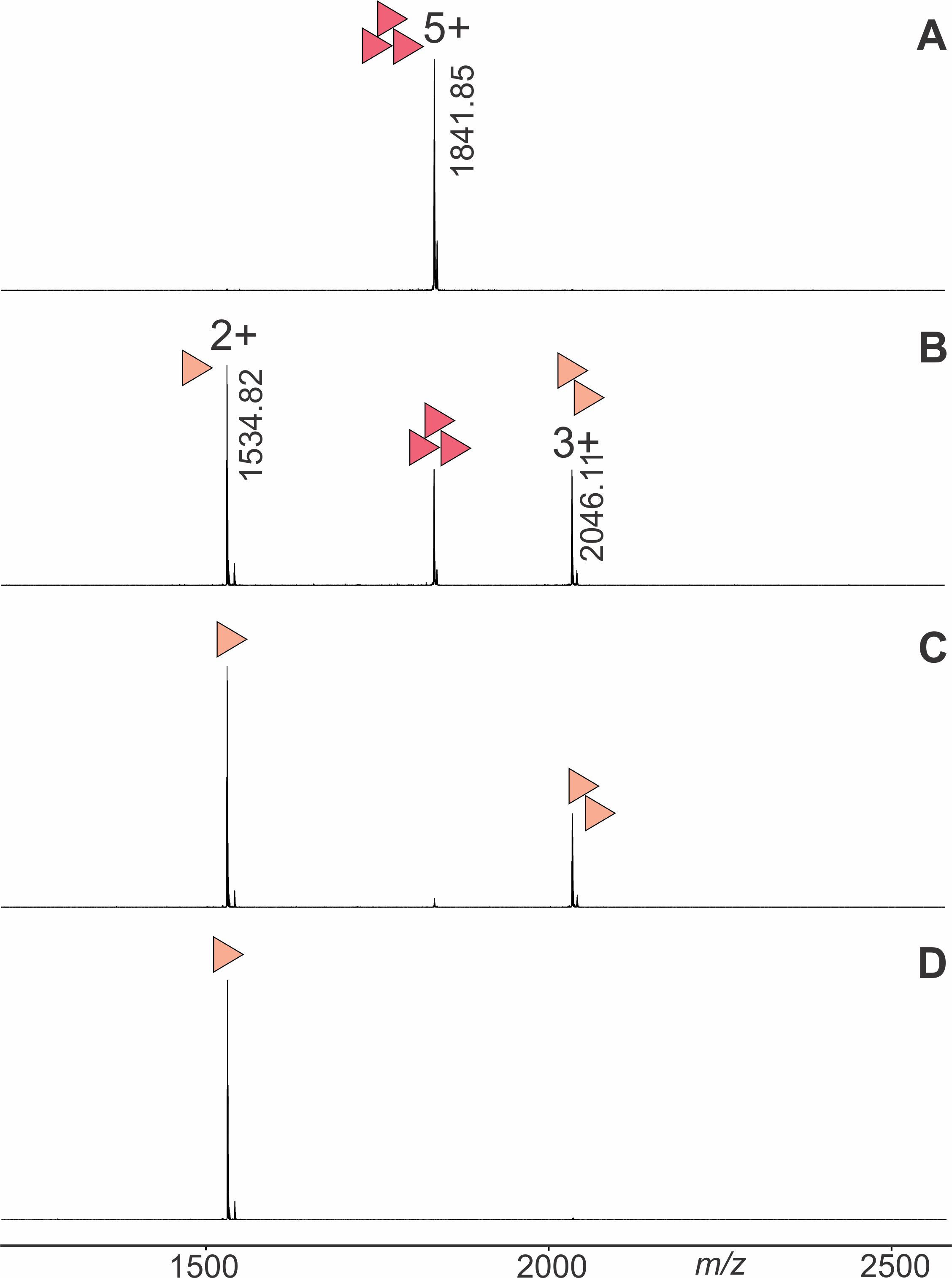


**Figure S8:** Nano-ESI mass spectra of foldon 4 with increasing collision cell voltage differences (∆CV). **A:** 2 V, **B:** 15 V, **C:** 20 V, **D:** 40 V. Charge states and *m/z* values of ion signals are given. The quadrupole was set to isolate the 5+ trimer ion. Symbols indicate multimeric states. Solvent: 50 mM ammonium acetate, pH 5.5.


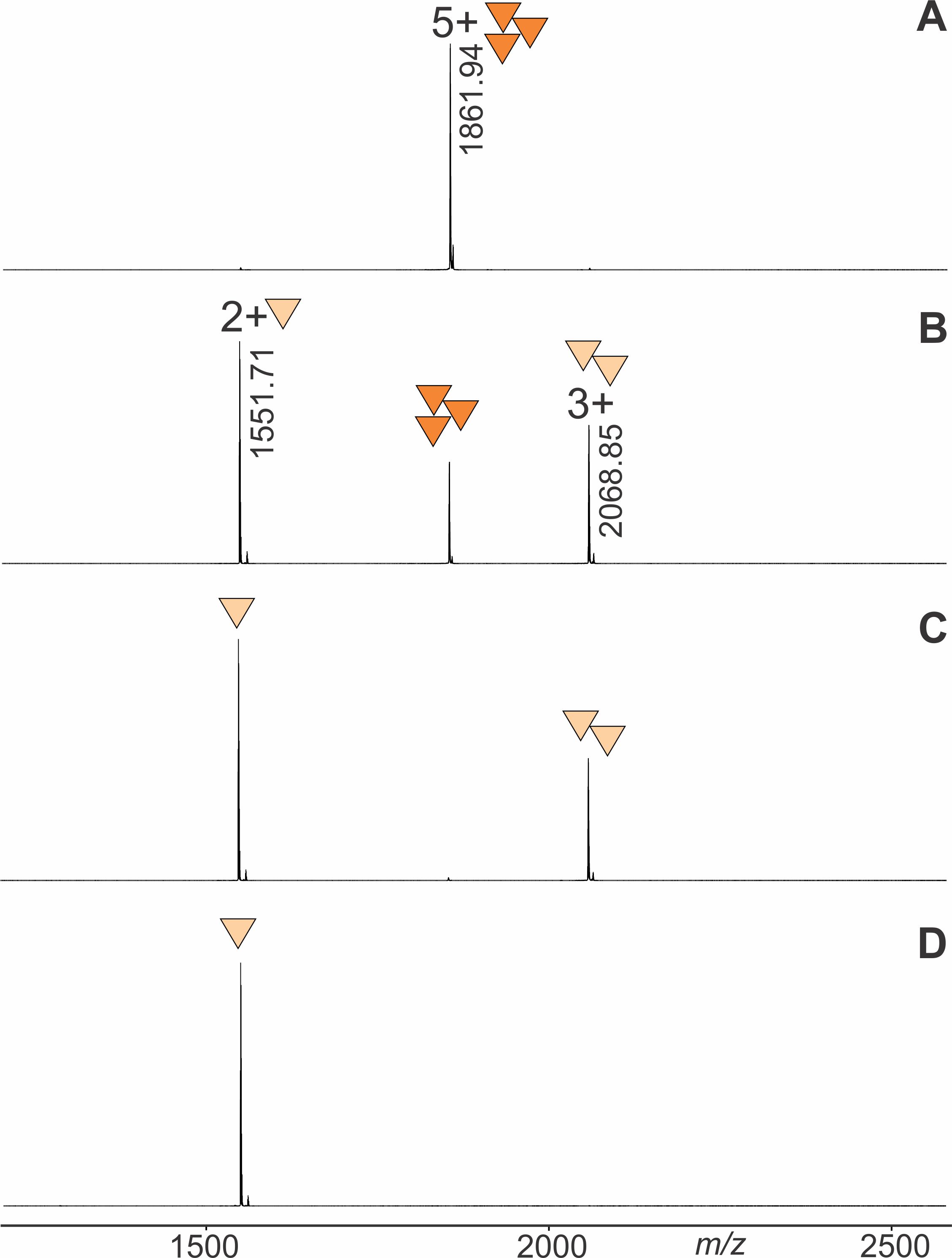


**Figure S9:** Nano-ESI mass spectra of foldon 5 with increasing collision cell voltage differences (∆CV). **A:** 2 V, **B:** 15 V, **C:** 20 V, **D:** 40 V. Charge states and *m/z* values of ion signals are given. The quadrupole was set to isolate the 5+ trimer ion. Symbols indicate multimeric states. Solvent: 50 mM ammonium acetate, pH 5.5.


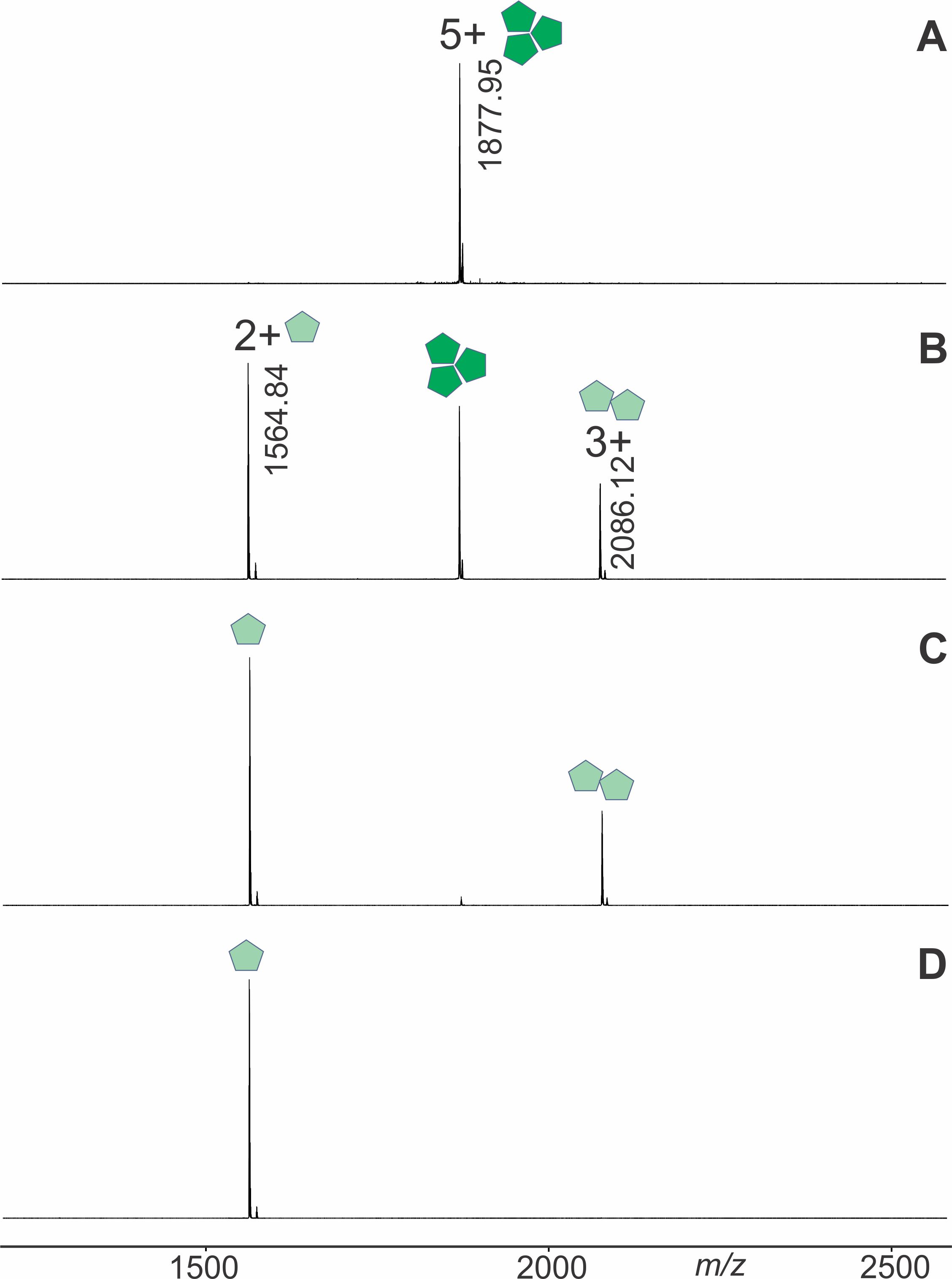


**Figure S10:** Nano-ESI mass spectra of foldon 6 with increasing collision cell voltage differences (∆CV). **A:** 2 V, **B:** 15 V, **C:** 20 V, **D:** 40 V. Charge states and *m/z* values of ion signals are given. The quadrupole was set to isolate the 5+ trimer ion. Symbols indicate multimeric states. Solvent: 50 mM ammonium acetate, pH 5.5.


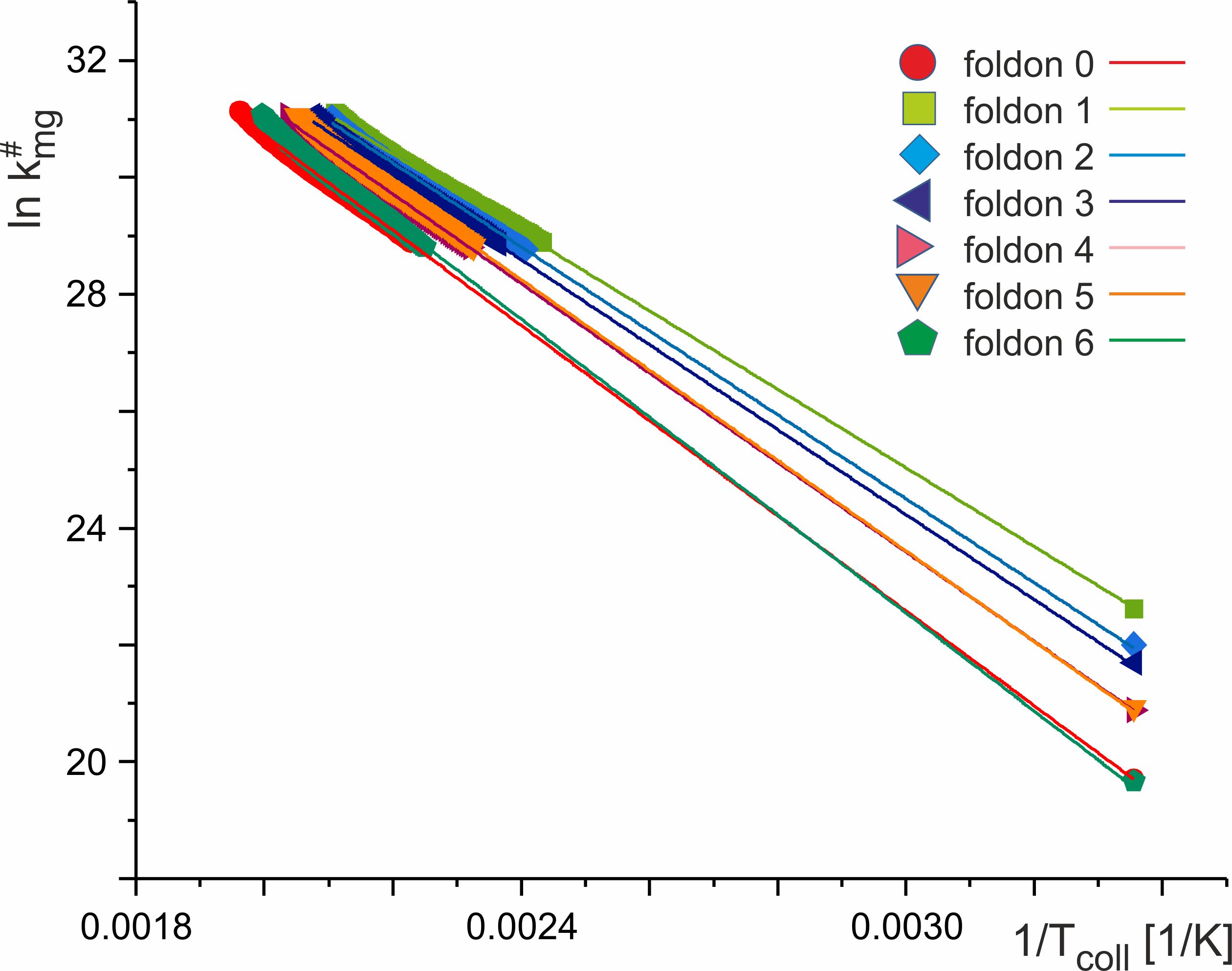


**Figure S11:** Arrhenius plot for the foldon trimer dissociation reactions in the gas phase. Foldon 0 (red circle), foldon 1 (green square), foldon 2 (blue diamond), foldon 3 (dark blue triangle), foldon 4 (pink triangle), foldon 5 (orange triangle), foldon 6 (dark green pentagon). Each data point (thickened parts of the lines) has been obtained experimentally and corresponding lines have been extrapolated linearly. The values for ${\boldsymbol{ln}\mathbf{k}}_{\mathbf{m0g}}^{\boldsymbol{\#}}$ are read at $\mathbf{1/T}_{\mathbf{amb}}$..


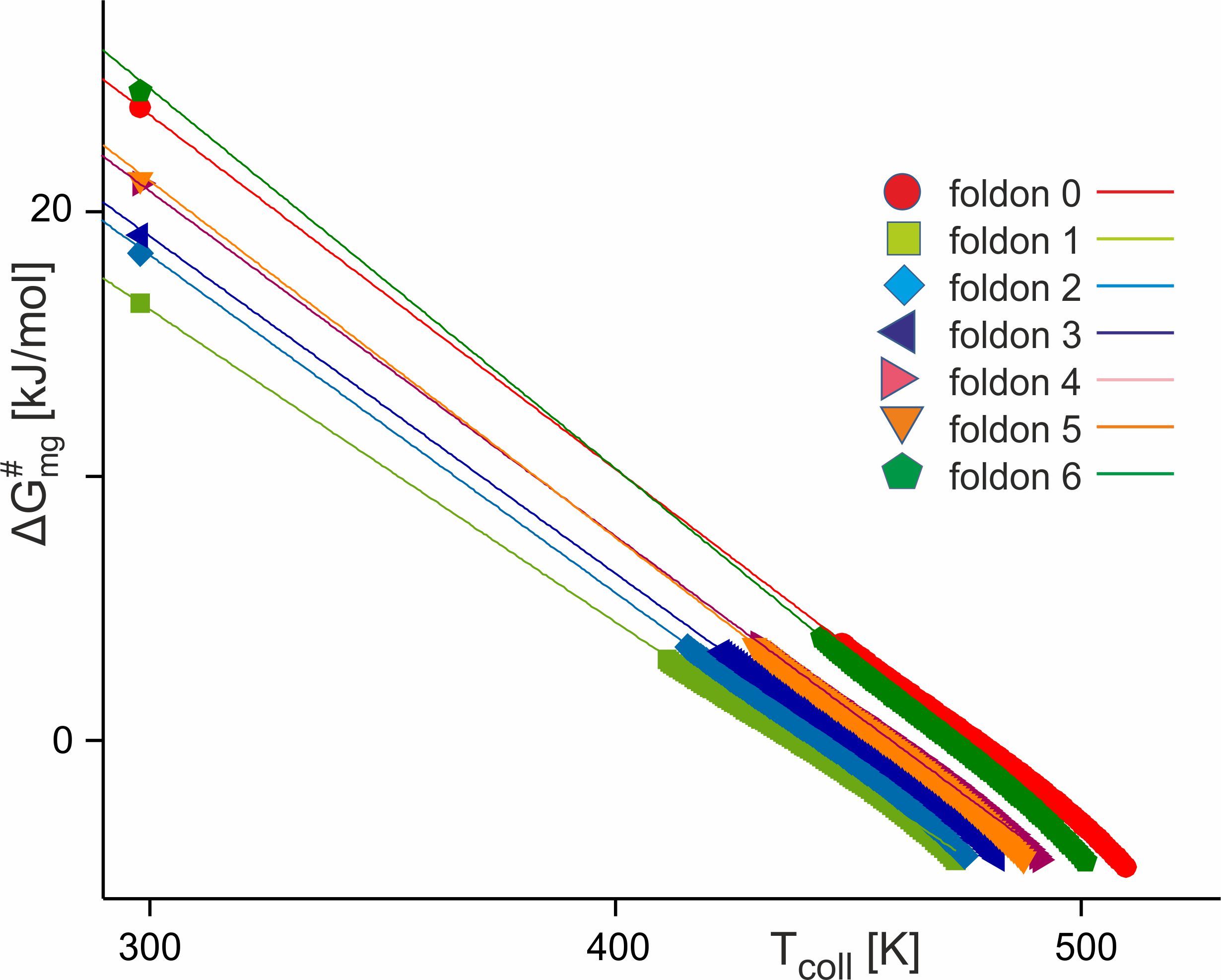


**Figure S12:** Ellingham diagram for the foldon trimer dissociation reactions in the gas phase. Foldon 0 (red circle), foldon 1 (green square), foldon 2 (blue diamond), foldon 3 (dark blue triangle), foldon 4 (pink triangle), foldon 5 (orange triangle), foldon 6 (dark green pentagon). Each data point (thickened parts of the lines) has been obtained experimentally and corresponding lines have been extrapolated linearly. The values for $\boldsymbol{\Delta G}_{\mathbf{m0g}}^{\boldsymbol{\#}}$ are read at $\mathbf{T}_{\mathbf{amb}}$.


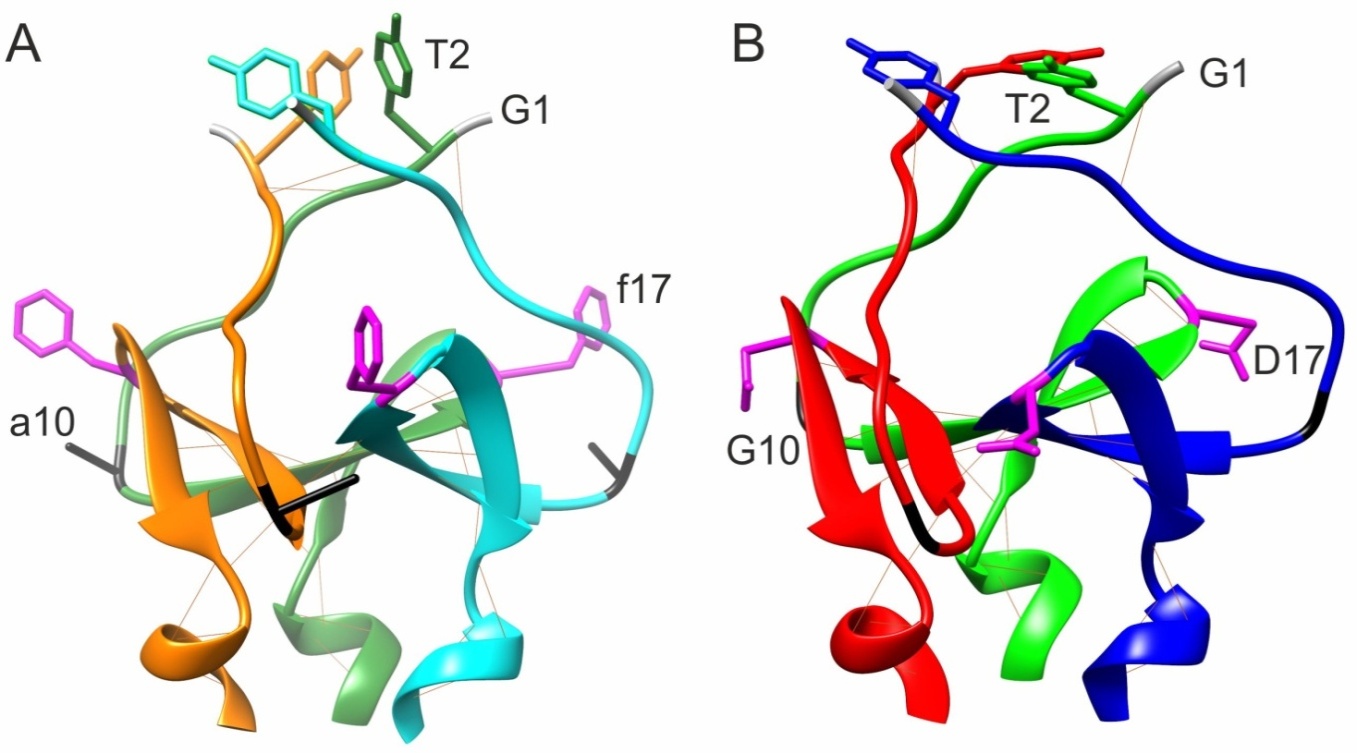


**Figure S13:** Cartoon views of ribbon diagrams of foldon 3D structures. **A**: foldon 1 (2ww6.pdb). B: wild type foldon 0 (1rfo.pdb). Side chains of selected amino acid residues are shown as stick models. Selected amino acid residues of one of the foldon monomers (green chains) are labelled using the single letter code. D-amino acid residues are represented by small letters. Numbers give amino acid residue positions. Thin lines show hydrogen bridges.


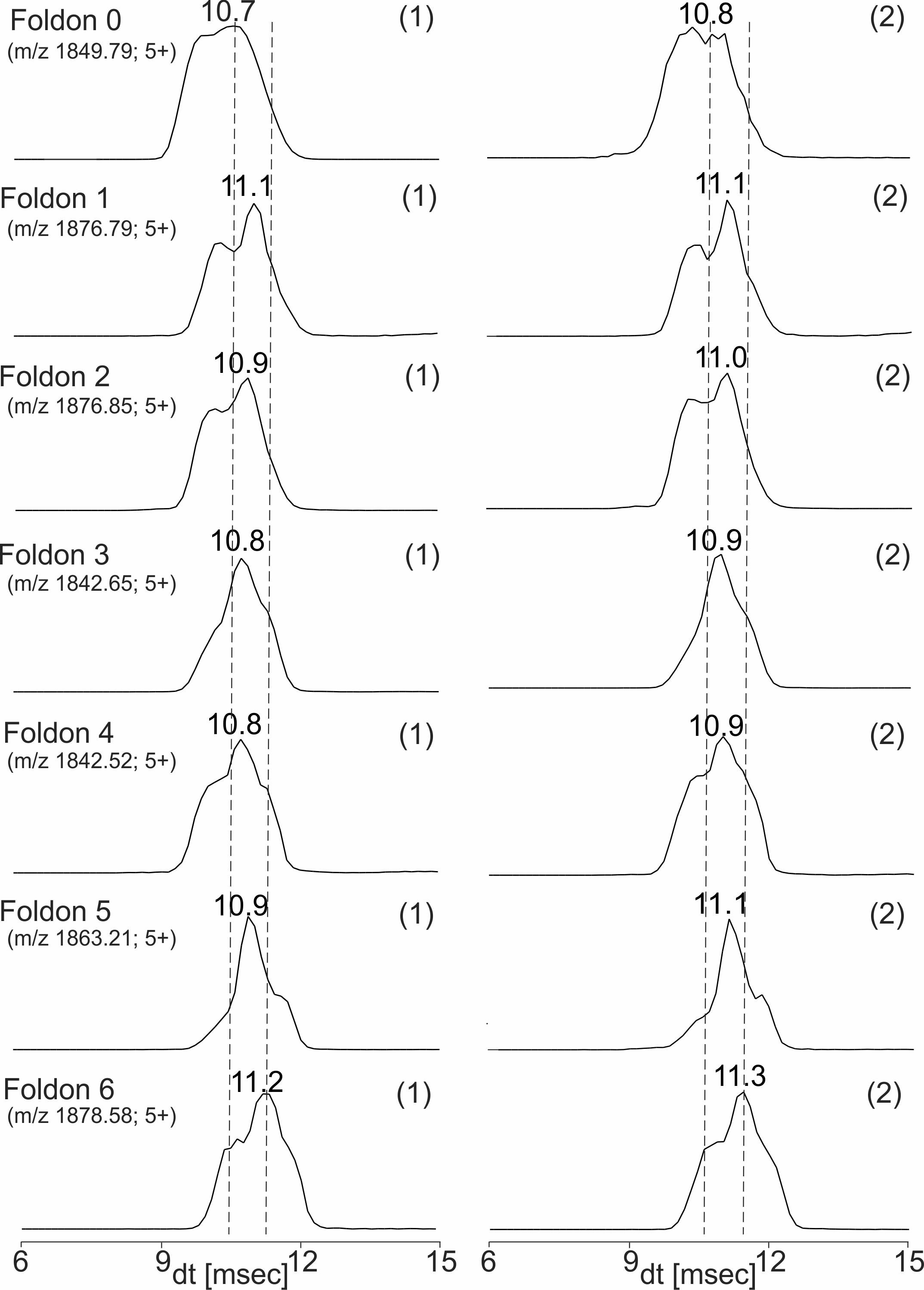


**Figure S14:** Drift time traces of quintuply protonated foldon trimer ion signals. Left panels (1): first measurement. Right panels (2): second measurement. Vertical lines indicate 10.7 msec (left) and the 11.3 msec (right) positions.

# Supplement Tables

**Table S1:** Ion intensities, charge states, and *m/z* values for the foldon 0 homo-trimer at various collision cell voltage difference settings

**1st determination (Foldon_0_02a.raw)**

| ion | *z* | 0V | 2V | 5V | 7V | 10V | 12V | 15V | 20V | 30V | 40V | 50V |
| --- | --- | --- | --- | --- | --- | --- | --- | --- | --- | --- | --- | --- |
| trimer | 5+ | 688000 | 1200000 | 869000 | 938000 | 750000 | 674000 | 399000 | 33500 | 0 | - | 0 |
| dimer | 3+ | 5760 | 11000 | 18300 | 37200 | 81100 | 142000 | 310000 | 498000 | 224000 | - | 0 |
| monomer | 2+ | 7720 | 17100 | 25500 | 50800 | 108000 | 185000 | 394000 | 774000 | 870000 | - | 1250000 |

**2nd determination (Foldon_0_04a.raw)**

| ion | *z* | 0V | 2V | 5V | 7V | 10V | 12V | 15V | 20V | 30V | 40V | 50V |
| --- | --- | --- | --- | --- | --- | --- | --- | --- | --- | --- | --- | --- |
| trimer | 5+ | 674000 | 897000 | 910000 | 1000000 | 853000 | 756000 | 414000 | 26800 | 0 | 0 | 0 |
| dimer | 3+ | 5600 | 11400 | 26000 | 50000 | 106000 | 198000 | 378000 | 564000 | 279000 | 11000 | 0 |
| monomer | 2+ | 9090 | 17100 | 33700 | 64100 | 139000 | 253000 | 487000 | 820000 | 1110000 | 1510000 | 1350000 |

**Table S2:** Ion intensities, charge states, and *m/z* values for the foldon 1 homo-trimer at various collision cell voltage difference settings

**1st determination (Foldon_1_02a.raw)**

| ion | *z* | 0V | 2V | 5V | 7V | 10V | 12V | 15V | 20V | 30V | 40V | 50V |
| --- | --- | --- | --- | --- | --- | --- | --- | --- | --- | --- | --- | --- |
| trimer | 5+ | 526000 | 580000 | 429000 | 494000 | 250000 | 225000 | 86900 | 4310 | 0 | 0 | 0 |
| dimer | 3+ | 12800 | 19800 | 30600 | 56300 | 77100 | 148000 | 199000 | 219000 | 53200 | 0 | 0 |
| monomer | 2+ | 15700 | 24400 | 35100 | 74500 | 104000 | 198000 | 287000 | 386000 | 530000 | 684000 | 532000 |

**2nd determination (Foldon_1_04a.raw)**

| ion | *z* | 0V | 2V | 5V | 7V | 10V | 12V | 15V | 20V | 30V | 40V | 50V |
| --- | --- | --- | --- | --- | --- | --- | --- | --- | --- | --- | --- | --- |
| trimer | 5+ | 196000 | 334000 | 378000 | 367000 | 294000 | 190000 | 76900 | 2360 | 0 | 0 | 0 |
| dimer | 3+ | 5480 | 12000 | 25000 | 43200 | 91600 | 128000 | 200000 | 224000 | 61200 | 1750 | 0 |
| monomer | 2+ | 6810 | 15200 | 34000 | 56800 | 121000 | 183000 | 294000 | 413000 | 630000 | 482000 | 506000 |

**Table S3:** Ion intensities, charge states, and *m/z* values for the foldon 2 homo-trimer at various collision cell voltage difference settings

**1st determination (Foldon_2_03a.raw)**

| ion | *z* | 0V | 2V | 5V | 7V | 10V | 12V | 15V | 20V | 30V | 40V | 50V |
| --- | --- | --- | --- | --- | --- | --- | --- | --- | --- | --- | --- | --- |
| trimer | 5+ | 98500 | 114000 | 128000 | 138000 | 122000 | 102000 | 49400 | 1940 | 0 | 0 | 0 |
| dimer | 3+ | 571 | 1070 | 3630 | 7300 | 22800 | 42000 | 78100 | 111000 | 51100 | 3350 | 0 |
| monomer | 2+ | 886 | 2030 | 5450 | 11800 | 42600 | 80300 | 162000 | 256000 | 475000 | 670000 | 789000 |

**2nd determination (Foldon_2_05a.raw)**

| ion | *z* | 0V | 2V | 5V | 7V | 10V | 12V | 15V | 20V | 30V | 40V | 50V |
| --- | --- | --- | --- | --- | --- | --- | --- | --- | --- | --- | --- | --- |
| trimer | 5+ | 153000 | 186000 | 196000 | 226000 | 216000 | 181000 | 77100 | 2710 | 0 | 0 | 0 |
| dimer | 3+ | 1540 | 2430 | 7080 | 18900 | 53600 | 104000 | 172000 | 251000 | 88600 | 0 | 0 |
| monomer | 2+ | 1600 | 4040 | 13500 | 35100 | 93300 | 171000 | 304000 | 475000 | 864000 | 1230000 | 1420000 |

**Table S4:** Ion intensities, charge states, and *m/z* values for the foldon 3 homo-trimer at various collision cell voltage difference settings

**1st determination (Foldon_3_02a.raw)**

| ion | *z* | 0V | 2V | 5V | 7V | 10V | 12V | 15V | 20V | 30V | 40V | 50V |
| --- | --- | --- | --- | --- | --- | --- | --- | --- | --- | --- | --- | --- |
| trimer | 5+ | 1450000 | 713000 | 720000 | 590000 | 562000 | 366000 | 129000 | 5020 | 0 | 0 | 0 |
| dimer | 3+ | 14100 | 11100 | 27100 | 43300 | 117000 | 178000 | 257000 | 398000 | 61600 | 8530 | 0 |
| monomer | 2+ | 18600 | 16500 | 37800 | 56900 | 160000 | 235000 | 365000 | 711000 | 720000 | 1080000 | 1140000 |

**2nd determination (Foldon_3_05a.raw)**

| ion | *z* | 0V | 2V | 5V | 7V | 10V | 12V | 15V | 20V | 30V | 40V | 50V |
| --- | --- | --- | --- | --- | --- | --- | --- | --- | --- | --- | --- | --- |
| trimer | 5+ | 728000 | 823000 | 773000 | 759000 | 607000 | 441000 | 172000 | 9690 | 0 | 0 | 0 |
| dimer | 3+ | 9790 | 15900 | 36900 | 68400 | 154000 | 243000 | 351000 | 401000 | 91600 | 5180 | 0 |
| monomer | 2+ | 15100 | 25300 | 49300 | 85600 | 198000 | 314000 | 515000 | 703000 | 1070000 | 1160000 | 997000 |

**Table S5:** Ion intensities, charge states, and *m/z* values for the foldon 4 homo-trimer at various collision cell voltage difference settings

**1st determination (Foldon_4_02a.raw)**

| ion | *z* | 0V | 2V | 5V | 7V | 10V | 12V | 15V | 20V | 30V | 40V | 50V |
| --- | --- | --- | --- | --- | --- | --- | --- | --- | --- | --- | --- | --- |
| trimer | 5+ | 52400 | 68500 | 79400 | 66300 | 56700 | 49800 | 25800 | 3170 | 0 | 0 | 0 |
| dimer | 3+ | 258 | 868 | 1510 | 2720 | 6800 | 13100 | 25700 | 33000 | 13400 | 1320 | 0 |
| monomer | 2+ | 483 | 851 | 2640 | 4360 | 13400 | 24600 | 49000 | 84600 | 137000 | 158000 | 140000 |

**2nd determination (Foldon_4_04a.raw)**

| ion | *z* | 0V | 2V | 5V | 7V | 10V | 12V | 15V | 20V | 30V | 40V | 50V |
| --- | --- | --- | --- | --- | --- | --- | --- | --- | --- | --- | --- | --- |
| trimer | 5+ | 108000 | 144000 | 146000 | 143000 | 128000 | 100000 | 48800 | 2520 | 0 | 0 | 0 |
| dimer | 3+ | 593 | 1560 | 3440 | 6610 | 21700 | 36500 | 66900 | 89400 | 34300 | 2460 | 0 |
| monomer | 2+ | 1020 | 2300 | 6150 | 12900 | 40000 | 63500 | 130000 | 210000 | 309000 | 336000 | 316000 |

**Table S6:** Ion intensities, charge states, and *m/z* values for the foldon 5 homo-trimer at various collision cell voltage difference settings

**1st determination (Foldon_5_02a.raw)**

| ion | *z* | 0V | 2V | 5V | 7V | 10V | 12V | 15V | 20V | 30V | 40V | 50V |
| --- | --- | --- | --- | --- | --- | --- | --- | --- | --- | --- | --- | --- |
| trimer | 5+ | 31100 | 69400 | 104000 | 115000 | 128000 | 119000 | 84800 | 7660 | 0 | 0 | 0 |
| dimer | 3+ | 94 | 506 | 764 | 2280 | 9710 | 18400 | 46800 | 81400 | 36800 | 0 | 0 |
| monomer | 2+ | 126 | 279 | 1330 | 4040 | 15500 | 38700 | 106000 | 213000 | 353000 | 397000 | 354000 |

**2nd determination (Foldon_5_04a.raw)**

| ion | *z* | 0V | 2V | 5V | 7V | 10V | 12V | 15V | 20V | 30V | 40V | 50V |
| --- | --- | --- | --- | --- | --- | --- | --- | --- | --- | --- | --- | --- |
| trimer | 5+ | 172000 | 215000 | 235000 | 217000 | 174000 | 140000 | 81700 | 5220 | 0 | 0 | 0 |
| dimer | 3+ | 535 | 0 | 2490 | 5760 | 14600 | 29100 | 53600 | 77600 | 26000 | 0 | 0 |
| monomer | 2+ | 1250 | 1680 | 5090 | 10700 | 30500 | 56500 | 123000 | 202000 | 301000 | 313000 | 275000 |

**Table S7:** Ion intensities, charge states, and *m/z* values for the foldon 6 homo-trimer at various collision cell voltage difference settings

**1st determination (Foldon_6_02a.raw)**

| ion | *z* | 0V | 2V | 5V | 7V | 10V | 12V | 15V | 20V | 30V | 40V | 50V |
| --- | --- | --- | --- | --- | --- | --- | --- | --- | --- | --- | --- | --- |
| trimer | 5+ | 1400000 | 1190000 | 820000 | 668000 | 619000 | 474000 | 195000 | 8180 | 0 | 0 | 0 |
| dimer | 3+ | 11900 | 13100 | 23500 | 35700 | 93500 | 167000 | 266000 | 309000 | 75600 | 0 | 0 |
| monomer | 2+ | 16600 | 18600 | 33100 | 52800 | 141000 | 248000 | 426000 | 610000 | 830000 | 919000 | 851000 |

**2nd determination (Foldon_6_04a.raw)**

| ion | *z* | 0V | 2V | 5V | 7V | 10V | 12V | 15V | 20V | 30V | 40V | 50V |
| --- | --- | --- | --- | --- | --- | --- | --- | --- | --- | --- | --- | --- |
| trimer | 5+ | 340000 | 433000 | 444000 | 406000 | 340000 | 237000 | 103000 | 3540 | 0 | 0 | 0 |
| dimer | 3+ | 1940 | 4030 | 9770 | 21500 | 51600 | 80600 | 129000 | 150000 | 49600 | 0 | 0 |
| monomer | 2+ | 3530 | 8170 | 18000 | 33400 | 90300 | 138000 | 241000 | 347000 | 517000 | 533000 | 467000 |

**Table S8:** Boltzmann curve parameters of foldon trimer dissociation reactions in the gas phase.

| foldon / symbol | *m/z* (trimer 5+) | A1 | A2 | ΔCV_50_ | dx | R^2^ | m | n | y(0) |
| --- | --- | --- | --- | --- | --- | --- | --- | --- | --- |
| 0 /  | 1848.94 | 97.16 | 0.00 | 13.51 | 2.28 | 0.999 | -10.64 | 192.36 | 48.58 |
| 1 /  | 1875.99 | 94.50 | 0.00 | 11.04 | 2.39 | 0.999 | -9.89 | 156.46 | 47.25 |
| 2 /  | 1875.95 | 98,42 | 0,00 | 11,27 | 2,30 | 0,999 | -10,69 | 169,65 | 49,21 |
| 3 /  | 1841.69 | 96.70 | 0.00 | 11.63 | 2.27 | 0.999 | -10.66 | 172.29 | 48.35 |
| 4 /  | 1841.78 | 98.56 | 0.00 | 12.26 | 2.30 | 0.999 | -10.70 | 180.47 | 49.28 |
| 5 /  | 1862.01 | 97.82 | 0.00 | 12.25 | 2.22 | 0.999 | -10.99 | 183.66 | 48.91 |
| 6 /  | 1877.92 | 99.30 | 0.00 | 13.42 | 2.20 | 0.999 | -11.29 | 201.08 | 49.65 |

**Table S9:** Van der Waals contacts (no heteroatoms) in the foldon 0 trimer.

| chain1 | residue1 | res_id1 | atom1 | chain2 | residue2 | res_id2 | atom2 | distance | intracatenane | intercatenane | total |
| --- | --- | --- | --- | --- | --- | --- | --- | --- | --- | --- | --- |
| A | GLY | 1 | C | A | TYR | 2 | C | 3.39 | yes |  |  |
| A | GLY | 1 | C | A | TYR | 2 | CB | 3.6 | yes |  |  |
| A | GLY | 1 | C | A | TYR | 2 | CG | 3.93 | yes |  |  |
| A | GLY | 1 | C | A | TYR | 2 | CD1 | 3.37 | yes |  |  |
| A | GLY | 1 | C | C | GLU | 5 | CB | 3.86 |  | yes |  |
| A | TYR | 2 | C | A | ILE | 3 | C | 3.28 | yes |  |  |
| A | TYR | 2 | C | A | ILE | 3 | CB | 3.71 | yes |  |  |
| A | TYR | 2 | C | B | TYR | 2 | CD2 | 3.83 |  | yes |  |
| A | TYR | 2 | C | B | TYR | 2 | CE2 | 3.13 |  | yes |  |
| A | TYR | 2 | C | B | TYR | 2 | CZ | 3.93 |  | yes |  |
| A | TYR | 2 | C | C | GLU | 5 | CB | 3.43 |  | yes |  |
| A | TYR | 2 | CB | B | TYR | 2 | CD2 | 3.47 |  | yes |  |
| A | TYR | 2 | CB | B | TYR | 2 | CE2 | 3.37 |  | yes |  |
| A | TYR | 2 | CD1 | C | ILE | 3 | C | 3.77 |  | yes |  |
| A | TYR | 2 | CD1 | C | PRO | 4 | CB | 3.69 |  | yes |  |
| A | TYR | 2 | CD2 | C | TYR | 2 | C | 3.56 |  | yes |  |
| A | TYR | 2 | CD2 | C | TYR | 2 | CB | 3.21 |  | yes |  |
| A | TYR | 2 | CD2 | C | ILE | 3 | C | 3.81 |  | yes |  |
| A | TYR | 2 | CE1 | C | PRO | 4 | CB | 3.79 |  | yes |  |
| A | TYR | 2 | CE2 | C | TYR | 2 | C | 3.33 |  | yes |  |
| A | TYR | 2 | CE2 | C | TYR | 2 | CB | 3.51 |  | yes |  |
| A | ILE | 3 | C | A | PRO | 4 | C | 3.26 | yes |  |  |
| A | ILE | 3 | C | A | PRO | 4 | CB | 3.6 | yes |  |  |
| A | ILE | 3 | C | A | PRO | 4 | CG | 3.59 | yes |  |  |
| A | ILE | 3 | C | A | PRO | 4 | CD | 2.5 | yes |  |  |
| A | ILE | 3 | C | B | TYR | 2 | CG | 3.88 |  | yes |  |
| A | ILE | 3 | C | B | TYR | 2 | CD2 | 3.54 |  | yes |  |
| A | ILE | 3 | C | B | TYR | 2 | CE2 | 3.52 |  | yes |  |
| A | ILE | 3 | C | B | TYR | 2 | CZ | 3.83 |  | yes |  |
| A | ILE | 3 | CB | A | PRO | 4 | CD | 3.39 | yes |  |  |
| A | ILE | 3 | CB | A | ARG | 15 | CZ | 3.58 | yes |  |  |
| A | ILE | 3 | CG1 | A | TRP | 20 | CZ2 | 3.98 | yes |  |  |
| A | ILE | 3 | CG2 | A | ARG | 15 | CG | 3.66 | yes |  |  |
| A | ILE | 3 | CG2 | A | ARG | 15 | CD | 3.83 | yes |  |  |
| A | ILE | 3 | CG2 | A | ARG | 15 | CZ | 3.15 | yes |  |  |
| A | ILE | 3 | CG2 | C | GLU | 5 | C | 3.95 |  | yes |  |
| A | ILE | 3 | CG2 | C | GLU | 5 | CB | 3.36 |  | yes |  |
| A | ILE | 3 | CG2 | C | GLU | 5 | CG | 3.4 |  | yes |  |
| A | ILE | 3 | CG2 | C | GLU | 5 | CD | 3.09 |  | yes |  |
| A | ILE | 3 | CD1 | A | ARG | 15 | CG | 3.29 | yes |  |  |
| A | ILE | 3 | CD1 | A | TRP | 20 | CZ2 | 3.15 | yes |  |  |
| A | ILE | 3 | CD1 | A | TRP | 20 | CH2 | 3.25 | yes |  |  |
| A | ILE | 3 | CD1 | B | ILE | 3 | CD1 | 3.85 |  | yes |  |
| A | ILE | 3 | CD1 | C | ILE | 3 | CD1 | 3.71 |  | yes |  |
| A | ILE | 3 | CD1 | C | TRP | 20 | CZ2 | 2.99 |  | yes |  |
| A | ILE | 3 | CD1 | C | TRP | 20 | CH2 | 3.33 |  | yes |  |
| A | PRO | 4 | C | A | GLU | 5 | C | 3.1 | yes |  |  |
| A | PRO | 4 | C | A | GLU | 5 | CB | 3.72 | yes |  |  |
| A | PRO | 4 | CB | B | TYR | 2 | CD1 | 3.68 |  | yes |  |
| A | PRO | 4 | CB | B | TYR | 2 | CE1 | 3.54 |  | yes |  |
| A | PRO | 4 | CD | A | ARG | 15 | CZ | 3.61 | yes |  |  |
| A | GLU | 5 | C | A | ALA | 6 | C | 3.27 | yes |  |  |
| A | GLU | 5 | C | A | ALA | 6 | CB | 3.68 | yes |  |  |
| A | GLU | 5 | C | A | TRP | 20 | CD1 | 3.55 | yes |  |  |
| A | GLU | 5 | CB | B | GLY | 1 | C | 3.69 |  | yes |  |
| A | GLU | 5 | CB | B | TYR | 2 | C | 3.72 |  | yes |  |
| A | GLU | 5 | CB | B | ILE | 3 | CG2 | 3.53 |  | yes |  |
| A | GLU | 5 | CG | B | ILE | 3 | CG2 | 3.64 |  | yes |  |
| A | GLU | 5 | CD | B | ILE | 3 | CG2 | 2.98 |  | yes |  |
| A | ALA | 6 | C | A | PRO | 7 | C | 3.05 | yes |  |  |
| A | ALA | 6 | C | A | PRO | 7 | CB | 3.66 | yes |  |  |
| A | ALA | 6 | C | A | PRO | 7 | CG | 3.64 | yes |  |  |
| A | ALA | 6 | C | A | PRO | 7 | CD | 2.5 | yes |  |  |
| A | ALA | 6 | CB | A | PRO | 7 | CD | 3.26 | yes |  |  |
| A | ALA | 6 | CB | A | TYR | 13 | CG | 3.96 | yes |  |  |
| A | ALA | 6 | CB | A | TYR | 13 | CD2 | 3.49 | yes |  |  |
| A | ALA | 6 | CB | A | TRP | 20 | CB | 3.51 | yes |  |  |
| A | ALA | 6 | CB | A | TRP | 20 | CG | 3 | yes |  |  |
| A | ALA | 6 | CB | A | TRP | 20 | CD1 | 2.99 | yes |  |  |
| A | ALA | 6 | CB | A | TRP | 20 | CD2 | 3.46 | yes |  |  |
| A | ALA | 6 | CB | A | TRP | 20 | CE2 | 3.71 | yes |  |  |
| A | ALA | 6 | CB | B | ARG | 15 | CZ | 3.54 |  | yes |  |
| A | PRO | 7 | C | A | ARG | 8 | C | 3.45 | yes |  |  |
| A | PRO | 7 | C | A | ARG | 8 | CB | 3.54 | yes |  |  |
| A | PRO | 7 | C | A | ARG | 8 | CZ | 3.98 | yes |  |  |
| A | PRO | 7 | CB | A | TYR | 13 | CE2 | 3.67 | yes |  |  |
| A | PRO | 7 | CG | A | TYR | 13 | CE2 | 3.45 | yes |  |  |
| A | PRO | 7 | CG | A | TYR | 13 | CZ | 3.51 | yes |  |  |
| A | PRO | 7 | CD | A | TYR | 13 | CD2 | 3.67 | yes |  |  |
| A | PRO | 7 | CD | A | TYR | 13 | CE2 | 3.24 | yes |  |  |
| A | PRO | 7 | CD | A | TYR | 13 | CZ | 3.67 | yes |  |  |
| A | ARG | 8 | C | A | ASP | 9 | C | 3.36 | yes |  |  |
| A | ARG | 8 | C | A | ASP | 9 | CB | 3.57 | yes |  |  |
| A | ARG | 8 | CG | B | ASP | 17 | C | 3.56 |  | yes |  |
| A | ASP | 9 | C | A | GLY | 10 | C | 3.18 | yes |  |  |
| A | ASP | 9 | CG | A | GLN | 11 | CB | 3.92 | yes |  |  |
| A | GLY | 10 | C | A | GLN | 11 | C | 3.38 | yes |  |  |
| A | GLY | 10 | C | A | GLN | 11 | CB | 3.56 | yes |  |  |
| A | GLY | 10 | C | B | LYS | 16 | CG | 3.72 |  | yes |  |
| A | GLY | 10 | C | B | LYS | 16 | CD | 3.27 |  | yes |  |
| A | GLY | 10 | C | B | LYS | 16 | CE | 3.57 |  | yes |  |
| A | GLN | 11 | C | A | ALA | 12 | C | 3.33 | yes |  |  |
| A | GLN | 11 | C | A | ALA | 12 | CB | 3.57 | yes |  |  |
| A | GLN | 11 | C | B | LYS | 16 | CB | 3.41 |  | yes |  |
| A | GLN | 11 | C | B | LYS | 16 | CG | 3.42 |  | yes |  |
| A | GLN | 11 | C | B | LYS | 16 | CD | 3.08 |  | yes |  |
| A | GLN | 11 | CB | A | LEU | 22 | CD2 | 3.3 | yes |  |  |
| A | GLN | 11 | CG | A | LEU | 22 | CD2 | 3.37 | yes |  |  |
| A | GLN | 11 | CD | A | LEU | 22 | CD1 | 3.69 | yes |  |  |
| A | GLN | 11 | CD | A | LEU | 22 | CD2 | 3.62 | yes |  |  |
| A | ALA | 12 | C | A | TYR | 13 | C | 3.49 | yes |  |  |
| A | ALA | 12 | C | A | TYR | 13 | CB | 3.44 | yes |  |  |
| A | ALA | 12 | C | A | TYR | 13 | CG | 3.58 | yes |  |  |
| A | ALA | 12 | C | A | TYR | 13 | CD2 | 3.88 | yes |  |  |
| A | ALA | 12 | C | B | VAL | 14 | CG2 | 3.43 |  | yes |  |
| A | ALA | 12 | CB | A | LEU | 23 | CB | 3.85 | yes |  |  |
| A | ALA | 12 | CB | B | VAL | 14 | CG1 | 3.93 |  | yes |  |
| A | ALA | 12 | CB | B | VAL | 14 | CG2 | 3.01 |  | yes |  |
| A | ALA | 12 | CB | B | LYS | 16 | CB | 3.7 |  | yes |  |
| A | ALA | 12 | CB | B | LYS | 16 | CD | 3.94 |  | yes |  |
| A | ALA | 12 | CB | B | PHE | 26 | CE1 | 3.44 |  | yes |  |
| A | ALA | 12 | CB | B | PHE | 26 | CE2 | 3.4 |  | yes |  |
| A | ALA | 12 | CB | B | PHE | 26 | CZ | 2.97 |  | yes |  |
| A | TYR | 13 | C | A | VAL | 14 | C | 3.32 | yes |  |  |
| A | TYR | 13 | C | A | VAL | 14 | CB | 3.5 | yes |  |  |
| A | TYR | 13 | C | A | VAL | 14 | CG2 | 3.35 | yes |  |  |
| A | TYR | 13 | C | A | TRP | 20 | CE3 | 3.8 | yes |  |  |
| A | TYR | 13 | C | B | VAL | 14 | CG2 | 3.39 |  | yes |  |
| A | TYR | 13 | CB | A | TRP | 20 | C | 3.97 | yes |  |  |
| A | TYR | 13 | CB | A | TRP | 20 | CB | 3.38 | yes |  |  |
| A | TYR | 13 | CB | A | TRP | 20 | CG | 3.93 | yes |  |  |
| A | TYR | 13 | CB | A | TRP | 20 | CD2 | 3.9 | yes |  |  |
| A | TYR | 13 | CB | A | TRP | 20 | CE3 | 3.45 | yes |  |  |
| A | TYR | 13 | CG | A | TRP | 20 | C | 3.84 | yes |  |  |
| A | TYR | 13 | CG | A | TRP | 20 | CB | 3.34 | yes |  |  |
| A | TYR | 13 | CD1 | A | TRP | 20 | C | 3.12 | yes |  |  |
| A | TYR | 13 | CD1 | A | TRP | 20 | CB | 3.47 | yes |  |  |
| A | TYR | 13 | CD1 | A | VAL | 21 | C | 3.26 | yes |  |  |
| A | TYR | 13 | CD2 | A | TRP | 20 | CB | 4 | yes |  |  |
| A | TYR | 13 | CE1 | A | TRP | 20 | C | 3.82 | yes |  |  |
| A | TYR | 13 | CE1 | A | VAL | 21 | C | 3.97 | yes |  |  |
| A | TYR | 13 | CE1 | A | LEU | 22 | CG | 3.95 | yes |  |  |
| A | TYR | 13 | CE1 | A | LEU | 22 | CD2 | 3.91 | yes |  |  |
| A | VAL | 14 | C | A | ARG | 15 | C | 3.33 | yes |  |  |
| A | VAL | 14 | C | A | ARG | 15 | CB | 3.52 | yes |  |  |
| A | VAL | 14 | C | A | TRP | 20 | CE3 | 3.43 | yes |  |  |
| A | VAL | 14 | C | A | TRP | 20 | CZ3 | 3.51 | yes |  |  |
| A | VAL | 14 | CB | A | VAL | 21 | CG2 | 3.25 | yes |  |  |
| A | VAL | 14 | CB | C | ALA | 12 | CB | 3.84 |  | yes |  |
| A | VAL | 14 | CB | C | LEU | 23 | CD2 | 3.88 |  | yes |  |
| A | VAL | 14 | CG1 | C | ALA | 12 | C | 3.97 |  | yes |  |
| A | VAL | 14 | CG1 | C | ALA | 12 | CB | 3.42 |  | yes |  |
| A | VAL | 14 | CG1 | C | TYR | 13 | C | 3.77 |  | yes |  |
| A | VAL | 14 | CG1 | C | LEU | 23 | CB | 3.46 |  | yes |  |
| A | VAL | 14 | CG1 | C | LEU | 23 | CG | 3.86 |  | yes |  |
| A | VAL | 14 | CG1 | C | LEU | 23 | CD2 | 3.17 |  | yes |  |
| A | VAL | 14 | CG2 | A | VAL | 21 | C | 3.85 | yes |  |  |
| A | VAL | 14 | CG2 | A | VAL | 21 | CG2 | 3.3 | yes |  |  |
| A | VAL | 14 | CG2 | A | LEU | 22 | C | 3.93 | yes |  |  |
| A | VAL | 14 | CG2 | A | LEU | 23 | CB | 3.81 | yes |  |  |
| A | VAL | 14 | CG2 | C | LEU | 23 | CD2 | 3.54 |  | yes |  |
| A | ARG | 15 | C | A | LYS | 16 | C | 3.61 | yes |  |  |
| A | ARG | 15 | C | A | LYS | 16 | CB | 3.35 | yes |  |  |
| A | ARG | 15 | CB | A | TRP | 20 | CZ3 | 3.37 | yes |  |  |
| A | ARG | 15 | CB | A | TRP | 20 | CH2 | 3.66 | yes |  |  |
| A | ARG | 15 | CB | C | TYR | 13 | O | 3.84 |  | yes |  |
| A | ARG | 15 | CB | C | TRP | 20 | CZ3 | 3.69 |  | yes |  |
| A | ARG | 15 | CB | C | TRP | 20 | CH2 | 3.91 |  | yes |  |
| A | ARG | 15 | CG | A | TRP | 20 | CE3 | 3.91 | yes |  |  |
| A | ARG | 15 | CG | A | TRP | 20 | CZ2 | 3.64 | yes |  |  |
| A | ARG | 15 | CG | A | TRP | 20 | CZ3 | 3.31 | yes |  |  |
| A | ARG | 15 | CG | A | TRP | 20 | CH2 | 3.15 | yes |  |  |
| A | ARG | 15 | CD | A | GLU | 19 | C | 3.73 | yes |  |  |
| A | ARG | 15 | CD | A | TRP | 20 | CD2 | 3.94 | yes |  |  |
| A | ARG | 15 | CD | A | TRP | 20 | CE2 | 3.98 | yes |  |  |
| A | ARG | 15 | CD | A | TRP | 20 | CE3 | 3.84 | yes |  |  |
| A | ARG | 15 | CD | A | TRP | 20 | CZ2 | 3.95 | yes |  |  |
| A | ARG | 15 | CD | A | TRP | 20 | CZ3 | 3.78 | yes |  |  |
| A | ARG | 15 | CD | A | TRP | 20 | CH2 | 3.84 | yes |  |  |
| A | ARG | 15 | CZ | A | GLY | 18 | C | 2.91 | yes |  |  |
| A | ARG | 15 | CZ | C | GLU | 5 | OE2 | 2.98 |  | yes |  |
| A | LYS | 16 | C | A | ASP | 17 | C | 2.94 | yes |  |  |
| A | LYS | 16 | C | A | ASP | 17 | CB | 3.18 | yes |  |  |
| A | LYS | 16 | C | A | ASP | 17 | CG | 2.98 | yes |  |  |
| A | LYS | 16 | C | A | ASP | 17 | OD1 | 2.91 | yes |  |  |
| A | LYS | 16 | C | A | ASP | 17 | OD2 | 3.66 | yes |  |  |
| A | LYS | 16 | CB | A | ASP | 17 | CG | 3.99 | yes |  |  |
| A | LYS | 16 | CB | A | ASP | 17 | OD2 | 3.93 | yes |  |  |
| A | LYS | 16 | CB | C | GLN | 11 | C | 3.24 |  | yes |  |
| A | LYS | 16 | CB | C | ALA | 12 | CB | 3.65 |  | yes |  |
| A | LYS | 16 | CG | A | ASP | 17 | CB | 3.94 | yes |  |  |
| A | LYS | 16 | CG | A | ASP | 17 | CG | 2.83 | yes |  |  |
| A | LYS | 16 | CG | A | VAL | 21 | CG1 | 3.92 | yes |  |  |
| A | LYS | 16 | CG | C | GLY | 10 | C | 3.83 |  | yes |  |
| A | LYS | 16 | CG | C | GLN | 11 | C | 3.96 |  | yes |  |
| A | LYS | 16 | CD | A | ASP | 17 | CG | 3.72 | yes |  |  |
| A | LYS | 16 | CD | A | ASP | 17 | OD1 | 3.7 | yes |  |  |
| A | LYS | 16 | CD | A | ASP | 17 | OD2 | 3.14 | yes |  |  |
| A | LYS | 16 | CD | A | VAL | 21 | CG1 | 3.41 | yes |  |  |
| A | LYS | 16 | CE | A | ASP | 17 | OD2 | 3.51 | yes |  |  |
| A | LYS | 16 | CE | C | GLY | 10 | C | 3.69 |  | yes |  |
| A | LYS | 16 | NZ | A | PHE | 26 | CE2 | 3.73 | yes |  |  |
| A | LYS | 16 | NZ | A | PHE | 26 | CZ | 3.25 | yes |  |  |
| A | ASP | 17 | C | A | GLY | 18 | C | 3.05 | yes |  |  |
| A | ASP | 17 | C | C | ARG | 8 | CG | 3.46 |  | yes |  |
| A | GLY | 18 | C | A | GLU | 19 | C | 3.56 | yes |  |  |
| A | GLY | 18 | C | A | GLU | 19 | CB | 3.34 | yes |  |  |
| A | GLY | 18 | C | A | GLU | 19 | CG | 3.48 | yes |  |  |
| A | GLU | 19 | C | A | TRP | 20 | C | 3.24 | yes |  |  |
| A | GLU | 19 | C | A | TRP | 20 | CB | 3.61 | yes |  |  |
| A | GLU | 19 | C | A | TRP | 20 | CG | 3.9 | yes |  |  |
| A | TRP | 20 | C | A | VAL | 21 | C | 3.38 | yes |  |  |
| A | TRP | 20 | C | A | VAL | 21 | CB | 3.57 | yes |  |  |
| A | TRP | 20 | C | A | VAL | 21 | CG1 | 3.55 | yes |  |  |
| A | TRP | 20 | CE3 | B | ARG | 15 | CB | 3.95 |  | yes |  |
| A | TRP | 20 | CZ2 | B | ILE | 3 | CD1 | 3.11 |  | yes |  |
| A | TRP | 20 | CZ2 | B | ARG | 15 | CG | 3.96 |  | yes |  |
| A | TRP | 20 | CZ3 | B | ARG | 15 | CB | 3.64 |  | yes |  |
| A | TRP | 20 | CH2 | B | ILE | 3 | CD1 | 3.63 |  | yes |  |
| A | TRP | 20 | CH2 | B | ARG | 15 | CB | 3.79 |  | yes |  |
| A | TRP | 20 | CH2 | B | ARG | 15 | CG | 3.83 |  | yes |  |
| A | TRP | 20 | CH2 | C | TRP | 20 | CH2 | 3.87 |  | yes |  |
| A | VAL | 21 | C | A | LEU | 22 | C | 2.89 | yes |  |  |
| A | VAL | 21 | C | A | LEU | 22 | CB | 3.64 | yes |  |  |
| A | VAL | 21 | CB | A | PHE | 26 | CE1 | 3.89 | yes |  |  |
| A | VAL | 21 | CG2 | A | PHE | 26 | CE1 | 2.99 | yes |  |  |
| A | VAL | 21 | CG2 | A | PHE | 26 | CZ | 3.39 | yes |  |  |
| A | LEU | 22 | C | A | LEU | 23 | C | 2.96 | yes |  |  |
| A | LEU | 22 | C | A | LEU | 23 | CB | 3.68 | yes |  |  |
| A | LEU | 23 | C | A | SER | 24 | C | 2.92 | yes |  |  |
| A | LEU | 23 | C | A | SER | 24 | CB | 3.71 | yes |  |  |
| A | LEU | 23 | CG | B | PHE | 26 | CB | 3.54 |  | yes |  |
| A | LEU | 23 | CG | B | PHE | 26 | CG | 3.6 |  | yes |  |
| A | LEU | 23 | CG | B | PHE | 26 | CD2 | 3.97 |  | yes |  |
| A | LEU | 23 | CD1 | B | LEU | 23 | CD1 | 3.67 |  | yes |  |
| A | LEU | 23 | CD1 | C | LEU | 23 | CD1 | 3.51 |  | yes |  |
| A | LEU | 23 | CD1 | C | LEU | 23 | CD2 | 3.31 |  | yes |  |
| A | LEU | 23 | CD2 | B | VAL | 14 | CB | 3.52 |  | yes |  |
| A | LEU | 23 | CD2 | B | VAL | 14 | CG1 | 3.46 |  | yes |  |
| A | LEU | 23 | CD2 | B | VAL | 14 | CG2 | 3.61 |  | yes |  |
| A | LEU | 23 | CD2 | B | LEU | 23 | CD1 | 3.4 |  | yes |  |
| A | LEU | 23 | CD2 | B | PHE | 26 | CB | 3.25 |  | yes |  |
| A | LEU | 23 | CD2 | B | PHE | 26 | CG | 3.09 |  | yes |  |
| A | LEU | 23 | CD2 | B | PHE | 26 | CD1 | 3.14 |  | yes |  |
| A | LEU | 23 | CD2 | B | PHE | 26 | CD2 | 3.84 |  | yes |  |
| A | LEU | 23 | CD2 | B | PHE | 26 | CE1 | 3.93 |  | yes |  |
| A | SER | 24 | C | A | THR | 25 | C | 3.02 | yes |  |  |
| A | SER | 24 | C | A | THR | 25 | CB | 3.71 | yes |  |  |
| A | THR | 25 | C | A | PHE | 26 | C | 3.32 | yes |  |  |
| A | THR | 25 | C | A | PHE | 26 | CB | 3.62 | yes |  |  |
| A | THR | 25 | C | A | PHE | 26 | CG | 3.9 | yes |  |  |
| A | THR | 25 | C | A | PHE | 26 | CD1 | 3.67 | yes |  |  |
| A | PHE | 26 | C | A | LEU | 27 | C | 3.44 | yes |  |  |
| A | PHE | 26 | C | A | LEU | 27 | CB | 3.47 | yes |  |  |
| A | PHE | 26 | C | A | LEU | 27 | CD1 | 3.83 | yes |  |  |
| A | PHE | 26 | C | C | LEU | 27 | CD2 | 3.39 |  | yes |  |
| A | PHE | 26 | CB | C | LEU | 23 | CG | 3.86 |  | yes |  |
| A | PHE | 26 | CB | C | LEU | 23 | CD2 | 3.22 |  | yes |  |
| A | PHE | 26 | CB | C | LEU | 27 | CD2 | 3.62 |  | yes |  |
| A | PHE | 26 | CG | C | LEU | 23 | CG | 3.82 |  | yes |  |
| A | PHE | 26 | CG | C | LEU | 23 | CD2 | 2.91 |  | yes |  |
| A | PHE | 26 | CD1 | C | LEU | 23 | CD2 | 3.4 |  | yes |  |
| A | PHE | 26 | CD2 | C | LEU | 23 | CG | 3.64 |  | yes |  |
| A | PHE | 26 | CD2 | C | LEU | 23 | CD2 | 3.11 |  | yes |  |
| A | PHE | 26 | CE1 | C | LEU | 23 | CD2 | 4 |  | yes |  |
| A | PHE | 26 | CE2 | C | ALA | 12 | CB | 3.38 |  | yes |  |
| A | PHE | 26 | CE2 | C | LEU | 23 | CD2 | 3.75 |  | yes |  |
| A | PHE | 26 | CZ | C | ALA | 12 | CB | 3.34 |  | yes |  |
| A | LEU | 27 | CD1 | B | LEU | 27 | CD1 | 3.32 |  | yes |  |
| A | LEU | 27 | CD1 | C | LEU | 27 | CD1 | 3.34 |  | yes |  |
| A | LEU | 27 | CD2 | B | PHE | 26 | C | 3.11 |  | yes |  |
| A | LEU | 27 | CD2 | B | PHE | 26 | CB | 3.82 |  | yes |  |
| B | GLY | 1 | C | B | TYR | 2 | C | 3.58 | yes |  |  |
| B | GLY | 1 | C | B | TYR | 2 | CB | 3.39 | yes |  |  |
| B | GLY | 1 | C | B | TYR | 2 | CG | 3.74 | yes |  |  |
| B | GLY | 1 | C | B | TYR | 2 | CD1 | 3.25 | yes |  |  |
| B | TYR | 2 | C | B | ILE | 3 | C | 3.28 | yes |  |  |
| B | TYR | 2 | C | B | ILE | 3 | CB | 3.7 | yes |  |  |
| B | TYR | 2 | C | C | TYR | 2 | CD2 | 3.65 |  | yes |  |
| B | TYR | 2 | C | C | TYR | 2 | CE2 | 3.38 |  | yes |  |
| B | TYR | 2 | CB | C | TYR | 2 | CD2 | 3.24 |  | yes |  |
| B | TYR | 2 | CB | C | TYR | 2 | CE2 | 3.25 |  | yes |  |
| B | ILE | 3 | C | B | PRO | 4 | C | 3.25 | yes |  |  |
| B | ILE | 3 | C | B | PRO | 4 | CB | 3.57 | yes |  |  |
| B | ILE | 3 | C | B | PRO | 4 | CG | 3.65 | yes |  |  |
| B | ILE | 3 | C | B | PRO | 4 | CD | 2.51 | yes |  |  |
| B | ILE | 3 | C | C | TYR | 2 | CB | 3.93 | yes |  |  |
| B | ILE | 3 | C | C | TYR | 2 | CG | 3.5 |  | yes |  |
| B | ILE | 3 | C | C | TYR | 2 | CD1 | 3.67 |  | yes |  |
| B | ILE | 3 | C | C | TYR | 2 | CD2 | 3.75 |  | yes |  |
| B | ILE | 3 | CB | B | PRO | 4 | CD | 3.2 | yes |  |  |
| B | ILE | 3 | CG2 | B | ARG | 15 | CD | 3.8 | yes |  |  |
| B | ILE | 3 | CG2 | B | ARG | 15 | CZ | 3.98 | yes |  |  |
| B | ILE | 3 | CD1 | B | ARG | 15 | CG | 3.28 | yes |  |  |
| B | ILE | 3 | CD1 | B | ARG | 15 | CD | 3.99 | yes |  |  |
| B | ILE | 3 | CD1 | B | TRP | 20 | CZ2 | 3.34 | yes |  |  |
| B | ILE | 3 | CD1 | B | TRP | 20 | CH2 | 3.43 | yes |  |  |
| B | ILE | 3 | CD1 | C | ILE | 3 | CD1 | 3.77 |  | yes |  |
| B | PRO | 4 | C | B | GLU | 5 | C | 3.03 | yes |  |  |
| B | PRO | 4 | C | B | GLU | 5 | CB | 3.71 | yes |  |  |
| B | PRO | 4 | CB | C | TYR | 2 | CD1 | 3.75 |  | yes |  |
| B | PRO | 4 | CB | C | TYR | 2 | CE1 | 3.91 |  | yes |  |
| B | GLU | 5 | C | B | ALA | 6 | C | 3.31 | yes |  |  |
| B | GLU | 5 | C | B | ALA | 6 | CB | 3.65 | yes |  |  |
| B | GLU | 5 | C | B | TRP | 20 | CD1 | 3.83 | yes |  |  |
| B | GLU | 5 | C | C | ILE | 3 | CG2 | 3.94 |  | yes |  |
| B | GLU | 5 | CB | C | TYR | 2 | C | 3.62 |  | yes |  |
| B | GLU | 5 | CB | C | ILE | 3 | CG2 | 3.32 |  | yes |  |
| B | GLU | 5 | CG | C | ILE | 3 | CG2 | 3.4 |  | yes |  |
| B | GLU | 5 | CD | B | ARG | 8 | CZ | 3.65 | yes |  |  |
| B | GLU | 5 | CD | C | ILE | 3 | CG2 | 3.07 |  | yes |  |
| B | ALA | 6 | C | B | PRO | 7 | C | 3 | yes |  |  |
| B | ALA | 6 | C | B | PRO | 7 | CB | 3.67 | yes |  |  |
| B | ALA | 6 | C | B | PRO | 7 | CG | 3.64 | yes |  |  |
| B | ALA | 6 | C | B | PRO | 7 | CD | 2.49 | yes |  |  |
| B | ALA | 6 | CB | B | PRO | 7 | CD | 3.37 | yes |  |  |
| B | ALA | 6 | CB | B | TYR | 13 | CD2 | 3.87 | yes |  |  |
| B | ALA | 6 | CB | B | TRP | 20 | CB | 3.78 | yes |  |  |
| B | ALA | 6 | CB | B | TRP | 20 | CG | 3.11 | yes |  |  |
| B | ALA | 6 | CB | B | TRP | 20 | CD1 | 3.09 | yes |  |  |
| B | ALA | 6 | CB | B | TRP | 20 | CD2 | 3.35 | yes |  |  |
| B | ALA | 6 | CB | B | TRP | 20 | NE1 | 3.32 | yes |  |  |
| B | ALA | 6 | CB | B | TRP | 20 | CE2 | 3.47 | yes |  |  |
| B | PRO | 7 | C | B | ARG | 8 | C | 3.45 | yes |  |  |
| B | PRO | 7 | C | B | ARG | 8 | CB | 3.54 | yes |  |  |
| B | PRO | 7 | C | B | ARG | 8 | CG | 3.96 | yes |  |  |
| B | PRO | 7 | C | B | ARG | 8 | CD | 3.71 | yes |  |  |
| B | PRO | 7 | C | B | ASP | 9 | OD1 | 3.95 | yes |  |  |
| B | PRO | 7 | CB | B | ASP | 9 | OD1 | 3.95 | yes |  |  |
| B | PRO | 7 | CB | B | TYR | 13 | CE2 | 3.87 | yes |  |  |
| B | PRO | 7 | CG | B | TYR | 13 | CE2 | 3.23 | yes |  |  |
| B | PRO | 7 | CG | B | TYR | 13 | CZ | 3.47 | yes |  |  |
| B | PRO | 7 | CD | B | TYR | 13 | CE2 | 3.78 | yes |  |  |
| B | ARG | 8 | C | B | ASP | 9 | C | 3.43 | yes |  |  |
| B | ARG | 8 | C | B | ASP | 9 | CB | 3.54 | yes |  |  |
| B | ARG | 8 | CG | C | ASP | 17 | C | 3.53 |  | yes |  |
| B | ASP | 9 | C | B | GLY | 10 | C | 3.16 | yes |  |  |
| B | ASP | 9 | CG | B | GLN | 11 | CB | 3.61 | yes |  |  |
| B | GLY | 10 | C | B | GLN | 11 | C | 3.3 | yes |  |  |
| B | GLY | 10 | C | B | GLN | 11 | CB | 3.59 | yes |  |  |
| B | GLY | 10 | C | C | LYS | 16 | CG | 3.64 |  | yes |  |
| B | GLY | 10 | C | C | LYS | 16 | CE | 3.58 |  | yes |  |
| B | GLN | 11 | C | B | ALA | 12 | C | 3.26 | yes |  |  |
| B | GLN | 11 | C | B | ALA | 12 | CB | 3.61 | yes |  |  |
| B | GLN | 11 | C | C | LYS | 16 | CB | 3.31 |  | yes |  |
| B | GLN | 11 | C | C | LYS | 16 | CG | 3.57 |  | yes |  |
| B | GLN | 11 | CB | B | LEU | 22 | CD2 | 3.28 | yes |  |  |
| B | GLN | 11 | CG | B | LEU | 22 | CD1 | 3.79 | yes |  |  |
| B | GLN | 11 | CG | B | LEU | 22 | CD2 | 3.47 | yes |  |  |
| B | GLN | 11 | CD | B | LEU | 22 | CD1 | 3.66 | yes |  |  |
| B | GLN | 11 | CD | B | LEU | 22 | CD2 | 3.35 | yes |  |  |
| B | ALA | 12 | C | B | TYR | 13 | C | 3.45 | yes |  |  |
| B | ALA | 12 | C | B | TYR | 13 | CB | 3.46 | yes |  |  |
| B | ALA | 12 | C | B | TYR | 13 | CG | 3.67 | yes |  |  |
| B | ALA | 12 | C | B | TYR | 13 | CD2 | 3.97 | yes |  |  |
| B | ALA | 12 | C | B | LEU | 22 | CD2 | 3.62 | yes |  |  |
| B | ALA | 12 | C | C | VAL | 14 | CG1 | 3.92 |  | yes |  |
| B | ALA | 12 | CB | B | LEU | 23 | CB | 3.75 | yes |  |  |
| B | ALA | 12 | CB | C | VAL | 14 | CB | 3.96 |  | yes |  |
| B | ALA | 12 | CB | C | VAL | 14 | CG1 | 3.43 |  | yes |  |
| B | ALA | 12 | CB | C | LYS | 16 | CB | 3.91 |  | yes |  |
| B | ALA | 12 | CB | C | PHE | 26 | CE1 | 3.84 |  | yes |  |
| B | ALA | 12 | CB | C | PHE | 26 | CE2 | 3.24 |  | yes |  |
| B | ALA | 12 | CB | C | PHE | 26 | CZ | 3.02 |  | yes |  |
| B | TYR | 13 | C | B | VAL | 14 | C | 3.31 | yes |  |  |
| B | TYR | 13 | C | B | VAL | 14 | CB | 3.46 | yes |  |  |
| B | TYR | 13 | C | B | VAL | 14 | CG1 | 3.91 | yes |  |  |
| B | TYR | 13 | C | B | TRP | 20 | CE3 | 3.99 | yes |  |  |
| B | TYR | 13 | C | C | VAL | 14 | CG1 | 3.59 |  | yes |  |
| B | TYR | 13 | CB | B | TRP | 20 | CB | 3.5 | yes |  |  |
| B | TYR | 13 | CB | B | TRP | 20 | CE3 | 3.52 | yes |  |  |
| B | TYR | 13 | CG | B | TRP | 20 | C | 3.89 | yes |  |  |
| B | TYR | 13 | CG | B | TRP | 20 | CB | 3.37 | yes |  |  |
| B | TYR | 13 | CD1 | B | TRP | 20 | C | 3.13 | yes |  |  |
| B | TYR | 13 | CD1 | B | TRP | 20 | CB | 3.44 | yes |  |  |
| B | TYR | 13 | CD1 | B | VAL | 21 | C | 3.18 | yes |  |  |
| B | TYR | 13 | CE1 | B | TRP | 20 | C | 3.77 | yes |  |  |
| B | TYR | 13 | CE1 | B | VAL | 21 | C | 3.91 | yes |  |  |
| B | VAL | 14 | C | B | ARG | 15 | C | 3.28 | yes |  |  |
| B | VAL | 14 | C | B | ARG | 15 | CB | 3.52 | yes |  |  |
| B | VAL | 14 | C | B | TRP | 20 | CE3 | 3.46 | yes |  |  |
| B | VAL | 14 | C | B | TRP | 20 | CZ3 | 3.59 | yes |  |  |
| B | VAL | 14 | CG1 | B | VAL | 21 | C | 3.66 | yes |  |  |
| B | VAL | 14 | CG1 | B | VAL | 21 | CB | 3.86 | yes |  |  |
| B | VAL | 14 | CG1 | B | PHE | 26 | CD1 | 3.32 | yes |  |  |
| B | VAL | 14 | CG1 | B | PHE | 26 | CE1 | 3.15 | yes |  |  |
| B | VAL | 14 | CG2 | B | ARG | 15 | C | 3.99 | yes |  |  |
| B | ARG | 15 | C | B | LYS | 16 | C | 3.61 | yes |  |  |
| B | ARG | 15 | C | B | LYS | 16 | CB | 3.34 | yes |  |  |
| B | ARG | 15 | CB | B | TRP | 20 | CZ3 | 3.36 | yes |  |  |
| B | ARG | 15 | CB | B | TRP | 20 | CH2 | 3.69 | yes |  |  |
| B | ARG | 15 | CG | B | TRP | 20 | CE3 | 3.88 | yes |  |  |
| B | ARG | 15 | CG | B | TRP | 20 | CZ2 | 3.6 | yes |  |  |
| B | ARG | 15 | CG | B | TRP | 20 | CZ3 | 3.19 | yes |  |  |
| B | ARG | 15 | CG | B | TRP | 20 | CH2 | 3.03 | yes |  |  |
| B | ARG | 15 | CD | B | TRP | 20 | CH2 | 3.93 | yes |  |  |
| B | LYS | 16 | C | B | ASP | 17 | C | 2.95 | yes |  |  |
| B | LYS | 16 | C | B | ASP | 17 | CB | 3.15 | yes |  |  |
| B | LYS | 16 | C | B | ASP | 17 | CG | 2.9 | yes |  |  |
| B | LYS | 16 | CB | B | ASP | 17 | CG | 3.9 | yes |  |  |
| B | LYS | 16 | CB | B | VAL | 21 | CG2 | 3.91 | yes |  |  |
| B | LYS | 16 | CG | B | ASP | 17 | CG | 2.92 | yes |  |  |
| B | ASP | 17 | C | B | GLY | 18 | C | 3.07 | yes |  |  |
| B | GLY | 18 | C | B | GLU | 19 | C | 3.57 | yes |  |  |
| B | GLY | 18 | C | B | GLU | 19 | CB | 3.35 | yes |  |  |
| B | GLY | 18 | C | B | GLU | 19 | CG | 3.33 | yes |  |  |
| B | GLU | 19 | C | B | TRP | 20 | C | 3.26 | yes |  |  |
| B | GLU | 19 | C | B | TRP | 20 | CB | 3.61 | yes |  |  |
| B | GLU | 19 | C | B | TRP | 20 | CG | 3.84 | yes |  |  |
| B | TRP | 20 | C | B | VAL | 21 | C | 3.41 | yes |  |  |
| B | TRP | 20 | C | B | VAL | 21 | CB | 3.57 | yes |  |  |
| B | TRP | 20 | C | B | VAL | 21 | CG2 | 3.69 | yes |  |  |
| B | TRP | 20 | CZ2 | C | ILE | 3 | CD1 | 3 |  | yes |  |
| B | TRP | 20 | CZ3 | C | ARG | 15 | CB | 3.68 |  | yes |  |
| B | TRP | 20 | CH2 | C | ILE | 3 | CD1 | 3.34 |  | yes |  |
| B | TRP | 20 | CH2 | C | ARG | 15 | CB | 3.84 |  | yes |  |
| B | TRP | 20 | CH2 | C | TRP | 20 | CH2 | 3.97 |  | yes |  |
| B | VAL | 21 | CB | B | PHE | 26 | CE1 | 3.98 | yes |  |  |
| B | VAL | 21 | CG1 | B | PHE | 26 | CE1 | 3.91 | yes |  |  |
| B | LEU | 22 | C | B | LEU | 23 | C | 2.93 | yes |  |  |
| B | LEU | 22 | C | B | LEU | 23 | CB | 3.7 | yes |  |  |
| B | LEU | 23 | C | B | SER | 24 | C | 2.98 | yes |  |  |
| B | LEU | 23 | C | B | SER | 24 | CB | 3.69 | yes |  |  |
| B | LEU | 23 | CB | C | VAL | 14 | CG1 | 3.53 |  | yes |  |
| B | LEU | 23 | CG | C | VAL | 14 | CG1 | 3.87 |  | yes |  |
| B | LEU | 23 | CG | C | PHE | 26 | CB | 3.65 |  | yes |  |
| B | LEU | 23 | CG | C | PHE | 26 | CG | 3.56 |  | yes |  |
| B | LEU | 23 | CG | C | PHE | 26 | CD2 | 3.64 |  | yes |  |
| B | LEU | 23 | CD1 | C | LEU | 23 | CD1 | 3.53 |  | yes |  |
| B | LEU | 23 | CD2 | C | VAL | 14 | CB | 3.77 |  | yes |  |
| B | LEU | 23 | CD2 | C | VAL | 14 | CG1 | 3.1 |  | yes |  |
| B | LEU | 23 | CD2 | C | VAL | 14 | CG2 | 3.36 |  | yes |  |
| B | LEU | 23 | CD2 | C | LEU | 23 | CD1 | 3.42 |  | yes |  |
| B | LEU | 23 | CD2 | C | PHE | 26 | CB | 3.25 |  | yes |  |
| B | LEU | 23 | CD2 | C | PHE | 26 | CG | 2.92 |  | yes |  |
| B | LEU | 23 | CD2 | C | PHE | 26 | CD1 | 3.09 |  | yes |  |
| B | LEU | 23 | CD2 | C | PHE | 26 | CD2 | 3.41 |  | yes |  |
| B | LEU | 23 | CD2 | C | PHE | 26 | CE1 | 3.71 |  | yes |  |
| B | LEU | 23 | CD2 | C | PHE | 26 | CE2 | 3.97 |  | yes |  |
| B | SER | 24 | C | B | THR | 25 | C | 3.01 | yes |  |  |
| B | SER | 24 | C | B | THR | 25 | CB | 3.72 | yes |  |  |
| B | SER | 24 | C | B | LEU | 27 | CB | 3.88 | yes |  |  |
| B | THR | 25 | C | B | PHE | 26 | C | 3.31 | yes |  |  |
| B | THR | 25 | C | B | PHE | 26 | CB | 3.61 | yes |  |  |
| B | THR | 25 | C | B | PHE | 26 | CG | 3.82 | yes |  |  |
| B | THR | 25 | C | B | PHE | 26 | CD1 | 3.9 | yes |  |  |
| B | PHE | 26 | C | B | LEU | 27 | C | 3.55 | yes |  |  |
| B | PHE | 26 | C | B | LEU | 27 | CB | 3.35 | yes |  |  |
| B | PHE | 26 | C | B | LEU | 27 | CD1 | 3.49 | yes |  |  |
| B | LEU | 27 | CD1 | C | LEU | 27 | CD1 | 3.48 |  | yes |  |
| B | LEU | 27 | CD2 | C | PHE | 26 | C | 3.56 |  | yes |  |
| B | LEU | 27 | CD2 | C | PHE | 26 | CB | 3.64 |  | yes |  |
| C | GLY | 1 | C | C | TYR | 2 | C | 3.38 | yes |  |  |
| C | GLY | 1 | C | C | TYR | 2 | CB | 3.6 | yes |  |  |
| C | GLY | 1 | C | C | TYR | 2 | CG | 3.92 | yes |  |  |
| C | GLY | 1 | C | C | TYR | 2 | CD1 | 3.35 | yes |  |  |
| C | TYR | 2 | C | C | ILE | 3 | C | 3.24 | yes |  |  |
| C | TYR | 2 | C | C | ILE | 3 | CB | 3.73 | yes |  |  |
| C | ILE | 3 | C | C | PRO | 4 | C | 3.21 | yes |  |  |
| C | ILE | 3 | C | C | PRO | 4 | CB | 3.61 | yes |  |  |
| C | ILE | 3 | C | C | PRO | 4 | CG | 3.59 | yes |  |  |
| C | ILE | 3 | C | C | PRO | 4 | CD | 2.5 | yes |  |  |
| C | ILE | 3 | CB | C | PRO | 4 | CD | 3.36 | yes |  |  |
| C | ILE | 3 | CB | C | ARG | 15 | CZ | 3.62 | yes |  |  |
| C | ILE | 3 | CG1 | C | TRP | 20 | CZ2 | 3.98 | yes |  |  |
| C | ILE | 3 | CG2 | C | ARG | 15 | CG | 3.73 | yes |  |  |
| C | ILE | 3 | CG2 | C | ARG | 15 | CD | 3.87 | yes |  |  |
| C | ILE | 3 | CG2 | C | ARG | 15 | CZ | 3.17 | yes |  |  |
| C | ILE | 3 | CD1 | C | ARG | 15 | CG | 3.27 | yes |  |  |
| C | ILE | 3 | CD1 | C | TRP | 20 | CZ2 | 3.19 | yes |  |  |
| C | ILE | 3 | CD1 | C | TRP | 20 | CH2 | 3.33 | yes |  |  |
| C | PRO | 4 | C | C | GLU | 5 | C | 3.07 | yes |  |  |
| C | PRO | 4 | C | C | GLU | 5 | CB | 3.73 | yes |  |  |
| C | PRO | 4 | CD | C | ARG | 15 | CZ | 3.62 | yes |  |  |
| C | GLU | 5 | C | C | ALA | 6 | C | 3.24 | yes |  |  |
| C | GLU | 5 | C | C | ALA | 6 | CB | 3.67 | yes |  |  |
| C | GLU | 5 | C | C | TRP | 20 | CD1 | 3.88 | yes |  |  |
| C | GLU | 5 | CG | C | ALA | 6 | C | 4 | yes |  |  |
| C | ALA | 6 | C | C | PRO | 7 | C | 3.07 | yes |  |  |
| C | ALA | 6 | C | C | PRO | 7 | CB | 3.67 | yes |  |  |
| C | ALA | 6 | C | C | PRO | 7 | CG | 3.64 | yes |  |  |
| C | ALA | 6 | C | C | PRO | 7 | CD | 2.5 | yes |  |  |
| C | ALA | 6 | CB | C | PRO | 7 | CD | 3.14 | yes |  |  |
| C | ALA | 6 | CB | C | TYR | 13 | CD2 | 3.79 | yes |  |  |
| C | ALA | 6 | CB | C | TRP | 20 | CB | 3.88 | yes |  |  |
| C | ALA | 6 | CB | C | TRP | 20 | CG | 3.36 | yes |  |  |
| C | ALA | 6 | CB | C | TRP | 20 | CD1 | 3.45 | yes |  |  |
| C | ALA | 6 | CB | C | TRP | 20 | CD2 | 3.62 | yes |  |  |
| C | ALA | 6 | CB | C | TRP | 20 | CE2 | 3.84 | yes |  |  |
| C | PRO | 7 | C | C | ARG | 8 | C | 3.45 | yes |  |  |
| C | PRO | 7 | C | C | ARG | 8 | CB | 3.53 | yes |  |  |
| C | PRO | 7 | C | C | ARG | 8 | CG | 3.93 | yes |  |  |
| C | PRO | 7 | C | C | ARG | 8 | CD | 3.55 | yes |  |  |
| C | PRO | 7 | CB | C | TYR | 13 | CE2 | 3.76 | yes |  |  |
| C | PRO | 7 | CG | C | TYR | 13 | CE2 | 3.32 | yes |  |  |
| C | PRO | 7 | CG | C | TYR | 13 | CZ | 3.57 | yes |  |  |
| C | PRO | 7 | CD | C | TYR | 13 | CD2 | 3.47 | yes |  |  |
| C | PRO | 7 | CD | C | TYR | 13 | CE2 | 3.28 | yes |  |  |
| C | PRO | 7 | CD | C | TYR | 13 | CZ | 3.88 | yes |  |  |
| C | ARG | 8 | C | C | ASP | 9 | C | 3.37 | yes |  |  |
| C | ARG | 8 | C | C | ASP | 9 | CB | 3.57 | yes |  |  |
| C | ASP | 9 | C | C | GLY | 10 | C | 3.18 | yes |  |  |
| C | ASP | 9 | CG | C | GLN | 11 | CG | 3.36 | yes |  |  |
| C | GLY | 10 | C | C | GLN | 11 | C | 3.33 | yes |  |  |
| C | GLY | 10 | C | C | GLN | 11 | CB | 3.55 | yes |  |  |
| C | GLY | 10 | C | C | GLN | 11 | CG | 3.67 | yes |  |  |
| C | GLN | 11 | C | C | ALA | 12 | C | 3.25 | yes |  |  |
| C | GLN | 11 | C | C | ALA | 12 | CB | 3.64 | yes |  |  |
| C | GLN | 11 | C | C | LEU | 22 | CD2 | 3.97 | yes |  |  |
| C | GLN | 11 | CB | C | TYR | 13 | CE2 | 3.93 | yes |  |  |
| C | GLN | 11 | CB | C | LEU | 22 | CD2 | 3.47 | yes |  |  |
| C | ALA | 12 | C | C | TYR | 13 | C | 3.47 | yes |  |  |
| C | ALA | 12 | C | C | TYR | 13 | CB | 3.44 | yes |  |  |
| C | ALA | 12 | C | C | TYR | 13 | CG | 3.57 | yes |  |  |
| C | ALA | 12 | C | C | TYR | 13 | CD2 | 3.73 | yes |  |  |
| C | ALA | 12 | C | C | LEU | 22 | CD2 | 3.67 | yes |  |  |
| C | ALA | 12 | CB | C | LEU | 23 | CB | 3.94 | yes |  |  |
| C | TYR | 13 | C | C | VAL | 14 | C | 3.34 | yes |  |  |
| C | TYR | 13 | C | C | VAL | 14 | CB | 3.49 | yes |  |  |
| C | TYR | 13 | C | C | VAL | 14 | CG2 | 3.35 | yes |  |  |
| C | TYR | 13 | C | C | TRP | 20 | CE3 | 3.77 | yes |  |  |
| C | TYR | 13 | CB | C | TRP | 20 | C | 3.99 | yes |  |  |
| C | TYR | 13 | CB | C | TRP | 20 | CB | 3.39 | yes |  |  |
| C | TYR | 13 | CB | C | TRP | 20 | CG | 3.98 | yes |  |  |
| C | TYR | 13 | CB | C | TRP | 20 | CD2 | 3.94 | yes |  |  |
| C | TYR | 13 | CB | C | TRP | 20 | CE3 | 3.45 | yes |  |  |
| C | TYR | 13 | CG | C | TRP | 20 | C | 3.93 | yes |  |  |
| C | TYR | 13 | CG | C | TRP | 20 | CB | 3.47 | yes |  |  |
| C | TYR | 13 | CD1 | C | TRP | 20 | C | 3.23 | yes |  |  |
| C | TYR | 13 | CD1 | C | TRP | 20 | CB | 3.59 | yes |  |  |
| C | TYR | 13 | CD1 | C | VAL | 21 | C | 3.19 | yes |  |  |
| C | TYR | 13 | CE1 | C | TRP | 20 | C | 3.99 | yes |  |  |
| C | TYR | 13 | CE1 | C | VAL | 21 | C | 3.91 | yes |  |  |
| C | TYR | 13 | CE1 | C | LEU | 22 | CG | 3.9 | yes |  |  |
| C | VAL | 14 | C | C | ARG | 15 | C | 3.22 | yes |  |  |
| C | VAL | 14 | C | C | ARG | 15 | CB | 3.56 | yes |  |  |
| C | VAL | 14 | C | C | TRP | 20 | CE3 | 3.46 | yes |  |  |
| C | VAL | 14 | C | C | TRP | 20 | CZ3 | 3.63 | yes |  |  |
| C | VAL | 14 | CG2 | C | VAL | 21 | C | 3.78 | yes |  |  |
| C | VAL | 14 | CG2 | C | LEU | 22 | C | 3.79 | yes |  |  |
| C | VAL | 14 | CG2 | C | LEU | 23 | CB | 3.93 | yes |  |  |
| C | VAL | 14 | CG2 | C | PHE | 26 | CD1 | 3.91 | yes |  |  |
| C | VAL | 14 | CG2 | C | PHE | 26 | CE1 | 3.83 | yes |  |  |
| C | ARG | 15 | C | C | LYS | 16 | C | 3.67 | yes |  |  |
| C | ARG | 15 | C | C | LYS | 16 | CB | 3.24 | yes |  |  |
| C | ARG | 15 | CB | C | TRP | 20 | CZ3 | 3.35 | yes |  |  |
| C | ARG | 15 | CB | C | TRP | 20 | CH2 | 3.58 | yes |  |  |
| C | ARG | 15 | CG | C | TRP | 20 | CE3 | 3.92 | yes |  |  |
| C | ARG | 15 | CG | C | TRP | 20 | CZ2 | 3.5 | yes |  |  |
| C | ARG | 15 | CG | C | TRP | 20 | CZ3 | 3.37 | yes |  |  |
| C | ARG | 15 | CG | C | TRP | 20 | CH2 | 3.14 | yes |  |  |
| C | ARG | 15 | CD | C | GLU | 19 | C | 3.71 | yes |  |  |
| C | ARG | 15 | CD | C | TRP | 20 | CD2 | 3.79 | yes |  |  |
| C | ARG | 15 | CD | C | TRP | 20 | CE2 | 3.74 | yes |  |  |
| C | ARG | 15 | CD | C | TRP | 20 | CE3 | 3.85 | yes |  |  |
| C | ARG | 15 | CD | C | TRP | 20 | CZ2 | 3.75 | yes |  |  |
| C | ARG | 15 | CD | C | TRP | 20 | CZ3 | 3.84 | yes |  |  |
| C | ARG | 15 | CD | C | TRP | 20 | CH2 | 3.79 | yes |  |  |
| C | ARG | 15 | CZ | C | GLY | 18 | C | 2.92 | yes |  |  |
| C | LYS | 16 | C | C | ASP | 17 | C | 2.97 | yes |  |  |
| C | LYS | 16 | C | C | ASP | 17 | CB | 2.99 | yes |  |  |
| C | LYS | 16 | CG | C | ASP | 17 | CB | 3.92 | yes |  |  |
| C | LYS | 16 | CD | C | VAL | 21 | CG2 | 3.72 | yes |  |  |
| C | ASP | 17 | C | C | GLY | 18 | C | 3.04 | yes |  |  |
| C | GLY | 18 | C | C | GLU | 19 | C | 3.57 | yes |  |  |
| C | GLY | 18 | C | C | GLU | 19 | CB | 3.34 | yes |  |  |
| C | GLY | 18 | C | C | GLU | 19 | CG | 3.49 | yes |  |  |
| C | GLU | 19 | C | C | TRP | 20 | C | 3.29 | yes |  |  |
| C | GLU | 19 | C | C | TRP | 20 | CB | 3.6 | yes |  |  |
| C | GLU | 19 | C | C | TRP | 20 | CG | 3.83 | yes |  |  |
| C | TRP | 20 | C | C | VAL | 21 | C | 3.38 | yes |  |  |
| C | TRP | 20 | C | C | VAL | 21 | CB | 3.6 | yes |  |  |
| C | TRP | 20 | C | C | VAL | 21 | CG2 | 3.73 | yes |  |  |
| C | VAL | 21 | C | C | LEU | 22 | C | 2.94 | yes |  |  |
| C | VAL | 21 | C | C | LEU | 22 | CB | 3.65 | yes |  |  |
| C | VAL | 21 | CG1 | C | PHE | 26 | CE1 | 3.84 | yes |  |  |
| C | LEU | 22 | C | C | LEU | 23 | C | 3 | yes |  |  |
| C | LEU | 22 | C | C | LEU | 23 | CB | 3.69 | yes |  |  |
| C | LEU | 23 | C | C | SER | 24 | C | 3.02 | yes |  |  |
| C | LEU | 23 | C | C | SER | 24 | CB | 3.71 | yes |  |  |
| C | SER | 24 | C | C | THR | 25 | C | 2.98 | yes |  |  |
| C | SER | 24 | C | C | THR | 25 | CB | 3.71 | yes |  |  |
| C | THR | 25 | C | C | PHE | 26 | C | 3.36 | yes |  |  |
| C | THR | 25 | C | C | PHE | 26 | CB | 3.59 | yes |  |  |
| C | THR | 25 | C | C | PHE | 26 | CG | 3.81 | yes |  |  |
| C | THR | 25 | C | C | PHE | 26 | CD1 | 3.74 | yes |  |  |
| C | PHE | 26 | C | C | LEU | 27 | C | 3.51 | yes |  |  |
| C | PHE | 26 | C | C | LEU | 27 | CB | 3.41 | yes |  |  |
| C | PHE | 26 | C | C | LEU | 27 | CD1 | 3.55 | yes |  |  |
|  |  |  |  |  |  |  |  | **sums** | **417** | **162** | **579** |

**Table S10:** Van der Waals contacts (no heteroatoms) in the foldon 1 trimer.

| Residue 1 | Atom 1 | Residue 2 | Atom 2 | Distance | intracatenane | intercatenane | total |
| --- | --- | --- | --- | --- | --- | --- | --- |
| GLY 1 (A) | C | TYR 2 (A) | C | 3.09 | yes |  |  |
| GLY 1 (A) | C | TYR 2 (A) | CB | 3.68 | yes |  |  |
| TYR 2 (A) | C | ILE 3 (A) | C | 3.17 | yes |  |  |
| TYR 2 (A) | C | ILE 3 (A) | CB | 3.75 | yes |  |  |
| TYR 2 (A) | C | TYR 2 (C) | CD1 | 3.66 |  | yes |  |
| TYR 2 (A) | C | TYR 2 (C) | CE1 | 3.78 |  | yes |  |
| TYR 2 (A) | CB | TYR 2 (C) | CG | 3.7 |  | yes |  |
| TYR 2 (A) | CB | TYR 2 (C) | CD1 | 3.6 |  | yes |  |
| TYR 2 (A) | CB | TYR 2 (C) | CD2 | 3.99 |  | yes |  |
| TYR 2 (A) | CB | TYR 2 (C) | CE1 | 3.85 |  | yes |  |
| TYR 2 (A) | CG | TYR 2 (B) | CB | 3.65 |  | yes |  |
| TYR 2 (A) | CD1 | TYR 2 (B) | CB | 3.53 |  | yes |  |
| TYR 2 (A) | CD1 | ILE 3 (B) | C | 3.74 |  | yes |  |
| TYR 2 (A) | CD2 | TYR 2 (B) | CD2 | 3.87 |  | yes |  |
| TYR 2 (A) | CE1 | TYR 2 (B) | CB | 3.94 |  | yes |  |
| ILE 3 (A) | C | PRO 4 (A) | C | 3.27 | yes |  |  |
| ILE 3 (A) | C | PRO 4 (A) | CB | 3.57 | yes |  |  |
| ILE 3 (A) | C | PRO 4 (A) | CG | 3.67 | yes |  |  |
| ILE 3 (A) | C | PRO 4 (A) | CD | 2.53 | yes |  |  |
| ILE 3 (A) | C | TYR 2 (C) | CD1 | 3.81 |  | yes |  |
| ILE 3 (A) | CB | ILE 3 (B) | CB | 3.99 |  | yes |  |
| ILE 3 (A) | CG1 | ILE 3 (B) | C | 3.99 |  | yes |  |
| ILE 3 (A) | CG1 | ILE 3 (B) | CB | 3.7 |  | yes |  |
| ILE 3 (A) | CG1 | ILE 3 (B) | CG2 | 3.58 |  | yes |  |
| ILE 3 (A) | CG1 | TRP 20 (B) | CZ2 | 3.63 |  | yes |  |
| ILE 3 (A) | CG2 | PRO 4 (A) | CD | 3.97 | yes |  |  |
| ILE 3 (A) | CG2 | TRP 20 (A) | CZ2 | 3.3 | yes |  |  |
| ILE 3 (A) | CG2 | ILE 3 (C) | CG1 | 3.9 |  | yes |  |
| ILE 3 (A) | CG2 | ILE 3 (C) | CD1 | 3.77 |  | yes |  |
| ILE 3 (A) | CD1 | ARG 15 (A) | CD | 3.85 | yes |  |  |
| ILE 3 (A) | CD1 | ARG 15 (A) | CZ | 3.9 | yes |  |  |
| ILE 3 (A) | CD1 | GLU 5 (B) | CD | 3.99 |  | yes |  |
| ILE 3 (A) | CD1 | TRP 20 (B) | CZ2 | 3.55 |  | yes |  |
| PRO 4 (A) | C | GLU 5 (A) | C | 3.09 | yes |  |  |
| PRO 4 (A) | C | GLU 5 (A) | CB | 3.7 | yes |  |  |
| PRO 4 (A) | C | ILE 3 (C) | CG2 | 3.92 |  | yes |  |
| GLU 5 (A) | C | ALA 6 (A) | C | 3.26 | yes |  |  |
| GLU 5 (A) | C | ALA 6 (A) | CB | 3.66 | yes |  |  |
| GLU 5 (A) | C | TRP 20 (A) | CD1 | 3.85 | yes |  |  |
| GLU 5 (A) | CB | ILE 3 (C) | CG2 | 3.87 |  | yes |  |
| GLU 5 (A) | CD | ILE 3 (C) | CG2 | 3.78 |  | yes |  |
| ALA 6 (A) | C | PRO 7 (A) | C | 3.03 | yes |  |  |
| ALA 6 (A) | C | PRO 7 (A) | CB | 3.61 | yes |  |  |
| ALA 6 (A) | C | PRO 7 (A) | CG | 3.6 | yes |  |  |
| ALA 6 (A) | C | PRO 7 (A) | CD | 2.53 | yes |  |  |
| ALA 6 (A) | CB | PRO 7 (A) | CD | 3.29 | yes |  |  |
| ALA 6 (A) | CB | TRP 20 (A) | CG | 3.65 | yes |  |  |
| ALA 6 (A) | CB | TRP 20 (A) | CD1 | 3.7 | yes |  |  |
| ALA 6 (A) | CB | TRP 20 (A) | CD2 | 3.71 | yes |  |  |
| ALA 6 (A) | CB | TRP 20 (A) | CE2 | 3.8 | yes |  |  |
| ALA 6 (A) | CB | ARG 15 (C) | CG | 3.75 |  | yes |  |
| PRO 7 (A) | C | ARG 8 (A) | C | 3.36 | yes |  |  |
| PRO 7 (A) | C | ARG 8 (A) | CB | 3.66 | yes |  |  |
| PRO 7 (A) | C | ARG 8 (A) | CG | 3.93 | yes |  |  |
| PRO 7 (A) | CB | TYR 13 (A) | CE2 | 3.88 | yes |  |  |
| PRO 7 (A) | CB | TYR 13 (A) | CZ | 3.82 | yes |  |  |
| PRO 7 (A) | CG | TYR 13 (A) | CE1 | 3.89 | yes |  |  |
| PRO 7 (A) | CG | TYR 13 (A) | CE2 | 3.95 | yes |  |  |
| PRO 7 (A) | CG | TYR 13 (A) | CZ | 3.58 | yes |  |  |
| PRO 7 (A) | CD | TYR 13 (A) | CD2 | 3.95 | yes |  |  |
| PRO 7 (A) | CD | TYR 13 (A) | CE1 | 3.91 | yes |  |  |
| PRO 7 (A) | CD | TYR 13 (A) | CE2 | 3.76 | yes |  |  |
| PRO 7 (A) | CD | TYR 13 (A) | CZ | 3.76 | yes |  |  |
| ARG 8 (A) | C | ASP 9 (A) | C | 3.22 | yes |  |  |
| ARG 8 (A) | C | ASP 9 (A) | CB | 3.66 | yes |  |  |
| ASP 9 (A) | CG | TYR 13 (A) | CE2 | 3.96 | yes |  |  |
| GLN 11 (A) | C | ALA 12 (A) | C | 3.24 | yes |  |  |
| GLN 11 (A) | C | ALA 12 (A) | CB | 3.66 | yes |  |  |
| GLN 11 (A) | C | TYR 13 (A) | CD2 | 3.92 | yes |  |  |
| GLN 11 (A) | C | TYR 13 (A) | CE2 | 3.98 | yes |  |  |
| GLN 11 (A) | C | LYS 16 (C) | CG | 3.84 | yes |  |  |
| GLN 11 (A) | CB | LEU 22 (A) | CD2 | 3.39 | yes |  |  |
| GLN 11 (A) | CG | LEU 22 (A) | CD2 | 3.91 | yes |  |  |
| ALA 12 (A) | C | TYR 13 (A) | C | 3.57 | yes |  |  |
| ALA 12 (A) | C | TYR 13 (A) | CB | 3.46 | yes |  |  |
| ALA 12 (A) | C | TYR 13 (A) | CG | 3.62 | yes |  |  |
| ALA 12 (A) | C | TYR 13 (A) | CD2 | 3.55 | yes |  |  |
| ALA 12 (A) | CB | VAL 14 (C) | CG2 | 3.82 |  | yes |  |
| ALA 12 (A) | CB | PHE 26 (C) | CE2 | 3.94 |  | yes |  |
| ALA 12 (A) | CB | PHE 26 (C) | CZ | 3.8 |  | yes |  |
| TYR 13 (A) | C | VAL 14 (A) | C | 3.48 | yes |  |  |
| TYR 13 (A) | C | VAL 14 (A) | CB | 3.55 | yes |  |  |
| TYR 13 (A) | C | VAL 14 (A) | CG1 | 3.62 | yes |  |  |
| TYR 13 (A) | C | TRP 20 (A) | CE3 | 3.95 | yes |  |  |
| TYR 13 (A) | CB | TRP 20 (A) | CE3 | 3.7 | yes |  |  |
| TYR 13 (A) | CD1 | TRP 20 (A) | C | 3.79 | yes |  |  |
| TYR 13 (A) | CD1 | TRP 20 (A) | CB | 3.83 | yes |  |  |
| TYR 13 (A) | CD1 | VAL 21 (A) | C | 3.47 | yes |  |  |
| TYR 13 (A) | CE1 | LEU 22 (A) | CG | 3.85 | yes |  |  |
| TYR 13 (A) | CE1 | LEU 22 (A) | CD2 | 3.71 | yes |  |  |
| TYR 13 (A) | CZ | LEU 22 (A) | CD2 | 3.51 | yes |  |  |
| VAL 14 (A) | C | ARG 15 (A) | C | 3.36 | yes |  |  |
| VAL 14 (A) | C | ARG 15 (A) | CB | 3.57 | yes |  |  |
| VAL 14 (A) | C | TRP 20 (A) | CE3 | 3.61 | yes |  |  |
| VAL 14 (A) | C | TRP 20 (A) | CZ3 | 3.93 | yes |  |  |
| VAL 14 (A) | CG1 | LEU 23 (A) | CD1 | 3.67 | yes |  |  |
| VAL 14 (A) | CG1 | VAL 14 (B) | CG1 | 3.83 |  | yes |  |
| VAL 14 (A) | CG1 | VAL 14 (C) | CG1 | 3.85 |  | yes |  |
| VAL 14 (A) | CG2 | PHE 26 (A) | CD2 | 3.84 | yes |  |  |
| VAL 14 (A) | CG2 | PHE 26 (A) | CE2 | 3.48 | yes |  |  |
| VAL 14 (A) | CG2 | ALA 12 (B) | CB | 3.94 |  | yes |  |
| ARG 15 (A) | C | LYS 16 (A) | C | 3.63 | yes |  |  |
| ARG 15 (A) | C | LYS 16 (A) | CB | 3.24 | yes |  |  |
| ARG 15 (A) | CB | TRP 20 (B) | CZ3 | 3.78 |  | yes |  |
| ARG 15 (A) | CB | TRP 20 (B) | CH2 | 3.85 |  | yes |  |
| ARG 15 (A) | CG | GLU 5 (B) | OE2 | 3.54 |  | yes |  |
| ARG 15 (A) | CG | ALA 6 (B) | CB | 3.77 |  | yes |  |
| ARG 15 (A) | CD | GLU 19 (A) | C | 3.95 | yes |  |  |
| ARG 15 (A) | CZ | GLY 18 (A) | C | 3.54 | yes |  |  |
| LYS 16 (A) | CG | GLN 11 (B) | C | 3.97 |  | yes |  |
| LYS 16 (A) | CD | PHE 26 (A) | CZ | 3.73 | yes |  |  |
| GLY 18 (A) | C | GLU 19 (A) | C | 3.6 | yes |  |  |
| GLY 18 (A) | C | GLU 19 (A) | CB | 3.38 | yes |  |  |
| GLY 18 (A) | C | GLU 19 (A) | CG | 3.38 | yes |  |  |
| GLY 18 (A) | C | GLU 19 (A) | CD | 3.73 | yes |  |  |
| GLY 18 (A) | C | GLU 19 (A) | OE2 | 3.99 | yes |  |  |
| GLU 19 (A) | C | TRP 20 (A) | C | 3.21 | yes |  |  |
| GLU 19 (A) | C | TRP 20 (A) | CB | 3.64 | yes |  |  |
| TRP 20 (A) | C | VAL 21 (A) | C | 3.51 | yes |  |  |
| TRP 20 (A) | C | VAL 21 (A) | CB | 3.47 | yes |  |  |
| TRP 20 (A) | C | VAL 21 (A) | CG2 | 3.44 | yes |  |  |
| TRP 20 (A) | CZ2 | ILE 3 (C) | CD1 | 3.87 |  | yes |  |
| TRP 20 (A) | CZ3 | ARG 15 (C) | CB | 3.79 |  | yes |  |
| TRP 20 (A) | CH2 | ARG 15 (C) | CB | 3.74 |  | yes |  |
| VAL 21 (A) | C | LEU 22 (A) | C | 2.94 | yes |  |  |
| VAL 21 (A) | C | LEU 22 (A) | CB | 3.71 | yes |  |  |
| VAL 21 (A) | CG1 | PHE 26 (A) | CE2 | 3.68 | yes |  |  |
| VAL 21 (A) | CG1 | PHE 26 (A) | CZ | 3.87 | yes |  |  |
| LEU 22 (A) | C | LEU 23 (A) | C | 2.98 | yes |  |  |
| LEU 22 (A) | C | LEU 23 (A) | CB | 3.7 | yes |  |  |
| LEU 23 (A) | C | SER 24 (A) | C | 3.03 | yes |  |  |
| LEU 23 (A) | C | SER 24 (A) | CB | 3.72 | yes |  |  |
| LEU 23 (A) | CG | LEU 27 (A) | CD1 | 3.82 | yes |  |  |
| LEU 23 (A) | CD1 | LEU 23 (B) | CD1 | 3.97 |  | yes |  |
| LEU 23 (A) | CD1 | LEU 23 (B) | CD2 | 3.96 |  | yes |  |
| LEU 23 (A) | CD1 | LEU 23 (C) | CD1 | 3.89 |  | yes |  |
| LEU 23 (A) | CD2 | PHE 26 (C) | CB | 3.62 |  | yes |  |
| LEU 23 (A) | CD2 | PHE 26 (C) | CG | 3.59 |  | yes |  |
| LEU 23 (A) | CD2 | PHE 26 (C) | CD2 | 3.6 |  | yes |  |
| SER 24 (A) | C | THR 25 (A) | C | 3.09 | yes |  |  |
| SER 24 (A) | C | THR 25 (A) | CB | 3.72 | yes |  |  |
| THR 25 (A) | C | PHE 26 (A) | C | 3.4 | yes |  |  |
| THR 25 (A) | C | PHE 26 (A) | CB | 3.61 | yes |  |  |
| THR 25 (A) | C | PHE 26 (A) | CG | 3.79 | yes |  |  |
| PHE 26 (A) | C | LEU 27 (A) | C | 3.42 | yes |  |  |
| PHE 26 (A) | C | LEU 27 (A) | CB | 3.57 | yes |  |  |
| PHE 26 (A) | C | LEU 27 (A) | CG | 3.83 | yes |  |  |
| PHE 26 (A) | C | LEU 27 (A) | CD2 | 3.85 | yes |  |  |
| PHE 26 (A) | CB | LEU 23 (B) | CD2 | 3.49 |  | yes |  |
| PHE 26 (A) | CG | LEU 23 (B) | CD2 | 3.79 |  | yes |  |
| PHE 26 (A) | CE1 | ALA 12 (B) | CB | 3.77 |  | yes |  |
| PHE 26 (A) | CZ | ALA 12 (B) | CB | 3.76 |  | yes |  |
| LEU 27 (A) | CD2 | LEU 27 (B) | CD2 | 3.41 |  | yes |  |
| LEU 27 (A) | CD2 | LEU 27 (C) | CD2 | 3.58 |  | yes |  |
| GLY 1 (B) | C | TYR 2 (B) | C | 3.08 | yes |  |  |
| GLY 1 (B) | C | TYR 2 (B) | CB | 3.7 | yes |  |  |
| TYR 2 (B) | C | ILE 3 (B) | C | 3.46 | yes |  |  |
| TYR 2 (B) | C | ILE 3 (B) | CB | 3.64 | yes |  |  |
| TYR 2 (B) | C | ILE 3 (B) | CG1 | 3.76 | yes |  |  |
| TYR 2 (B) | CG | TYR 2 (C) | CB | 3.78 |  | yes |  |
| TYR 2 (B) | CD1 | TYR 2 (C) | CB | 3.63 |  | yes |  |
| TYR 2 (B) | CE1 | TYR 2 (C) | CB | 3.82 |  | yes |  |
| ILE 3 (B) | C | PRO 4 (B) | C | 3.12 | yes |  |  |
| ILE 3 (B) | C | PRO 4 (B) | CB | 3.64 | yes |  |  |
| ILE 3 (B) | C | PRO 4 (B) | CG | 3.57 | yes |  |  |
| ILE 3 (B) | C | PRO 4 (B) | CD | 2.51 | yes |  |  |
| ILE 3 (B) | CG2 | PRO 4 (B) | CD | 3.77 | yes |  |  |
| ILE 3 (B) | CG2 | ARG 15 (B) | CZ | 3.41 | yes |  |  |
| ILE 3 (B) | CG2 | TRP 20 (B) | CZ2 | 3.54 | yes |  |  |
| ILE 3 (B) | CD1 | PRO 4 (C) | C | 3.98 |  | yes |  |
| ILE 3 (B) | CD1 | TRP 20 (C) | CZ2 | 3.3 |  | yes |  |
| ILE 3 (B) | CD1 | TRP 20 (C) | CH2 | 3.93 |  | yes |  |
| PRO 4 (B) | C | GLU 5 (B) | C | 3 | yes |  |  |
| PRO 4 (B) | C | GLU 5 (B) | CB | 3.7 | yes |  |  |
| GLU 5 (B) | C | ALA 6 (B) | C | 3.09 | yes |  |  |
| GLU 5 (B) | C | ALA 6 (B) | CB | 3.69 | yes |  |  |
| GLU 5 (B) | CG | ARG 8 (B) | CZ | 3.71 | yes |  |  |
| ALA 6 (B) | C | PRO 7 (B) | C | 2.89 | yes |  |  |
| ALA 6 (B) | C | PRO 7 (B) | CB | 3.61 | yes |  |  |
| ALA 6 (B) | C | PRO 7 (B) | CG | 3.63 | yes |  |  |
| ALA 6 (B) | C | PRO 7 (B) | CD | 2.54 | yes |  |  |
| ALA 6 (B) | CB | PRO 7 (B) | CD | 3.35 | yes |  |  |
| ALA 6 (B) | CB | TRP 20 (B) | CG | 3.73 | yes |  |  |
| ALA 6 (B) | CB | TRP 20 (B) | CD1 | 3.86 | yes |  |  |
| ALA 6 (B) | CB | TRP 20 (B) | CD2 | 3.59 | yes |  |  |
| ALA 6 (B) | CB | TRP 20 (B) | CE2 | 3.65 | yes |  |  |
| PRO 7 (B) | C | ARG 8 (B) | C | 3.39 | yes |  |  |
| PRO 7 (B) | C | ARG 8 (B) | CB | 3.56 | yes |  |  |
| PRO 7 (B) | C | ARG 8 (B) | CG | 3.79 | yes |  |  |
| PRO 7 (B) | CB | TYR 13 (B) | CE2 | 3.78 | yes |  |  |
| PRO 7 (B) | CB | TYR 13 (B) | CZ | 3.55 | yes |  |  |
| PRO 7 (B) | CG | TYR 13 (B) | CE1 | 3.68 | yes |  |  |
| PRO 7 (B) | CG | TYR 13 (B) | CZ | 3.55 | yes |  |  |
| PRO 7 (B) | CD | TYR 13 (B) | CD1 | 3.96 | yes |  |  |
| PRO 7 (B) | CD | TYR 13 (B) | CD2 | 3.99 | yes |  |  |
| PRO 7 (B) | CD | TYR 13 (B) | CE1 | 3.87 | yes |  |  |
| PRO 7 (B) | CD | TYR 13 (B) | CE2 | 3.9 | yes |  |  |
| PRO 7 (B) | CD | TYR 13 (B) | CZ | 3.84 | yes |  |  |
| PRO 7 (B) | CD | TRP 20 (B) | CB | 3.76 | yes |  |  |
| ARG 8 (B) | C | ASP 9 (B) | C | 3.39 | yes |  |  |
| ARG 8 (B) | C | ASP 9 (B) | CB | 3.57 | yes |  |  |
| ASP 9 (B) | CG | GLN 11 (B) | CG | 3.84 | yes |  |  |
| GLN 11 (B) | C | ALA 12 (B) | C | 3.27 | yes |  |  |
| GLN 11 (B) | C | ALA 12 (B) | CB | 3.66 | yes |  |  |
| GLN 11 (B) | C | TYR 13 (B) | CD2 | 3.8 | yes |  |  |
| GLN 11 (B) | C | TYR 13 (B) | CE2 | 3.68 | yes |  |  |
| GLN 11 (B) | CB | TYR 13 (B) | CE2 | 3.88 | yes |  |  |
| GLN 11 (B) | CB | LEU 22 (B) | CD2 | 3.51 | yes |  |  |
| GLN 11 (B) | CD | LEU 22 (B) | CD2 | 3.49 | yes |  |  |
| ALA 12 (B) | C | TYR 13 (B) | C | 3.58 | yes |  |  |
| ALA 12 (B) | C | TYR 13 (B) | CB | 3.45 | yes |  |  |
| ALA 12 (B) | C | TYR 13 (B) | CG | 3.52 | yes |  |  |
| ALA 12 (B) | C | TYR 13 (B) | CD2 | 3.47 | yes |  |  |
| TYR 13 (B) | C | VAL 14 (B) | C | 3.5 | yes |  |  |
| TYR 13 (B) | C | VAL 14 (B) | CB | 3.53 | yes |  |  |
| TYR 13 (B) | C | VAL 14 (B) | CG1 | 3.54 | yes |  |  |
| TYR 13 (B) | CB | TRP 20 (B) | CE3 | 3.67 | yes |  |  |
| TYR 13 (B) | CD1 | TRP 20 (B) | C | 3.97 | yes |  |  |
| TYR 13 (B) | CD1 | TRP 20 (B) | CB | 3.91 | yes |  |  |
| TYR 13 (B) | CD1 | VAL 21 (B) | C | 3.59 | yes |  |  |
| TYR 13 (B) | CE1 | LEU 22 (B) | CG | 3.82 | yes |  |  |
| TYR 13 (B) | CE1 | LEU 22 (B) | CD2 | 3.46 | yes |  |  |
| TYR 13 (B) | CZ | LEU 22 (B) | CD2 | 3.51 | yes |  |  |
| VAL 14 (B) | C | ARG 15 (B) | C | 3.36 | yes |  |  |
| VAL 14 (B) | C | ARG 15 (B) | CB | 3.56 | yes |  |  |
| VAL 14 (B) | C | TRP 20 (B) | CE3 | 3.75 | yes |  |  |
| VAL 14 (B) | CG1 | LEU 23 (B) | CD1 | 3.95 | yes |  |  |
| VAL 14 (B) | CG1 | VAL 14 (C) | CG1 | 3.84 |  | yes |  |
| VAL 14 (B) | CG2 | PHE 26 (B) | CE2 | 3.78 | yes |  |  |
| VAL 14 (B) | CG2 | ALA 12 (C) | CB | 3.85 |  | yes |  |
| ARG 15 (B) | C | LYS 16 (B) | C | 3.64 | yes |  |  |
| ARG 15 (B) | C | LYS 16 (B) | CB | 3.23 | yes |  |  |
| ARG 15 (B) | CG | ALA 6 (C) | CB | 3.73 |  | yes |  |
| ARG 15 (B) | CZ | GLY 18 (B) | C | 3.48 |  | yes |  |
| ARG 15 (B) | CZ | GLU 19 (B) | C | 3.94 | yes |  |  |
| LYS 16 (B) | CB | VAL 21 (B) | CG2 | 4 | yes |  |  |
| LYS 16 (B) | CG | GLN 11 (C) | C | 3.78 |  | yes |  |
| GLY 18 (B) | C | GLU 19 (B) | C | 3.57 | yes |  |  |
| GLY 18 (B) | C | GLU 19 (B) | CB | 3.38 | yes |  |  |
| GLY 18 (B) | C | GLU 19 (B) | CG | 3.41 | yes |  |  |
| GLY 18 (B) | C | GLU 19 (B) | CD | 3.75 | yes |  |  |
| GLU 19 (B) | C | TRP 20 (B) | C | 3.23 | yes |  |  |
| GLU 19 (B) | C | TRP 20 (B) | CB | 3.6 | yes |  |  |
| GLU 19 (B) | C | TRP 20 (B) | CG | 3.91 | yes |  |  |
| TRP 20 (B) | C | VAL 21 (B) | C | 3.49 | yes |  |  |
| TRP 20 (B) | C | VAL 21 (B) | CB | 3.54 | yes |  |  |
| TRP 20 (B) | C | VAL 21 (B) | CG2 | 3.57 | yes |  |  |
| VAL 21 (B) | C | LEU 22 (B) | C | 2.98 | yes |  |  |
| VAL 21 (B) | C | LEU 22 (B) | CB | 3.69 | yes |  |  |
| VAL 21 (B) | CG1 | PHE 26 (B) | CE2 | 3.62 | yes |  |  |
| LEU 22 (B) | C | LEU 23 (B) | C | 2.97 | yes |  |  |
| LEU 22 (B) | C | LEU 23 (B) | CB | 3.69 | yes |  |  |
| LEU 23 (B) | C | SER 24 (B) | C | 3.02 | yes |  |  |
| LEU 23 (B) | C | SER 24 (B) | CB | 3.72 | yes |  |  |
| LEU 23 (B) | CD1 | LEU 23 (C) | CD1 | 3.84 |  | yes |  |
| LEU 23 (B) | CD1 | LEU 23 (C) | CD2 | 4 |  | yes |  |
| SER 24 (B) | C | THR 25 (B) | C | 3.1 | yes |  |  |
| SER 24 (B) | C | THR 25 (B) | CB | 3.73 | yes |  |  |
| THR 25 (B) | C | PHE 26 (B) | C | 3.41 | yes |  |  |
| THR 25 (B) | C | PHE 26 (B) | CB | 3.63 | yes |  |  |
| THR 25 (B) | C | PHE 26 (B) | CG | 3.89 | yes |  |  |
| PHE 26 (B) | C | LEU 27 (B) | C | 3.16 | yes |  |  |
| PHE 26 (B) | C | LEU 27 (B) | CB | 3.68 | yes |  |  |
| PHE 26 (B) | CB | LEU 23 (C) | CD2 | 3.83 |  | yes |  |
| PHE 26 (B) | CB | LEU 27 (C) | CD1 | 3.77 |  | yes |  |
| PHE 26 (B) | CG | LEU 23 (C) | CD2 | 3.76 |  | yes |  |
| PHE 26 (B) | CD2 | LEU 23 (C) | CD2 | 3.86 |  | yes |  |
| PHE 26 (B) | CZ | ALA 12 (C) | CB | 3.91 |  | yes |  |
| GLY 1 (C) | C | TYR 2 (C) | C | 3.11 | yes |  |  |
| GLY 1 (C) | C | TYR 2 (C) | CB | 3.69 | yes |  |  |
| TYR 2 (C) | C | ILE 3 (C) | C | 3.3 | yes |  |  |
| TYR 2 (C) | C | ILE 3 (C) | CB | 3.64 | yes |  |  |
| TYR 2 (C) | C | ILE 3 (C) | CG2 | 3.85 | yes |  |  |
| ILE 3 (C) | C | PRO 4 (C) | C | 3.16 | yes |  |  |
| ILE 3 (C) | C | PRO 4 (C) | CB | 3.62 | yes |  |  |
| ILE 3 (C) | C | PRO 4 (C) | CG | 3.6 | yes |  |  |
| ILE 3 (C) | C | PRO 4 (C) | CD | 2.53 | yes |  |  |
| ILE 3 (C) | CB | PRO 4 (C) | CD | 3.31 | yes |  |  |
| ILE 3 (C) | CD1 | ARG 15 (C) | CD | 3.83 | yes |  |  |
| ILE 3 (C) | CD1 | ARG 15 (C) | CZ | 3.91 | yes |  |  |
| ILE 3 (C) | CD1 | TRP 20 (C) | CZ2 | 3.55 | yes |  |  |
| PRO 4 (C) | C | GLU 5 (C) | C | 2.97 | yes |  |  |
| PRO 4 (C) | C | GLU 5 (C) | CB | 3.69 | yes |  |  |
| GLU 5 (C) | C | ALA 6 (C) | C | 3.09 | yes |  |  |
| GLU 5 (C) | C | ALA 6 (C) | CB | 3.7 | yes |  |  |
| ALA 6 (C) | C | PRO 7 (C) | C | 2.95 | yes |  |  |
| ALA 6 (C) | C | PRO 7 (C) | CB | 3.63 | yes |  |  |
| ALA 6 (C) | C | PRO 7 (C) | CG | 3.62 | yes |  |  |
| ALA 6 (C) | C | PRO 7 (C) | CD | 2.55 | yes |  |  |
| ALA 6 (C) | CB | PRO 7 (C) | CD | 3.32 | yes |  |  |
| ALA 6 (C) | CB | TRP 20 (C) | CG | 3.82 | yes |  |  |
| ALA 6 (C) | CB | TRP 20 (C) | CD1 | 3.99 | yes |  |  |
| ALA 6 (C) | CB | TRP 20 (C) | CD2 | 3.64 | yes |  |  |
| ALA 6 (C) | CB | TRP 20 (C) | CE2 | 3.72 | yes |  |  |
| PRO 7 (C) | C | ARG 8 (C) | C | 3.42 | yes |  |  |
| PRO 7 (C) | C | ARG 8 (C) | CB | 3.56 | yes |  |  |
| PRO 7 (C) | C | ARG 8 (C) | CG | 3.69 | yes |  |  |
| PRO 7 (C) | CB | TYR 13 (C) | CE2 | 3.69 | yes |  |  |
| PRO 7 (C) | CB | TYR 13 (C) | CZ | 3.63 | yes |  |  |
| PRO 7 (C) | CG | TYR 13 (C) | CE1 | 3.67 | yes |  |  |
| PRO 7 (C) | CG | TYR 13 (C) | CE2 | 3.86 | yes |  |  |
| PRO 7 (C) | CG | TYR 13 (C) | CZ | 3.48 | yes |  |  |
| PRO 7 (C) | CD | TYR 13 (C) | CG | 3.94 | yes |  |  |
| PRO 7 (C) | CD | TYR 13 (C) | CD1 | 3.92 | yes |  |  |
| PRO 7 (C) | CD | TYR 13 (C) | CD2 | 3.91 | yes |  |  |
| PRO 7 (C) | CD | TYR 13 (C) | CE1 | 3.9 | yes |  |  |
| PRO 7 (C) | CD | TYR 13 (C) | CE2 | 3.89 | yes |  |  |
| PRO 7 (C) | CD | TYR 13 (C) | CZ | 3.89 | yes |  |  |
| PRO 7 (C) | CD | TRP 20 (C) | CB | 3.83 | yes |  |  |
| ARG 8 (C) | C | ASP 9 (C) | C | 3.28 | yes |  |  |
| ARG 8 (C) | C | ASP 9 (C) | CB | 3.66 | yes |  |  |
| ASP 9 (C) | CG | GLN 11 (C) | CG | 3.9 | yes |  |  |
| GLN 11 (C) | C | ALA 12 (C) | C | 3.31 | yes |  |  |
| GLN 11 (C) | C | ALA 12 (C) | CB | 3.63 | yes |  |  |
| GLN 11 (C) | C | TYR 13 (C) | CD2 | 3.91 | yes |  |  |
| GLN 11 (C) | C | TYR 13 (C) | CE2 | 3.94 | yes |  |  |
| GLN 11 (C) | CB | TYR 13 (C) | CE2 | 3.97 | yes |  |  |
| GLN 11 (C) | CB | LEU 22 (C) | CD2 | 3.52 | yes |  |  |
| GLN 11 (C) | CD | LEU 22 (C) | CD2 | 3.58 | yes |  |  |
| ALA 12 (C) | C | TYR 13 (C) | C | 3.58 | yes |  |  |
| ALA 12 (C) | C | TYR 13 (C) | CB | 3.46 | yes |  |  |
| ALA 12 (C) | C | TYR 13 (C) | CG | 3.61 | yes |  |  |
| ALA 12 (C) | C | TYR 13 (C) | CD2 | 3.48 | yes |  |  |
| TYR 13 (C) | C | VAL 14 (C) | C | 3.52 | yes |  |  |
| TYR 13 (C) | C | VAL 14 (C) | CB | 3.48 | yes |  |  |
| TYR 13 (C) | C | VAL 14 (C) | CG1 | 3.46 | yes |  |  |
| TYR 13 (C) | CB | TRP 20 (C) | CE3 | 3.79 | yes |  |  |
| TYR 13 (C) | CD1 | TRP 20 (C) | C | 3.86 | yes |  |  |
| TYR 13 (C) | CD1 | TRP 20 (C) | CB | 3.85 | yes |  |  |
| TYR 13 (C) | CD1 | VAL 21 (C) | C | 3.41 | yes |  |  |
| TYR 13 (C) | CE1 | LEU 22 (C) | CG | 3.87 | yes |  |  |
| TYR 13 (C) | CE1 | LEU 22 (C) | CD2 | 3.67 | yes |  |  |
| TYR 13 (C) | CE2 | LEU 22 (C) | CD2 | 3.89 | yes |  |  |
| TYR 13 (C) | CZ | LEU 22 (C) | CD2 | 3.45 | yes |  |  |
| VAL 14 (C) | C | ARG 15 (C) | C | 3.34 | yes |  |  |
| VAL 14 (C) | C | ARG 15 (C) | CB | 3.58 | yes |  |  |
| VAL 14 (C) | C | TRP 20 (C) | CE3 | 3.65 | yes |  |  |
| VAL 14 (C) | C | TRP 20 (C) | CZ3 | 3.98 | yes |  |  |
| VAL 14 (C) | CG1 | LEU 23 (C) | CD1 | 3.91 | yes |  |  |
| VAL 14 (C) | CG2 | VAL 21 (C) | CG2 | 3.65 | yes |  |  |
| VAL 14 (C) | CG2 | PHE 26 (C) | CE2 | 3.87 | yes |  |  |
| ARG 15 (C) | C | LYS 16 (C) | C | 3.66 | yes |  |  |
| ARG 15 (C) | C | LYS 16 (C) | CB | 3.21 | yes |  |  |
| ARG 15 (C) | CD | GLU 19 (C) | C | 3.94 | yes |  |  |
| ARG 15 (C) | CZ | GLY 18 (C) | C | 3.56 | yes |  |  |
| ARG 15 (C) | CZ | GLU 19 (C) | C | 4 | yes |  |  |
| LYS 16 (C) | CB | VAL 21 (C) | CG2 | 3.87 | yes |  |  |
| LYS 16 (C) | CD | VAL 21 (C) | CG2 | 3.96 | yes |  |  |
| LYS 16 (C) | CD | PHE 26 (C) | CZ | 4 | yes |  |  |
| GLY 18 (C) | C | GLU 19 (C) | C | 3.61 | yes |  |  |
| GLY 18 (C) | C | GLU 19 (C) | CB | 3.32 | yes |  |  |
| GLY 18 (C) | C | GLU 19 (C) | CG | 3.32 | yes |  |  |
| GLY 18 (C) | C | GLU 19 (C) | CD | 3.69 | yes |  |  |
| GLU 19 (C) | C | TRP 20 (C) | C | 3.23 | yes |  |  |
| GLU 19 (C) | C | TRP 20 (C) | CB | 3.64 | yes |  |  |
| TRP 20 (C) | C | VAL 21 (C) | C | 3.53 | yes |  |  |
| TRP 20 (C) | C | VAL 21 (C) | CB | 3.44 | yes |  |  |
| TRP 20 (C) | C | VAL 21 (C) | CG1 | 3.41 | yes |  |  |
| VAL 21 (C) | C | LEU 22 (C) | C | 3.03 | yes |  |  |
| VAL 21 (C) | C | LEU 22 (C) | CB | 3.68 | yes |  |  |
| VAL 21 (C) | CG2 | PHE 26 (C) | CE2 | 3.9 | yes |  |  |
| LEU 22 (C) | C | LEU 23 (C) | C | 3 | yes |  |  |
| LEU 22 (C) | C | LEU 23 (C) | CB | 3.71 | yes |  |  |
| LEU 23 (C) | C | SER 24 (C) | C | 3.04 | yes |  |  |
| LEU 23 (C) | C | SER 24 (C) | CB | 3.71 | yes |  |  |
| LEU 23 (C) | CG | LEU 27 (C) | CD1 | 3.58 | yes |  |  |
| LEU 23 (C) | CD2 | LEU 27 (C) | CD1 | 3.78 | yes |  |  |
| SER 24 (C) | C | THR 25 (C) | C | 2.97 | yes |  |  |
| SER 24 (C) | C | THR 25 (C) | CB | 3.73 | yes |  |  |
| THR 25 (C) | C | PHE 26 (C) | C | 3.44 | yes |  |  |
| THR 25 (C) | C | PHE 26 (C) | CB | 3.56 | yes |  |  |
| THR 25 (C) | C | PHE 26 (C) | CG | 3.68 | yes |  |  |
| THR 25 (C) | C | PHE 26 (C) | CD2 | 3.96 | yes |  |  |
| THR 25 (C) | CB | PHE 26 (C) | CD2 | 3.62 | yes |  |  |
| THR 25 (C) | CB | PHE 26 (C) | CE2 | 3.62 | yes |  |  |
| THR 25 (C) | CG2 | PHE 26 (C) | CZ | 3.97 | yes |  |  |
| PHE 26 (C) | C | LEU 27 (C) | C | 3.43 | yes |  |  |
| PHE 26 (C) | C | LEU 27 (C) | CB | 3.55 | yes |  |  |
| PHE 26 (C) | C | LEU 27 (C) | CG | 3.8 | yes |  |  |
| PHE 26 (C) | C | LEU 27 (C) | CD2 | 3.89 | yes |  |  |
|  |  |  |  | **sums** | **311** | **69** | **380** |

**Table S11:** Intercatenane Cα atom distances of the foldon 0 trimer.

| **aa** | **dAB (Å)** | **dBC (Å)** | **dCA (Å)** | **avg. d (Å)** | **std. dev. d** |
| --- | --- | --- | --- | --- | --- |
| G1 | 13.374 | 13.383 | 12.769 | 13.175 | 0.352 |
| Y2 | 7.502 | 7.223 | 7.045 | 7.257 | 0.230 |
| I3 | 7.164 | 7.086 | 6.988 | 7.079 | 0.088 |
| P4 | 9.384 | 9.340 | 9.349 | 9.358 | 0.023 |
| D5 | 10.669 | 10.374 | 10.544 | 10.529 | 0.148 |
| A6 | 13.406 | 13.168 | 13.261 | 13.278 | 0.120 |
| P7 | 19.657 | 19.597 | 19.426 | 19.560 | 0.120 |
| R8 | 22.508 | 22.552 | 22.155 | 22.405 | 0.218 |
| D9 | 25.713 | 25.985 | 25.679 | 25.792 | 0.168 |
| G10 | 23.481 | 23.381 | 23.289 | 23.384 | 0.096 |
| Q11 | 18.684 | 18.668 | 18.767 | 18.706 | 0.053 |
| A12 | 12.270 | 12.229 | 12.152 | 12.217 | 0.060 |
| Y13 | 9.211 | 9.133 | 9.140 | 9.161 | 0.043 |
| V14 | 5.615 | 5.571 | 5.701 | 5.629 | 0.066 |
| R15 | 8.049 | 7.963 | 8.082 | 8.031 | 0.061 |
| K16 | 14.367 | 14.218 | 14.511 | 14.365 | 0.147 |
| D17 | 19.573 | 19.662 | 19.976 | 19.737 | 0.212 |
| G18 | 16.276 | 16.348 | 16.594 | 16.406 | 0.167 |
| E19 | 15.836 | 15.868 | 16.048 | 15.917 | 0.114 |
| W20 | 12.828 | 12.791 | 12.896 | 12.838 | 0.053 |
| V21 | 14.020 | 13.991 | 14.000 | 14.004 | 0.015 |
| L22 | 13.864 | 13.658 | 13.815 | 13.779 | 0.108 |
| L23 | 8.303 | 8.089 | 8.303 | 8.232 | 0.124 |
| S24 | 11.754 | 11.891 | 12.130 | 11.925 | 0.190 |
| T25 | 14.091 | 14.377 | 14.669 | 14.379 | 0.289 |
| F26 | 9.119 | 9.268 | 9.460 | 9.282 | 0.171 |
| L27 | 7.600 | 7.836 | 7.647 | 7.694 | 0.125 |

**Table S12:** Intercatenane Cα atom distances of the foldon 1 trimer.

| **aa** | **dAB (Å)** | **dBC (Å)** | **dCA (Å)** | **avg d (Å)** | **std. dev. d** |
| --- | --- | --- | --- | --- | --- |
| G1 | 13.181 | 12.366 | 11.786 | 12.444 | 0.701 |
| Y2 | 6.195 | 6.245 | 6.282 | 6.241 | 0.044 |
| I3 | 5.551 | 7.142 | 6.274 | 6.322 | 0.797 |
| P4 | 9.833 | 10.477 | 9.198 | 9.836 | 0.640 |
| D5 | 12.288 | 11.805 | 11.566 | 11.886 | 0.368 |
| A6 | 14.010 | 13.497 | 13.432 | 13.646 | 0.317 |
| P7 | 20.369 | 19.726 | 19.684 | 19.926 | 0.384 |
| R8 | 23.728 | 23.030 | 23.463 | 23.407 | 0.352 |
| D9 | 26.255 | 26.290 | 26.112 | 26.219 | 0.094 |
| DAL10 | 24.033 | 24.131 | 23.974 | 24.046 | 0.079 |
| Q11 | 19.297 | 19.326 | 19.213 | 19.279 | 0.059 |
| A12 | 12.814 | 12.806 | 12.710 | 12.777 | 0.058 |
| Y13 | 9.683 | 9.628 | 9.561 | 9.624 | 0.061 |
| V14 | 5.789 | 5.851 | 5.701 | 5.780 | 0.075 |
| R15 | 8.750 | 9.031 | 8.944 | 8.908 | 0.144 |
| K16 | 15.139 | 15.312 | 15.288 | 15.246 | 0.094 |
| DPN17 | 20.407 | 20.761 | 20.627 | 20.598 | 0.179 |
| G18 | 17.286 | 18.018 | 17.712 | 17.672 | 0.368 |
| E19 | 16.605 | 17.156 | 16.655 | 16.805 | 0.305 |
| W20 | 13.414 | 13.792 | 13.282 | 13.496 | 0.265 |
| V21 | 15.179 | 15.219 | 15.142 | 15.180 | 0.039 |
| L22 | 14.709 | 14.611 | 14.479 | 14.600 | 0.115 |
| L23 | 9.099 | 9.006 | 9.006 | 9.037 | 0.054 |
| S24 | 12.634 | 12.861 | 12.672 | 12.722 | 0.122 |
| T25 | 15.593 | 15.570 | 15.314 | 15.492 | 0.155 |
| F26 | 11.150 | 10.695 | 11.040 | 10.962 | 0.237 |
| L27 | 7.509 | 6.925 | 7.961 | 7.465 | 0.519 |

**Table S13:** SASA differences between foldon 0 and foldon 1 trimers.

| **residue** | **resno** | **SASA (Å^2^)** | |  | **ΔSASA (Å^2^)** | **SASA foldon 1 (%)** | **Δ%** |
| --- | --- | --- | --- | --- | --- | --- | --- |
|  |  | **foldon 1** | **foldon 0** |  | **(foldon 1 - foldon 0)** | **(% of foldon 0)** | **(foldon 0 vs. 1)** |
| GLY | 1 | 241.68 | 316.64 |  | -74.96 | 76.33 | 23.67 |
| TYR | 2 | 368.26 | 282.59 |  | 85.67 | 130.32 | -30.32 |
| ILE | 3 | 4.40 | 0.01 |  | 4.39 | 44000.00 | -43900.00 |
| PRO | 4 | 194.69 | 183.45 |  | 11.24 | 106.13 | -6.13 |
| GLU | 5 | 154.21 | 145.91 |  | 8.30 | 105.69 | -5.69 |
| ALA | 6 | 19.29 | 1.01 |  | 18.28 | 1909.90 | -1809.90 |
| PRO | 7 | 159.97 | 214.48 |  | -54.51 | 74.59 | 25.41 |
| ARG | 8 | 500.70 | 485.00 |  | 15.70 | 103.24 | -3.24 |
| ASP | 9 | 257.67 | 265.70 |  | -8.03 | 96.98 | 3.02 |
| DAL/GLY | 10 | 130.15 | 95.97 |  | 34.18 | 135.62 | -35.62 |
| GLN | 11 | 255.07 | 230.82 |  | 24.25 | 110.51 | -10.51 |
| ALA | 12 | 32.35 | 2.48 |  | 29.87 | 1304.44 | -1204.44 |
| TYR | 13 | 48.37 | 52.84 |  | -4.47 | 91.54 | 8.46 |
| VAL | 14 | 4.03 | 0.09 |  | 3.94 | 4477.78 | -4377.78 |
| ARG | 15 | 27.93 | 1.33 |  | 26.60 | 2100.00 | -2000.00 |
| LYS | 16 | 206.18 | 167.22 |  | 38.96 | 123.30 | -23.30 |
| DPN/ASP | 17 | 330.73 | 143.32 |  | 187.41 | 230.76 | -130.76 |
| GLY | 18 | 71.45 | 35.51 |  | 35.94 | 201.21 | -101.21 |
| GLU | 19 | 338.27 | 366.87 |  | -28.60 | 92.20 | 7.80 |
| TRP | 20 | 119.05 | 82.81 |  | 36.24 | 143.76 | -43.76 |
| VAL | 21 | 114.10 | 104.02 |  | 10.08 | 109.69 | -9.69 |
| LEU | 22 | 160.90 | 119.43 |  | 41.47 | 134.72 | -34.72 |
| LEU | 23 | 10.19 | 0.09 |  | 10.10 | 11322.22 | -11222.22 |
| SER | 24 | 218.96 | 179.13 |  | 39.83 | 122.24 | -22.24 |
| THR | 25 | 290.57 | 350.75 |  | -60.18 | 82.84 | 17.16 |
| PHE | 26 | 206.93 | 98.50 |  | 108.43 | 210.08 | -110.08 |
| LEU | 27 | 358.33 | 379.25 |  | -20.92 | 94.48 | 5.52 |
|  | **sums** | **4824.43** | **4305.22** |  |  |  |  |

**Table S14:** Hydrogen bonds in the foldon 0 trimer.

| chain1 | residue1 | res_id1 | atom1 | chain2 | residue2 | res_id2 | atom2 | Distance [Å] | intracatenane | intercatenane | total |
| --- | --- | --- | --- | --- | --- | --- | --- | --- | --- | --- | --- |
| A | GLY | 1 | N | A | TYR | 2 | N | 2.92 | yes |  |  |
| A | GLY | 1 | O | A | TYR | 2 | N | 2.25 | yes |  |  |
| A | GLY | 1 | O | C | GLU | 5 | N | 2.45 |  | yes |  |
| A | TYR | 2 | O | A | ILE | 3 | N | 2.26 | yes |  |  |
| A | TYR | 2 | O | B | TYR | 2 | OH | 3 |  | yes |  |
| A | TYR | 2 | OH | C | TYR | 2 | O | 3.49 |  | yes |  |
| A | ILE | 3 | N | C | GLU | 5 | N | 3.45 |  | yes |  |
| A | ILE | 3 | O | A | PRO | 4 | N | 2.25 | yes |  |  |
| A | PRO | 4 | O | A | GLU | 5 | N | 2.25 | yes |  |  |
| A | PRO | 4 | O | A | GLU | 5 | O | 3.39 | yes |  |  |
| A | PRO | 4 | O | A | TRP | 20 | NE1 | 2.39 | yes |  |  |
| A | GLU | 5 | N | B | GLY | 1 | O | 2.51 |  | yes |  |
| A | GLU | 5 | O | A | ALA | 6 | N | 2.27 | yes |  |  |
| A | GLU | 5 | OE2 | B | ARG | 15 | NE | 2.85 |  | yes |  |
| A | GLU | 5 | OE2 | B | ARG | 15 | NH2 | 2.29 |  | yes |  |
| A | ALA | 6 | N | A | TRP | 20 | NE1 | 3.13 | yes |  |  |
| A | ALA | 6 | N | B | ARG | 15 | NH2 | 3.15 |  | yes |  |
| A | ALA | 6 | O | A | PRO | 7 | N | 2.25 | yes |  |  |
| A | ALA | 6 | O | A | PRO | 7 | O | 3.06 | yes |  |  |
| A | ALA | 6 | O | A | ARG | 8 | NE | 2.91 | yes |  |  |
| A | ALA | 6 | O | A | ARG | 8 | NH2 | 3.42 | yes |  |  |
| A | ALA | 6 | O | B | ARG | 15 | NH2 | 2.47 |  | yes |  |
| A | PRO | 7 | O | A | ARG | 8 | N | 2.26 | yes |  |  |
| A | ARG | 8 | N | A | ASP | 9 | N | 2.9 | yes |  |  |
| A | ARG | 8 | O | A | ASP | 9 | N | 2.24 | yes |  |  |
| A | ASP | 9 | N | A | GLY | 10 | N | 2.67 | yes |  |  |
| A | ASP | 9 | O | A | GLY | 10 | N | 2.25 | yes |  |  |
| A | ASP | 9 | O | A | GLN | 11 | N | 3.48 | yes |  |  |
| A | ASP | 9 | OD1 | A | GLN | 11 | N | 3.36 | yes |  |  |
| A | ASP | 9 | OD2 | A | GLN | 11 | OE1 | 3.09 | yes |  |  |
| A | GLY | 10 | N | A | GLN | 11 | N | 2.71 | yes |  |  |
| A | GLY | 10 | O | A | GLN | 11 | N | 2.25 | yes |  |  |
| A | GLN | 11 | O | A | ALA | 12 | N | 2.24 | yes |  |  |
| A | ALA | 12 | O | A | TYR | 13 | N | 2.25 | yes |  |  |
| A | ALA | 12 | O | A | LEU | 23 | N | 2.69 | yes |  |  |
| A | TYR | 13 | N | B | ARG | 15 | O | 2.44 |  | yes |  |
| A | TYR | 13 | O | A | VAL | 14 | N | 2.24 | yes |  |  |
| A | TYR | 13 | O | B | ARG | 15 | N | 2.67 |  | yes |  |
| A | VAL | 14 | N | A | VAL | 21 | O | 2.63 | yes |  |  |
| A | VAL | 14 | O | A | ARG | 15 | N | 2.23 | yes |  |  |
| A | VAL | 14 | O | A | GLU | 19 | O | 3.25 | yes |  |  |
| A | VAL | 14 | O | A | VAL | 21 | N | 2.93 | yes |  |  |
| A | ARG | 15 | N | A | LYS | 16 | N | 3.3 | yes |  |  |
| A | ARG | 15 | N | C | TYR | 13 | O | 2.71 |  | yes |  |
| A | ARG | 15 | O | A | LYS | 16 | N | 2.27 | yes |  |  |
| A | ARG | 15 | O | C | GLN | 11 | O | 3.33 |  | yes |  |
| A | ARG | 15 | O | C | TYR | 13 | N | 2.54 |  | yes |  |
| A | ARG | 15 | NE | A | GLU | 19 | N | 3.45 | yes |  |  |
| A | ARG | 15 | NE | C | GLU | 5 | OE2 | 2.97 |  | yes |  |
| A | ARG | 15 | NH1 | A | GLY | 18 | O | 3.26 | yes |  |  |
| A | ARG | 15 | NH1 | A | TRP | 20 | NE1 | 3.24 | yes |  |  |
| A | ARG | 15 | NH2 | A | GLY | 18 | O | 2.57 | yes |  |  |
| A | ARG | 15 | NH2 | C | GLU | 5 | OE2 | 2.26 |  | yes |  |
| A | LYS | 16 | N | A | GLU | 19 | O | 3.16 | yes |  |  |
| A | LYS | 16 | O | A | ASP | 17 | N | 2.24 | yes |  |  |
| A | LYS | 16 | O | A | ASP | 17 | O | 3.3 | yes |  |  |
| A | LYS | 16 | O | A | ASP | 17 | OD1 | 2.32 | yes |  |  |
| A | LYS | 16 | O | A | GLY | 18 | N | 3.04 | yes |  |  |
| A | LYS | 16 | O | A | GLU | 19 | N | 2.78 | yes |  |  |
| A | LYS | 16 | O | A | GLU | 19 | O | 3.03 | yes |  |  |
| A | ASP | 17 | N | A | GLY | 18 | N | 2.86 | yes |  |  |
| A | ASP | 17 | O | A | GLY | 18 | N | 2.25 | yes |  |  |
| A | ASP | 17 | O | A | GLU | 19 | N | 3.05 | yes |  |  |
| A | GLY | 18 | N | A | GLU | 19 | N | 2.77 | yes |  |  |
| A | GLY | 18 | O | A | GLU | 19 | N | 2.24 | yes |  |  |
| A | GLU | 19 | O | A | TRP | 20 | N | 2.25 | yes |  |  |
| A | TRP | 20 | N | A | VAL | 21 | N | 3.35 | yes |  |  |
| A | TRP | 20 | O | A | VAL | 21 | N | 2.25 | yes |  |  |
| A | VAL | 21 | N | A | LEU | 22 | N | 3.47 | yes |  |  |
| A | VAL | 21 | O | A | LEU | 22 | N | 2.23 | yes |  |  |
| A | VAL | 21 | O | A | LEU | 22 | O | 3.37 | yes |  |  |
| A | VAL | 21 | O | A | LEU | 23 | N | 3.05 | yes |  |  |
| A | LEU | 22 | N | A | LEU | 23 | N | 3.41 | yes |  |  |
| A | LEU | 22 | O | A | LEU | 23 | N | 2.24 | yes |  |  |
| A | LEU | 22 | O | A | LEU | 23 | O | 3.32 | yes |  |  |
| A | LEU | 22 | O | A | SER | 24 | N | 3.39 | yes |  |  |
| A | LEU | 22 | O | A | THR | 25 | N | 2.97 | yes |  |  |
| A | LEU | 22 | O | A | THR | 25 | OG1 | 3.49 | yes |  |  |
| A | LEU | 23 | N | A | SER | 24 | N | 2.78 | yes |  |  |
| A | LEU | 23 | O | A | SER | 24 | N | 2.25 | yes |  |  |
| A | LEU | 23 | O | A | THR | 25 | N | 2.82 | yes |  |  |
| A | LEU | 23 | O | A | PHE | 26 | N | 2.5 | yes |  |  |
| A | LEU | 23 | O | A | LEU | 27 | N | 3.41 | yes |  |  |
| A | SER | 24 | N | A | THR | 25 | N | 2.71 | yes |  |  |
| A | SER | 24 | O | A | THR | 25 | N | 2.25 | yes |  |  |
| A | SER | 24 | O | A | PHE | 26 | N | 3.13 | yes |  |  |
| A | SER | 24 | O | A | LEU | 27 | N | 2.9 | yes |  |  |
| A | SER | 24 | O | A | LEU | 27 | OXT | 2.94 | yes |  |  |
| A | SER | 24 | OG | A | THR | 25 | N | 3.18 | yes |  |  |
| A | THR | 25 | N | A | PHE | 26 | N | 2.63 | yes |  |  |
| A | THR | 25 | O | A | PHE | 26 | N | 2.26 | yes |  |  |
| A | PHE | 26 | N | A | LEU | 27 | N | 2.64 | yes |  |  |
| A | PHE | 26 | O | A | LEU | 27 | N | 2.22 | yes |  |  |
| B | GLY | 1 | O | B | TYR | 2 | N | 2.25 | yes |  |  |
| B | TYR | 2 | O | B | ILE | 3 | N | 2.25 | yes |  |  |
| B | ILE | 3 | O | B | PRO | 4 | N | 2.25 | yes |  |  |
| B | PRO | 4 | O | B | GLU | 5 | N | 2.25 | yes |  |  |
| B | PRO | 4 | O | B | GLU | 5 | O | 3.18 | yes |  |  |
| B | PRO | 4 | O | B | TRP | 20 | NE1 | 2.64 | yes |  |  |
| B | GLU | 5 | N | C | GLY | 1 | O | 2.56 |  | yes |  |
| B | GLU | 5 | N | C | ILE | 3 | N | 3.46 |  | yes |  |
| B | GLU | 5 | O | B | ALA | 6 | N | 2.26 | yes |  |  |
| B | GLU | 5 | OE1 | B | ARG | 8 | NE | 3.39 | yes |  |  |
| B | GLU | 5 | OE1 | B | ARG | 8 | NH2 | 2.64 | yes |  |  |
| B | GLU | 5 | OE2 | B | ARG | 8 | NE | 3.42 | yes |  |  |
| B | GLU | 5 | OE2 | C | ARG | 15 | NE | 2.96 |  | yes |  |
| B | GLU | 5 | OE2 | C | ARG | 15 | NH2 | 2.28 |  | yes |  |
| B | ALA | 6 | N | B | TRP | 20 | NE1 | 3.21 | yes |  |  |
| B | ALA | 6 | O | B | PRO | 7 | N | 2.26 | yes |  |  |
| B | ALA | 6 | O | B | PRO | 7 | O | 2.87 | yes |  |  |
| B | PRO | 7 | O | B | ARG | 8 | N | 2.26 | yes |  |  |
| B | ARG | 8 | N | B | ASP | 9 | N | 2.97 | yes |  |  |
| B | ARG | 8 | O | B | ASP | 9 | N | 2.24 | yes |  |  |
| B | ARG | 8 | O | C | ASP | 17 | OD1 | 3.37 |  | yes |  |
| B | ARG | 8 | NE | C | GLY | 18 | N | 3.39 |  | yes |  |
| B | ASP | 9 | N | B | GLY | 10 | N | 2.67 | yes |  |  |
| B | ASP | 9 | N | C | ASP | 17 | OD1 | 3.36 |  | yes |  |
| B | ASP | 9 | O | B | GLY | 10 | N | 2.25 | yes |  |  |
| B | ASP | 9 | O | B | GLN | 11 | N | 3.34 | yes |  |  |
| B | ASP | 9 | OD1 | B | GLN | 11 | N | 3.42 | yes |  |  |
| B | GLY | 10 | N | B | GLN | 11 | N | 2.66 | yes |  |  |
| B | GLY | 10 | N | C | ASP | 17 | OD1 | 2.58 |  | yes |  |
| B | GLY | 10 | O | B | GLN | 11 | N | 2.25 | yes |  |  |
| B | GLN | 11 | O | B | ALA | 12 | N | 2.24 | yes |  |  |
| B | GLN | 11 | OE1 | B | SER | 24 | OG | 3.18 | yes |  |  |
| B | ALA | 12 | N | B | TYR | 13 | N | 3.49 | yes |  |  |
| B | ALA | 12 | O | B | TYR | 13 | N | 2.24 | yes |  |  |
| B | ALA | 12 | O | B | LEU | 23 | N | 2.64 | yes |  |  |
| B | TYR | 13 | N | C | ARG | 15 | O | 2.53 | yes |  |  |
| B | TYR | 13 | O | B | VAL | 14 | N | 2.23 | yes |  |  |
| B | TYR | 13 | O | C | ARG | 15 | N | 2.78 |  | yes |  |
| B | VAL | 14 | N | B | VAL | 21 | O | 2.48 | yes |  |  |
| B | VAL | 14 | O | B | ARG | 15 | N | 2.23 | yes |  |  |
| B | VAL | 14 | O | B | GLU | 19 | O | 3.23 | yes |  |  |
| B | VAL | 14 | O | B | VAL | 21 | N | 2.91 | yes |  |  |
| B | ARG | 15 | N | B | LYS | 16 | N | 3.27 | yes |  |  |
| B | ARG | 15 | O | B | LYS | 16 | N | 2.27 | yes |  |  |
| B | ARG | 15 | NH1 | B | LYS | 16 | N | 2.75 | yes |  |  |
| B | ARG | 15 | NH1 | B | LYS | 16 | O | 3.41 | yes |  |  |
| B | ARG | 15 | NH1 | B | ASP | 17 | N | 3.05 | yes |  |  |
| B | ARG | 15 | NH1 | B | GLY | 18 | N | 2.86 | yes |  |  |
| B | ARG | 15 | NH2 | B | GLY | 18 | N | 3.2 | yes |  |  |
| B | LYS | 16 | N | B | GLU | 19 | O | 3.04 | yes |  |  |
| B | LYS | 16 | O | B | ASP | 17 | N | 2.24 | yes |  |  |
| B | LYS | 16 | O | B | ASP | 17 | O | 3.27 | yes |  |  |
| B | LYS | 16 | O | B | ASP | 17 | OD1 | 2.19 | yes |  |  |
| B | LYS | 16 | O | B | GLY | 18 | N | 3.12 | yes |  |  |
| B | LYS | 16 | O | B | GLU | 19 | N | 2.79 | yes |  |  |
| B | LYS | 16 | O | B | GLU | 19 | O | 3.05 | yes |  |  |
| B | ASP | 17 | N | B | GLY | 18 | N | 2.86 | yes |  |  |
| B | ASP | 17 | O | B | GLY | 18 | N | 2.25 | yes |  |  |
| B | ASP | 17 | O | B | GLU | 19 | N | 3.07 | yes |  |  |
| B | GLY | 18 | N | B | GLU | 19 | N | 2.76 | yes |  |  |
| B | GLY | 18 | O | B | GLU | 19 | N | 2.25 | yes |  |  |
| B | GLU | 19 | O | B | TRP | 20 | N | 2.25 | yes |  |  |
| B | TRP | 20 | N | B | VAL | 21 | N | 3.37 | yes |  |  |
| B | TRP | 20 | O | B | VAL | 21 | N | 2.25 | yes |  |  |
| B | VAL | 21 | N | B | LEU | 22 | N | 3.46 | yes |  |  |
| B | VAL | 21 | O | B | LEU | 22 | N | 2.23 | yes |  |  |
| B | VAL | 21 | O | B | LEU | 22 | O | 3.38 | yes |  |  |
| B | VAL | 21 | O | B | LEU | 23 | N | 3.16 | yes |  |  |
| B | LEU | 22 | N | B | LEU | 23 | N | 3.45 | yes |  |  |
| B | LEU | 22 | O | B | LEU | 23 | N | 2.25 | yes |  |  |
| B | LEU | 22 | O | B | LEU | 23 | O | 3.44 | yes |  |  |
| B | LEU | 22 | O | B | SER | 24 | N | 3.11 | yes |  |  |
| B | LEU | 22 | O | B | THR | 25 | N | 2.67 | yes |  |  |
| B | LEU | 22 | O | B | THR | 25 | OG1 | 3.38 | yes |  |  |
| B | LEU | 23 | N | B | SER | 24 | N | 2.66 | yes |  |  |
| B | LEU | 23 | O | B | SER | 24 | N | 2.24 | yes |  |  |
| B | LEU | 23 | O | B | THR | 25 | N | 3.06 | yes |  |  |
| B | LEU | 23 | O | B | PHE | 26 | N | 2.73 | yes |  |  |
| B | LEU | 23 | O | B | LEU | 27 | N | 3.36 | yes |  |  |
| B | SER | 24 | N | B | THR | 25 | N | 2.68 | yes |  |  |
| B | SER | 24 | O | B | THR | 25 | N | 2.25 | yes |  |  |
| B | SER | 24 | O | B | PHE | 26 | N | 3.21 | yes |  |  |
| B | SER | 24 | O | B | LEU | 27 | N | 2.87 | yes |  |  |
| B | SER | 24 | O | B | LEU | 27 | OXT | 2.72 | yes |  |  |
| B | THR | 25 | N | B | PHE | 26 | N | 2.61 | yes |  |  |
| B | THR | 25 | O | B | PHE | 26 | N | 2.26 | yes |  |  |
| B | PHE | 26 | N | B | LEU | 27 | N | 2.57 | yes |  |  |
| B | PHE | 26 | O | B | LEU | 27 | N | 2.22 | yes |  |  |
| C | GLY | 1 | N | C | TYR | 2 | N | 2.8 | yes |  |  |
| C | GLY | 1 | O | C | TYR | 2 | N | 2.25 | yes |  |  |
| C | TYR | 2 | O | C | ILE | 3 | N | 2.26 | yes |  |  |
| C | ILE | 3 | O | C | PRO | 4 | N | 2.25 | yes |  |  |
| C | PRO | 4 | O | C | GLU | 5 | N | 2.25 | yes |  |  |
| C | PRO | 4 | O | C | GLU | 5 | O | 3.34 | yes |  |  |
| C | PRO | 4 | O | C | TRP | 20 | NE1 | 2.42 | yes |  |  |
| C | GLU | 5 | O | C | ALA | 6 | N | 2.26 | yes |  |  |
| C | ALA | 6 | N | C | TRP | 20 | NE1 | 3.41 | yes |  |  |
| C | ALA | 6 | O | C | PRO | 7 | N | 2.26 | yes |  |  |
| C | ALA | 6 | O | C | PRO | 7 | O | 3.18 | yes |  |  |
| C | ALA | 6 | O | C | ARG | 8 | NH1 | 3.23 | yes |  |  |
| C | PRO | 7 | O | C | ARG | 8 | N | 2.25 | yes |  |  |
| C | ARG | 8 | N | C | ASP | 9 | N | 2.84 | yes |  |  |
| C | ARG | 8 | O | C | ASP | 9 | N | 2.24 | yes |  |  |
| C | ASP | 9 | N | C | GLY | 10 | N | 2.68 | yes |  |  |
| C | ASP | 9 | O | C | GLY | 10 | N | 2.25 | yes |  |  |
| C | ASP | 9 | O | C | GLN | 11 | N | 3.48 | yes |  |  |
| C | ASP | 9 | OD2 | C | GLN | 11 | NE2 | 3.05 | yes |  |  |
| C | GLY | 10 | N | C | GLN | 11 | N | 2.72 | yes |  |  |
| C | GLY | 10 | O | C | GLN | 11 | N | 2.24 | yes |  |  |
| C | GLN | 11 | O | C | ALA | 12 | N | 2.25 | yes |  |  |
| C | GLN | 11 | NE2 | C | TYR | 13 | OH | 3.48 | yes |  |  |
| C | ALA | 12 | O | C | TYR | 13 | N | 2.25 | yes |  |  |
| C | ALA | 12 | O | C | LEU | 23 | N | 2.74 | yes |  |  |
| C | TYR | 13 | O | C | VAL | 14 | N | 2.24 | yes |  |  |
| C | VAL | 14 | N | C | ARG | 15 | N | 3.48 | yes |  |  |
| C | VAL | 14 | N | C | VAL | 21 | O | 2.5 | yes |  |  |
| C | VAL | 14 | O | C | ARG | 15 | N | 2.23 | yes |  |  |
| C | VAL | 14 | O | C | GLU | 19 | O | 3.18 | yes |  |  |
| C | VAL | 14 | O | C | VAL | 21 | N | 2.82 | yes |  |  |
| C | ARG | 15 | N | C | LYS | 16 | N | 3.36 | yes |  |  |
| C | ARG | 15 | O | C | LYS | 16 | N | 2.26 | yes |  |  |
| C | ARG | 15 | NE | C | GLU | 19 | N | 3.45 | yes |  |  |
| C | ARG | 15 | NH1 | C | GLY | 18 | O | 3.23 | yes |  |  |
| C | ARG | 15 | NH1 | C | TRP | 20 | NE1 | 2.99 | yes |  |  |
| C | ARG | 15 | NH2 | C | GLY | 18 | O | 2.6 | yes |  |  |
| C | LYS | 16 | N | C | ASP | 17 | N | 3.47 | yes |  |  |
| C | LYS | 16 | N | C | GLU | 19 | O | 3.02 | yes |  |  |
| C | LYS | 16 | O | C | ASP | 17 | N | 2.24 | yes |  |  |
| C | LYS | 16 | O | C | ASP | 17 | O | 3.35 | yes |  |  |
| C | LYS | 16 | O | C | GLY | 18 | N | 3.34 | yes |  |  |
| C | LYS | 16 | O | C | GLU | 19 | N | 3.05 | yes |  |  |
| C | LYS | 16 | O | C | GLU | 19 | O | 3.22 | yes |  |  |
| C | ASP | 17 | N | C | GLY | 18 | N | 2.81 | yes |  |  |
| C | ASP | 17 | O | C | GLY | 18 | N | 2.24 | yes |  |  |
| C | ASP | 17 | O | C | GLU | 19 | N | 3.03 | yes |  |  |
| C | GLY | 18 | N | C | GLU | 19 | N | 2.76 | yes |  |  |
| C | GLY | 18 | O | C | GLU | 19 | N | 2.25 | yes |  |  |
| C | GLU | 19 | O | C | TRP | 20 | N | 2.25 | yes |  |  |
| C | TRP | 20 | N | C | VAL | 21 | N | 3.37 | yes |  |  |
| C | TRP | 20 | O | C | VAL | 21 | N | 2.25 | yes |  |  |
| C | VAL | 21 | N | C | LEU | 22 | N | 3.48 | yes |  |  |
| C | VAL | 21 | O | C | LEU | 22 | N | 2.23 | yes |  |  |
| C | VAL | 21 | O | C | LEU | 22 | O | 3.41 | yes |  |  |
| C | VAL | 21 | O | C | LEU | 23 | N | 3.21 | yes |  |  |
| C | LEU | 22 | N | C | LEU | 23 | N | 3.45 | yes |  |  |
| C | LEU | 22 | O | C | LEU | 23 | N | 2.25 | yes |  |  |
| C | LEU | 22 | O | C | SER | 24 | N | 3.26 | yes |  |  |
| C | LEU | 22 | O | C | THR | 25 | N | 2.96 | yes |  |  |
| C | LEU | 22 | O | C | THR | 25 | OG1 | 3.38 | yes |  |  |
| C | LEU | 23 | N | C | SER | 24 | N | 2.78 | yes |  |  |
| C | LEU | 23 | O | C | SER | 24 | N | 2.24 | yes |  |  |
| C | LEU | 23 | O | C | THR | 25 | N | 3.08 | yes |  |  |
| C | LEU | 23 | O | C | PHE | 26 | N | 2.75 | yes |  |  |
| C | LEU | 23 | O | C | LEU | 27 | N | 3.46 | yes |  |  |
| C | SER | 24 | N | C | THR | 25 | N | 2.69 | yes |  |  |
| C | SER | 24 | O | C | THR | 25 | N | 2.25 | yes |  |  |
| C | SER | 24 | O | C | PHE | 26 | N | 3.08 | yes |  |  |
| C | SER | 24 | O | C | LEU | 27 | N | 2.86 | yes |  |  |
| C | SER | 24 | O | C | LEU | 27 | OXT | 3.48 | yes |  |  |
| C | SER | 24 | OG | C | THR | 25 | N | 3.27 | yes |  |  |
| C | THR | 25 | N | C | PHE | 26 | N | 2.59 | yes |  |  |
| C | THR | 25 | O | C | PHE | 26 | N | 2.26 | yes |  |  |
| C | PHE | 26 | N | C | LEU | 27 | N | 2.64 | yes |  |  |
| C | PHE | 26 | O | C | LEU | 27 | N | 2.23 | yes |  |  |
|  |  |  |  |  |  |  |  | **sums** | **232** | **25** | **257** |

**Table S15:** Hydrogen bonds in the foldon 1 trimer.

| Residue 1 | Atom 1 | Residue 2 | Atom 2 | Distance [Å] | intracatenane | intercatenane | total |
| --- | --- | --- | --- | --- | --- | --- | --- |
| GLY 1 (A) | O | TYR 2 (A) | N | 2.25 | yes |  |  |
| GLY 1 (A) | O | TYR 2 (A) | O | 3.4 | yes |  |  |
| GLY 1 (A) | O | GLU 5 (B) | N | 3.16 |  | yes |  |
| TYR 2 (A) | O | ILE 3 (A) | N | 2.25 | yes |  |  |
| ILE 3 (A) | N | ILE 3 (B) | O | 2.92 |  | yes |  |
| ILE 3 (A) | O | PRO 4 (A) | N | 2.25 | yes |  |  |
| ILE 3 (A) | O | ILE 3 (C) | N | 3.09 |  | yes |  |
| PRO 4 (A) | O | GLU 5 (A) | N | 2.25 | yes |  |  |
| PRO 4 (A) | O | GLU 5 (A) | O | 3.39 | yes |  |  |
| PRO 4 (A) | O | TRP 20 (A) | NE1 | 2.81 | yes |  |  |
| GLU 5 (A) | N | GLY 1 (C) | O | 3.06 |  | yes |  |
| GLU 5 (A) | O | ALA 6 (A) | N | 2.26 | yes |  |  |
| GLU 5 (A) | O | TRP 20 (A) | NE1 | 3.43 | yes |  |  |
| GLU 5 (A) | OE1 | ARG 15 (C) | NH2 | 2.82 |  | yes |  |
| GLU 5 (A) | OE2 | ALA 6 (A) | N | 2.87 | yes |  |  |
| GLU 5 (A) | OE2 | ARG 15 (C) | NE | 2.87 |  | yes |  |
| ALA 6 (A) | N | TRP 20 (A) | NE1 | 3.43 | yes |  |  |
| ALA 6 (A) | O | PRO 7 (A) | N | 2.24 | yes |  |  |
| ALA 6 (A) | O | PRO 7 (A) | O | 3 | yes |  |  |
| PRO 7 (A) | O | ARG 8 (A) | N | 2.28 | yes |  |  |
| PRO 7 (A) | O | ASP 9 (A) | N | 3.5 | yes |  |  |
| ARG 8 (A) | N | ASP 9 (A) | N | 2.72 | yes |  |  |
| ARG 8 (A) | O | ASP 9 (A) | N | 2.24 | yes |  |  |
| ASP 9 (A) | OD1 | GLN 11 (A) | N | 2.89 | yes |  |  |
| ASP 9 (A) | OD2 | TYR 13 (A) | OH | 2.43 | yes |  |  |
| GLN 11 (A) | O | ALA 12 (A) | N | 2.26 | yes |  |  |
| ALA 12 (A) | O | TYR 13 (A) | N | 2.28 | yes |  |  |
| ALA 12 (A) | O | LEU 23 (A) | N | 2.9 | yes |  |  |
| TYR 13 (A) | N | ARG 15 (C) | O | 2.83 |  | yes |  |
| TYR 13 (A) | O | VAL 14 (A) | N | 2.28 | yes |  |  |
| TYR 13 (A) | O | ARG 15 (C) | N | 3.07 |  | yes |  |
| VAL 14 (A) | N | VAL 21 (A) | O | 2.89 | yes |  |  |
| VAL 14 (A) | O | ARG 15 (A) | N | 2.25 | yes |  |  |
| VAL 14 (A) | O | VAL 21 (A) | N | 2.91 | yes |  |  |
| VAL 14 (A) | O | VAL 21 (A) | O | 3.41 | yes |  |  |
| ARG 15 (A) | N | LYS 16 (A) | N | 3.49 | yes |  |  |
| ARG 15 (A) | N | TYR 13 (B) | O | 3.06 |  | yes |  |
| ARG 15 (A) | O | LYS 16 (A) | N | 2.25 | yes |  |  |
| ARG 15 (A) | O | TYR 13 (B) | N | 2.85 |  | yes |  |
| ARG 15 (A) | NE | GLU 5 (B) | OE2 | 2.89 |  | yes |  |
| ARG 15 (A) | NH1 | TRP 20 (A) | NE1 | 3.41 | yes |  |  |
| ARG 15 (A) | NH2 | GLY 18 (A) | O | 3.21 | yes |  |  |
| ARG 15 (A) | NH2 | GLU 5 (B) | OE1 | 2.78 |  | yes |  |
| LYS 16 (A) | N | GLU 19 (A) | O | 2.91 | yes |  |  |
| LYS 16 (A) | O | GLY 18 (A) | N | 3.16 | yes |  |  |
| LYS 16 (A) | O | GLU 19 (A) | N | 3 | yes |  |  |
| LYS 16 (A) | O | GLU 19 (A) | O | 3.2 | yes |  |  |
| GLY 18 (A) | N | GLU 19 (A) | N | 2.76 | yes |  |  |
| GLY 18 (A) | O | GLU 19 (A) | N | 2.25 | yes |  |  |
| GLY 18 (A) | O | GLU 19 (A) | OE2 | 3.38 | yes |  |  |
| GLU 19 (A) | O | TRP 20 (A) | N | 2.26 | yes |  |  |
| TRP 20 (A) | N | VAL 21 (A) | N | 3.47 | yes |  |  |
| TRP 20 (A) | O | VAL 21 (A) | N | 2.26 | yes |  |  |
| VAL 21 (A) | O | LEU 22 (A) | N | 2.25 | yes |  |  |
| VAL 21 (A) | O | LEU 22 (A) | O | 3.36 | yes |  |  |
| VAL 21 (A) | O | LEU 23 (A) | N | 3.19 | yes |  |  |
| LEU 22 (A) | N | LEU 23 (A) | N | 3.49 | yes |  |  |
| LEU 22 (A) | O | LEU 23 (A) | N | 2.25 | yes |  |  |
| LEU 22 (A) | O | LEU 23 (A) | O | 3.43 | yes |  |  |
| LEU 22 (A) | O | SER 24 (A) | N | 3.23 | yes |  |  |
| LEU 22 (A) | O | THR 25 (A) | N | 3.18 | yes |  |  |
| LEU 22 (A) | O | THR 25 (A) | OG1 | 2.92 | yes |  |  |
| LEU 23 (A) | N | SER 24 (A) | N | 2.82 | yes |  |  |
| LEU 23 (A) | O | SER 24 (A) | N | 2.25 | yes |  |  |
| LEU 23 (A) | O | THR 25 (A) | N | 3.02 | yes |  |  |
| LEU 23 (A) | O | PHE 26 (A) | N | 3.01 | yes |  |  |
| SER 24 (A) | N | THR 25 (A) | N | 2.77 | yes |  |  |
| SER 24 (A) | O | THR 25 (A) | N | 2.25 | yes |  |  |
| SER 24 (A) | O | PHE 26 (A) | N | 3.16 | yes |  |  |
| SER 24 (A) | O | LEU 27 (A) | N | 3.23 | yes |  |  |
| SER 24 (A) | OG | THR 25 (A) | N | 3.26 | yes |  |  |
| THR 25 (A) | N | PHE 26 (A) | N | 2.75 | yes |  |  |
| THR 25 (A) | O | PHE 26 (A) | N | 2.26 | yes |  |  |
| THR 25 (A) | OG1 | PHE 26 (A) | N | 3.31 | yes |  |  |
| PHE 26 (A) | N | LEU 27 (A) | N | 2.7 | yes |  |  |
| PHE 26 (A) | O | LEU 27 (A) | N | 2.27 | yes |  |  |
| GLY 1 (B) | O | TYR 2 (B) | N | 2.26 | yes |  |  |
| GLY 1 (B) | O | TYR 2 (B) | O | 3.25 | yes |  |  |
| GLY 1 (B) | O | GLU 5 (C) | N | 2.97 |  | yes |  |
| TYR 2 (B) | O | ILE 3 (B) | N | 2.25 | yes |  |  |
| ILE 3 (B) | O | PRO 4 (B) | N | 2.23 | yes |  |  |
| ILE 3 (B) | O | PRO 4 (B) | O | 3.38 | yes |  |  |
| PRO 4 (B) | O | GLU 5 (B) | N | 2.25 | yes |  |  |
| PRO 4 (B) | O | GLU 5 (B) | O | 3.2 | yes |  |  |
| PRO 4 (B) | O | TRP 20 (B) | NE1 | 2.85 | yes |  |  |
| GLU 5 (B) | O | ALA 6 (B) | N | 2.26 | yes |  |  |
| GLU 5 (B) | O | ALA 6 (B) | O | 3.35 | yes |  |  |
| GLU 5 (B) | O | TRP 20 (B) | NE1 | 3.49 | yes |  |  |
| GLU 5 (B) | OE2 | ALA 6 (B) | N | 2.95 | yes |  |  |
| ALA 6 (B) | N | TRP 20 (B) | NE1 | 3.32 | yes |  |  |
| ALA 6 (B) | O | PRO 7 (B) | N | 2.25 | yes |  |  |
| ALA 6 (B) | O | PRO 7 (B) | O | 3.03 | yes |  |  |
| ALA 6 (B) | O | ARG 8 (B) | N | 3.37 | yes |  |  |
| PRO 7 (B) | O | ARG 8 (B) | N | 2.26 | yes |  |  |
| PRO 7 (B) | O | ASP 9 (B) | N | 3.47 | yes |  |  |
| ARG 8 (B) | N | ASP 9 (B) | N | 2.79 | yes |  |  |
| ARG 8 (B) | O | ASP 9 (B) | N | 2.26 | yes |  |  |
| ASP 9 (B) | OD1 | GLN 11 (B) | N | 3.17 | yes |  |  |
| ASP 9 (B) | OD1 | TYR 13 (B) | OH | 2.95 | yes |  |  |
| ASP 9 (B) | OD2 | GLN 11 (B) | OE1 | 2.73 | yes |  |  |
| ASP 9 (B) | OD2 | TYR 13 (B) | OH | 3.08 | yes |  |  |
| GLN 11 (B) | O | ALA 12 (B) | N | 2.26 | yes |  |  |
| ALA 12 (B) | O | TYR 13 (B) | N | 2.28 | yes |  |  |
| ALA 12 (B) | O | LEU 23 (B) | N | 2.98 | yes |  |  |
| TYR 13 (B) | O | VAL 14 (B) | N | 2.29 | yes |  |  |
| VAL 14 (B) | N | VAL 21 (B) | O | 2.9 | yes |  |  |
| VAL 14 (B) | O | ARG 15 (B) | N | 2.24 | yes |  |  |
| VAL 14 (B) | O | VAL 21 (B) | N | 2.92 | yes |  |  |
| VAL 14 (B) | O | VAL 21 (B) | O | 3.46 | yes |  |  |
| ARG 15 (B) | N | LYS 16 (B) | N | 3.49 | yes |  |  |
| ARG 15 (B) | N | TYR 13 (C) | O | 3.08 |  | yes |  |
| ARG 15 (B) | O | LYS 16 (B) | N | 2.25 | yes |  |  |
| ARG 15 (B) | O | TYR 13 (C) | N | 2.82 |  | yes |  |
| ARG 15 (B) | NE | GLU 5 (C) | OE2 | 2.86 |  | yes |  |
| ARG 15 (B) | NH1 | TRP 20 (B) | N | 3.48 | yes |  |  |
| ARG 15 (B) | NH1 | TRP 20 (B) | NE1 | 3.23 | yes |  |  |
| ARG 15 (B) | NH2 | GLY 18 (B) | O | 3.22 | yes |  |  |
| ARG 15 (B) | NH2 | GLU 5 (C) | OE1 | 2.77 |  | yes |  |
| LYS 16 (B) | N | GLU 19 (B) | O | 2.93 | yes |  |  |
| LYS 16 (B) | O | GLY 18 (B) | N | 3.14 | yes |  |  |
| LYS 16 (B) | O | GLU 19 (B) | N | 3 | yes |  |  |
| LYS 16 (B) | O | GLU 19 (B) | O | 3.22 | yes |  |  |
| GLY 18 (B) | N | GLU 19 (B) | N | 2.76 | yes |  |  |
| GLY 18 (B) | O | GLU 19 (B) | N | 2.26 | yes |  |  |
| GLU 19 (B) | O | TRP 20 (B) | N | 2.25 | yes |  |  |
| TRP 20 (B) | N | VAL 21 (B) | N | 3.44 | yes |  |  |
| TRP 20 (B) | O | VAL 21 (B) | N | 2.25 | yes |  |  |
| VAL 21 (B) | O | LEU 22 (B) | N | 2.23 | yes |  |  |
| VAL 21 (B) | O | LEU 22 (B) | O | 3.49 | yes |  |  |
| VAL 21 (B) | O | LEU 23 (B) | N | 3.25 | yes |  |  |
| LEU 22 (B) | N | LEU 23 (B) | N | 3.5 | yes |  |  |
| LEU 22 (B) | O | LEU 23 (B) | N | 2.23 | yes |  |  |
| LEU 22 (B) | O | LEU 23 (B) | O | 3.46 | yes |  |  |
| LEU 22 (B) | O | SER 24 (B) | N | 3.24 | yes |  |  |
| LEU 22 (B) | O | THR 25 (B) | N | 3.39 | yes |  |  |
| LEU 22 (B) | O | THR 25 (B) | OG1 | 2.85 | yes |  |  |
| LEU 23 (B) | N | SER 24 (B) | N | 2.79 | yes |  |  |
| LEU 23 (B) | O | SER 24 (B) | N | 2.25 | yes |  |  |
| LEU 23 (B) | O | THR 25 (B) | N | 3.03 | yes |  |  |
| LEU 23 (B) | O | PHE 26 (B) | N | 2.94 | yes |  |  |
| SER 24 (B) | N | THR 25 (B) | N | 2.79 | yes |  |  |
| SER 24 (B) | O | THR 25 (B) | N | 2.26 | yes |  |  |
| SER 24 (B) | O | PHE 26 (B) | N | 3.22 | yes |  |  |
| SER 24 (B) | O | LEU 27 (B) | N | 3.36 | yes |  |  |
| SER 24 (B) | OG | THR 25 (B) | N | 3.1 | yes |  |  |
| THR 25 (B) | N | PHE 26 (B) | N | 2.75 | yes |  |  |
| THR 25 (B) | O | PHE 26 (B) | N | 2.25 | yes |  |  |
| THR 25 (B) | OG1 | PHE 26 (B) | N | 3.35 | yes |  |  |
| PHE 26 (B) | N | LEU 27 (B) | N | 2.77 | yes |  |  |
| PHE 26 (B) | O | LEU 27 (B) | N | 2.24 | yes |  |  |
| PHE 26 (B) | O | LEU 27 (B) | O | 3.38 | yes |  |  |
| GLY 1 (C) | O | TYR 2 (C) | N | 2.27 | yes |  |  |
| TYR 2 (C) | O | ILE 3 (C) | N | 2.25 | yes |  |  |
| ILE 3 (C) | O | PRO 4 (C) | N | 2.25 | yes |  |  |
| PRO 4 (C) | O | GLU 5 (C) | N | 2.26 | yes |  |  |
| PRO 4 (C) | O | GLU 5 (C) | O | 3.14 | yes |  |  |
| PRO 4 (C) | O | TRP 20 (C) | NE1 | 2.84 | yes |  |  |
| GLU 5 (C) | O | ALA 6 (C) | N | 2.25 | yes |  |  |
| GLU 5 (C) | O | ALA 6 (C) | O | 3.29 | yes |  |  |
| GLU 5 (C) | OE2 | ALA 6 (C) | N | 2.93 | yes |  |  |
| ALA 6 (C) | N | TRP 20 (C) | NE1 | 3.43 | yes |  |  |
| ALA 6 (C) | O | PRO 7 (C) | N | 2.25 | yes |  |  |
| ALA 6 (C) | O | PRO 7 (C) | O | 3.02 | yes |  |  |
| PRO 7 (C) | O | ARG 8 (C) | N | 2.26 | yes |  |  |
| ARG 8 (C) | N | ASP 9 (C) | N | 2.73 | yes |  |  |
| ARG 8 (C) | O | ASP 9 (C) | N | 2.26 | yes |  |  |
| ASP 9 (C) | OD1 | GLN 11 (C) | N | 3.02 | yes |  |  |
| ASP 9 (C) | OD2 | GLN 11 (C) | NE2 | 2.99 | yes |  |  |
| ASP 9 (C) | OD2 | TYR 13 (C) | OH | 2.95 | yes |  |  |
| GLN 11 (C) | O | ALA 12 (C) | N | 2.25 | yes |  |  |
| ALA 12 (C) | O | TYR 13 (C) | N | 2.28 | yes |  |  |
| ALA 12 (C) | O | LEU 23 (C) | N | 2.92 | yes |  |  |
| TYR 13 (C) | O | VAL 14 (C) | N | 2.28 | yes |  |  |
| VAL 14 (C) | N | VAL 21 (C) | O | 2.87 | yes |  |  |
| VAL 14 (C) | O | ARG 15 (C) | N | 2.25 | yes |  |  |
| VAL 14 (C) | O | VAL 21 (C) | N | 2.91 | yes |  |  |
| VAL 14 (C) | O | VAL 21 (C) | O | 3.33 | yes |  |  |
| ARG 15 (C) | O | LYS 16 (C) | N | 2.24 | yes |  |  |
| ARG 15 (C) | NH1 | TRP 20 (C) | NE1 | 3.25 | yes |  |  |
| ARG 15 (C) | NH2 | GLY 18 (C) | O | 3.19 | yes |  |  |
| LYS 16 (C) | N | GLU 19 (C) | O | 2.89 | yes |  |  |
| LYS 16 (C) | O | GLY 18 (C) | N | 3.21 | yes |  |  |
| LYS 16 (C) | O | GLU 19 (C) | N | 3.08 | yes |  |  |
| LYS 16 (C) | O | GLU 19 (C) | O | 3.18 | yes |  |  |
| GLY 18 (C) | N | GLU 19 (C) | N | 2.77 | yes |  |  |
| GLY 18 (C) | O | GLU 19 (C) | N | 2.25 | yes |  |  |
| GLY 18 (C) | O | GLU 19 (C) | OE1 | 3.45 | yes |  |  |
| GLY 18 (C) | O | GLU 19 (C) | OE2 | 3.35 | yes |  |  |
| GLU 19 (C) | O | TRP 20 (C) | N | 2.25 | yes |  |  |
| TRP 20 (C) | N | VAL 21 (C) | N | 3.49 | yes |  |  |
| TRP 20 (C) | O | VAL 21 (C) | N | 2.27 | yes |  |  |
| VAL 21 (C) | O | LEU 22 (C) | N | 2.23 | yes |  |  |
| VAL 21 (C) | O | LEU 23 (C) | N | 3.37 | yes |  |  |
| LEU 22 (C) | O | LEU 23 (C) | N | 2.24 | yes |  |  |
| LEU 22 (C) | O | SER 24 (C) | N | 3.27 | yes |  |  |
| LEU 22 (C) | O | THR 25 (C) | N | 3.22 | yes |  |  |
| LEU 22 (C) | O | THR 25 (C) | OG1 | 2.64 | yes |  |  |
| LEU 23 (C) | N | SER 24 (C) | N | 2.79 | yes |  |  |
| LEU 23 (C) | O | SER 24 (C) | N | 2.27 | yes |  |  |
| LEU 23 (C) | O | THR 25 (C) | N | 3 | yes |  |  |
| LEU 23 (C) | O | PHE 26 (C) | N | 2.93 | yes |  |  |
| SER 24 (C) | N | THR 25 (C) | N | 2.75 | yes |  |  |
| SER 24 (C) | O | THR 25 (C) | N | 2.25 | yes |  |  |
| SER 24 (C) | O | THR 25 (C) | O | 3.49 | yes |  |  |
| SER 24 (C) | O | PHE 26 (C) | N | 3.07 | yes |  |  |
| SER 24 (C) | O | LEU 27 (C) | N | 3.32 | yes |  |  |
| SER 24 (C) | OG | THR 25 (C) | N | 3.29 | yes |  |  |
| THR 25 (C) | N | PHE 26 (C) | N | 2.76 | yes |  |  |
| THR 25 (C) | O | PHE 26 (C) | N | 2.25 | yes |  |  |
| PHE 26 (C) | N | LEU 27 (C) | N | 2.75 | yes |  |  |
| PHE 26 (C) | O | LEU 27 (C) | N | 2.24 | yes |  |  |
|  |  |  |  | **sums** | **194** | **17** | **211** |

**Table S16:** Salt bridges in the foldon 0 trimer.

| chain1 | residue1 | res_id1 | atom1 | chain2 | residue2 | res_id2 | atom2 | Distance [Å] | intracatenane | intercatenane | total |
| --- | --- | --- | --- | --- | --- | --- | --- | --- | --- | --- | --- |
| A | GLU | 5 | OE2 | B | ARG | 15 | NE | 2.85 |  | yes |  |
| A | GLU | 5 | OE2 | B | ARG | 15 | NH2 | 2.29 |  | yes |  |
| A | ARG | 15 | NE | A | GLU | 19 | O | 3.82 | yes |  |  |
| A | ARG | 15 | NE | C | GLU | 5 | OE2 | 2.97 |  | yes |  |
| A | ARG | 15 | NH1 | A | GLU | 19 | O | 3.94 | yes |  |  |
| A | ARG | 15 | NH2 | C | GLU | 5 | OE1 | 3.58 |  | yes |  |
| A | ARG | 15 | NH2 | C | GLU | 5 | OE2 | 2.26 |  | yes |  |
| A | LYS | 16 | N | A | GLU | 19 | O | 3.16 | yes |  |  |
| B | GLU | 5 | OE1 | B | ARG | 8 | NE | 3.39 | yes |  |  |
| B | GLU | 5 | OE1 | B | ARG | 8 | NH2 | 2.64 | yes |  |  |
| B | GLU | 5 | OE1 | C | ARG | 15 | NH2 | 3.54 |  | yes |  |
| B | GLU | 5 | OE2 | B | ARG | 8 | NE | 3.42 | yes |  |  |
| B | GLU | 5 | OE2 | B | ARG | 8 | NH2 | 3.66 | yes |  |  |
| B | GLU | 5 | OE2 | C | ARG | 15 | NE | 2.96 |  | yes |  |
| B | GLU | 5 | OE2 | C | ARG | 15 | NH2 | 2.28 |  | yes |  |
| B | LYS | 16 | N | B | GLU | 19 | O | 3.04 | yes |  |  |
| C | ARG | 15 | NE | C | GLU | 19 | O | 3.82 | yes |  |  |
| C | LYS | 16 | N | C | GLU | 19 | O | 3.02 | yes |  |  |
|  |  |  |  |  |  |  |  | **sums** | **10** | **8** | **18** |

**Table S17:** Salt bridges in the foldon 1 trimer.

| Residue 1 | Atom 1 | Residue 2 | Atom 2 | Distance [Å] | intracatenane | intercatenane | total |
| --- | --- | --- | --- | --- | --- | --- | --- |
| GLU 5 (A) | OE1 | ARG 15 (C) | NE | 3.7 |  | yes |  |
| GLU 5 (A) | OE1 | ARG 15 (C) | NH2 | 2.82 |  | yes |  |
| GLU 5 (A) | OE2 | ARG 15 (C) | NE | 2.87 |  | yes |  |
| GLU 5 (A) | OE2 | ARG 15 (C) | NH2 | 3.54 |  | yes |  |
| ARG 15 (A) | NE | GLU 19 (A) | O | 3.95 | yes |  |  |
| ARG 15 (A) | NE | GLU 5 (B) | OE1 | 3.59 |  | yes |  |
| ARG 15 (A) | NE | GLU 5 (B) | OE2 | 2.89 |  | yes |  |
| ARG 15 (A) | NH1 | GLU 19 (A) | O | 3.87 | yes |  |  |
| ARG 15 (A) | NH2 | GLU 5 (B) | OE1 | 2.78 |  | yes |  |
| ARG 15 (A) | NH2 | GLU 5 (B) | OE2 | 3.62 |  | yes |  |
| LYS 16 (A) | N | GLU 19 (A) | O | 2.91 | yes |  |  |
| ARG 15 (B) | NE | GLU 19 (B) | O | 3.94 | yes |  |  |
| ARG 15 (B) | NE | GLU 5 (C) | OE1 | 3.54 |  | yes |  |
| ARG 15 (B) | NE | GLU 5 (C) | OE2 | 2.86 |  | yes |  |
| ARG 15 (B) | NH1 | GLU 19 (B) | O | 3.64 | yes |  |  |
| ARG 15 (B) | NH2 | GLU 5 (C) | OE1 | 2.77 |  | yes |  |
| ARG 15 (B) | NH2 | GLU 5 (C) | OE2 | 3.64 |  | yes |  |
| LYS 16 (B) | N | GLU 19 (B) | O | 2.93 | yes |  |  |
| ARG 15 (C) | NE | GLU 19 (C) | O | 3.95 | yes |  |  |
| ARG 15 (C) | NH1 | GLU 19 (C) | O | 3.88 | yes |  |  |
| LYS 16 (C) | N | GLU 19 (C) | O | 2.89 | yes |  |  |
|  |  |  |  | **sums** | **9** | **12** | **21** |

**Table S18:** Drift times, experimentally determined CCS, and literature references of calibrant proteins.

| **protein**  **name** | **species** | **CCS determinations** ^a)^ | | | |  | **CCS from literature** ^a)^ | | | **reference** |
| --- | --- | --- | --- | --- | --- | --- | --- | --- | --- | --- |
|  |  | ***m/z*** | **z** | **dt exp.** | **^TW^CCS_N2_** |  | ***m/z*** | **z** | **CCS exp.** |  |
| serum | Bos | 4432.04 | 15 | 18.80 | 4310 |  | 4600.07 | 15 | 4490 | ^66^ |
| albumin | taurus | 4432.59 | 15 | 18.80 | 4310 |  | / | / | / |  |
|  |  | 4155.21 | 16 | 17.56 | 4460 |  | 4312.56 | 16 | 4470 | ^66^ |
|  |  | 4155.07 | 16 | 17.42 | 4445 |  | / | / | / |  |
|  |  | 3910.85 | 17 | 17.28 | 4706 |  | 4058.88 | 17 | 4490 | ^66^ |
|  |  | 3911.10 | 17 | 17.56 | 4740 |  | / | / | / |  |
| myoglobin | Equus | 2196.44 | 8 | 11.06 | 1872 |  | 2196.90 | 8 | 1937 | ^67^ |
| (holo) | caballus | 2196.42 | 8 | 11.20 | 1879 |  | / | / | / |  |
|  |  | 1952.60 | 9 | 10.09 | 2045 |  | 1952.90 | 9 | 2085 | ^67^ |
|  |  | 1952.47 | 9 | 10.23 | 2054 |  | / | / | / |  |
| cytochrome c | Equus | 2473.12 | 5 | 12.03 | 1204 |  | 2472.80 | 5 | 1146 | ^67^ |
|  | caballus | 2473.14 | 5 | 11.89 | 1199 |  | / | / | / |  |
|  |  | 2060.97 | 6 | 10.37 | 1376 |  | 2060.90 | 6 | 1477/1490 | ^66,67^ |
|  |  | 2061.15 | 6 | 10.51 | 1381 |  | / | / | / |  |
|  |  | 1766.45 | 7 | 11.20 | 1645 |  | 1714.43 | 7 | 1590 | ^66^ |
|  |  | 1766.58 | 7 | 11.34 | 1652 |  | / | / | / |  |
|  |  | 1545.66 | 8 | 10.23 | 1827 |  | / | / | / |  |
|  |  | 1545.66 | 8 | 10.37 | 1835 |  | / | / | / |  |
| ubiquitin | Bos | 2142.17 | 4 | 11.75 | 956 |  | 2141.00 | 4 | 949 | ^67^ |
|  | taurus | 2142.21 | 4 | 11.75 | 956 |  | / | / | / |  |
|  |  | 1713.93 | 5 | 9.95 | 1132 |  | 1713.00 | 5 | 1011 | ^67^ |
|  |  | 1713.97 | 5 | 10.09 | 1137 |  | / | / | / |  |
|  |  | 1428.45 | 6 | 8.85 | 1313 |  | / | / | / |  |
|  |  | 1428.45 | 6 | 11.2 | 1411 |  | / | / | / |  |
|  |  | 1428.46 | 6 | 8.99 | 1319 |  | / | / | / |  |
|  |  | 1428.45 | 6 | 11.47 | 1423 |  | / | / | / |  |
| insulin | Bos | 1912.25 | 3 | 12.3 | 729 |  | 1912.25 | 3 | 729 | ^b)^ |
|  | taurus | 1912.25 | 3 | 12.44 | 732 |  | 1912.25 | 3 | 732 |  |
|  |  | 1434.19 | 4 | 8.43 | 865 |  | 1434.19 | 4 | 865 |  |
|  |  | 1434.19 | 4 | 8.57 | 868 |  | 1434.19 | 4 | 868 |  |
|  |  | 1147.55 | 5 | 7.19 | 1038 |  | 1147.55 | 5 | 1038 |  |
|  |  | 1147.55 | 5 | 7.19 | 1038 |  | 1147.55 | 5 | 1038 |  |

a) drift gas: nitrogen

b) this work

Table S19: Drift times of Foldon0 trimer, dimer, and monomer ion signals at different collision cell voltage differences ^a)^.

| ΔCV | trimer ^b)^ | | | |  | dimer ^b)^ | | | |  | monomer ^b)^ | | | |
| --- | --- | --- | --- | --- | --- | --- | --- | --- | --- | --- | --- | --- | --- | --- |
|  | *m/z* | *z* | dt [ms] | FWHM |  | *m/z* | *z* | dt [ms] | FWHM |  | *m/z* | *z* | dt [ms] | FWHM |
| 2 V | 1849.78 | 5+ | 10.7 | 1.9 |  | n.o. | - | - | - |  | n.o. | - | - | - |
| 5 V | 1849.78 | 5+ | 10.8 | 1.7 |  | n.o. | - | - | - |  | n.o. | - | - | - |
| 10 V | 1849.78 | 5+ | 10.8 | 1.3 |  | 2055.43 | 3+ | 13.3 | 0.9 |  | 1541.30 | 2+ | 12.4 | 1.0 |
| 15 V | 1849.78 | 5+ | 10.9 | 1.1 |  | 2055.09 | 3+ | 13.3 | 0.9 |  | 1541.82 | 2+ | 12.4 | 0.9 |
| 20 V | 1849.78 | 5+ | 11.1 | 0.9 |  | 2055.76 | 3+ | 13.3 | 0.9 |  | 1541.30 | 2+ | 12.4 | 1.0 |
| 25 V | n.o. | - | - | - |  | 2055.07 | 3+ | 13.3 | 0.8 |  | 1541.30 | 2+ | 12.4 | 1.0 |
| 30 V | n.o. | - | - | - |  | 2054.73 | 3+ | 13.3 | 0.9 |  | 1541.30 | 2+ | 12.4 | 1.0 |
| 35 V | n.o. | - | - | - |  | 2054.77 | 3+ | 13.3 | 0.9 |  | 1541.32 | 2+ | 12.4 | 1.0 |
| 40 V | n.o. | - | - | - |  | 2055.74 | 3+ | 13.3 | 0.9 |  | 1541.30 | 2+ | 12.6 | 1.0 |

1. First measurement series b) n.o.: not observed

Table S20: Drift times of Foldon0 trimer, dimer, and monomer ion signals at different collision voltage differences ^a)^.

| ΔCV | trimer ^b)^ | | | |  | dimer ^b)^ | | | |  | monomer ^b)^ | | | |
| --- | --- | --- | --- | --- | --- | --- | --- | --- | --- | --- | --- | --- | --- | --- |
|  | *m/z* | *z* | dt [ms] | FWHM |  | *m/z* | z | dt [ms] | FWHM |  | *m/z* | z | dt [ms] | FWHM |
| 2 V | 1849.78 | 5+ | 10.8 | 0.4 |  | n.o. | - | - | - |  | n.o. | - | - | - |
| 5 V | 1849.78 | 5+ | 10.8 | 1.7 |  | n.o. | - | - | - |  | n.o. | - | - | - |
| 10 V | 1849.80 | 5+ | 11.1 | 1.1 |  | 2055.41 | 3+ | 13.6 | 0.8 |  | 1540.83 | 2+ | 12.6 | 1.0 |
| 15 V | 1850.01 | 5+ | 11.1 | 0.1 |  | 2055.09 | 3+ | 13.3 | 0.7 |  | 1541.32 | 2+ | 12.6 | 0.9 |
| 20 V | 1849.56 | 5+ | 10.9 | 0.9 |  | 2055.43 | 3+ | 13.3 | 0.9 |  | 1541.84 | 2+ | 12.4 | 1.0 |
| 25 V | n.o. | - | - | - |  | 2057.77 | 3+ | 13.3 | 0.8 |  | 1541.32 | 2+ | 12.6 | 0.9 |
| 30 V | n.o. | - | - | - |  | 2054.42 | 3+ | 13.3 | 0.8 |  | 1541.32 | 2+ | 12.6 | 1.0 |
| 35 V | n.o. | - | - | - |  | 2054.77 | 3+ | 13.3 | 0.8 |  | 1541.32 | 2+ | 12.2 | 1.0 |
| 40 V | n.o. | - | - | - |  | 2056.12 | 3+ | 13.6 | 0.8 |  | 1541.30 | 2+ | 12.4 | 1.0 |

1. Second measurement series b) n.o.: not observed

Table S21: Drift times of Foldon1 trimer, dimer, and monomer ion signals at different collision cell voltage differences ^a)^.

| ΔCV | trimer ^b)^ | | | |  | dimer ^b)^ | | | |  | monomer ^b)^ | | | |
| --- | --- | --- | --- | --- | --- | --- | --- | --- | --- | --- | --- | --- | --- | --- |
|  | m/z | z | dt [ms] | FWHM |  | m/z | z | dt [ms] | FWHM |  | m/z | z | dt [ms] | FWHM |
| 2 V | 1876.85 | 5+ | 11.1 | 1.5 |  | n.o. | - | - | - |  | n.o. | - | - | - |
| 5 V | 1877.05 | 5+ | 11.1 | 0.9 |  | 2085.54 | 3+ | 13.8 | 0.8 |  | 1564.36 | 2+ | 12.4 | 0.6 |
| 10 V | 1877.05 | 5+ | 11.1 | 0.7 |  | 2085.48 | 3+ | 13.6 | 0.8 |  | 1563.88 | 2+ | 12.4 | 0.6 |
| 15 V | 1877.05 | 5+ | 11.1 | 0.7 |  | 2085.52 | 3+ | 13.7 | 0.9 |  | 1564.38 | 2+ | 12.4 | 0.7 |
| 20 V | 1877.26 | 5+ | 11.1 | 0.8 |  | 2085.18 | 3+ | 13.6 | 0.9 |  | 1563.88 | 2+ | 12.4 | 0.7 |
| 25 V | n.o. | - | - | - |  | 2085.52 | 3+ | 13.7 | 0.9 |  | 1563.88 | 2+ | 12.4 | 0.7 |
| 30 V | n.o. | - | - | - |  | 2085.52 | 3+ | 13.7 | 0.9 |  | 1563.88 | 2+ | 12.4 | 0.6 |
| 35 V | n.o. | - | - | - |  | 2085.50 | 3+ | 13.7 | 0.8 |  | 1563.88 | 2+ | 12.4 | 0.6 |
| 40 V | n.o. | - | - | - |  | n.o. | - | - | - |  | 1563.88 | 2+ | 12.4 | 0.7 |

1. First measurement series b) n.o.: not observed

Table S22: Drift times of Foldon1 trimer, dimer, and monomer ion signals at different collision voltage differences ^a)^.

| ΔCV | trimer ^b)^ | | | |  | dimer ^b)^ | | | |  | monomer ^b)^ | | | |
| --- | --- | --- | --- | --- | --- | --- | --- | --- | --- | --- | --- | --- | --- | --- |
|  | m/z | z | dt [ms] | FWHM |  | m/z | z | dt [ms] | FWHM |  | m/z | z | dt [ms] | FWHM |
| 2 V | 1877.05 | 5+ | 11.1 | 0.8 |  | n.o. | - | - | - |  | n.o. | - | - | - |
| 5 V | 1876.86 | 5+ | 11.1 | 0.7 |  | 2085.50 | 3+ | 13.7 | 0.7 |  | 1564.38 | 2+ | 12.6 | 0.7 |
| 10 V | 1876.86 | 5+ | 11.1 | 0.7 |  | 2085.50 | 3+ | 13.7 | 0.9 |  | 1563.90 | 2+ | 12.6 | 0.0 |
| 15V | 1876. | 5+ | 11.2 | 0.7 |  | 2085.18 | 3+ | 13.7 | 0.8 |  | 1563.88 | 2+ | 12.4 | 0.5 |
| 20 V | n.o. | - | - | - |  | 2085.16 | 3+ | 13.7 | 0.9 |  | 1563.88 | 2+ | 12.4 | 0.7 |
| 25 V | n.o. | - | - | - |  | 2085.52 | 3+ | 13.7 | 0.9 |  | 1564.38 | 2+ | 12.4 | 0.7 |
| 30 V | n.o. | - | - | - |  | 2085.50 | 3+ | 13.7 | 0.9 |  | 1564.40 | 2+ | 12.4 | 0.7 |
| 35 V | n.o. | - | - | - |  | 2084.84 | 3+ | 13.7 | 0.9 |  | 1563.90 | 2+ | 12.4 | 0.7 |
| 40 V | n.o. | - | - | - |  | n.o. | - | - | - |  | 1563.88 | 2+ | 12.4 | 0.7 |

1. Second measurement series b) n.o.: not observed

Table S23: Drift times of Foldon2 trimer, dimer, and monomer ion signals at different collision cell voltage differences ^a)^.

| ΔCV | trimer ^b)^ | | | |  | dimer ^b)^ | | | |  | monomer ^b)^ | | | |
| --- | --- | --- | --- | --- | --- | --- | --- | --- | --- | --- | --- | --- | --- | --- |
|  | m/z | z | dt [ms] | FWHM |  | m/z | z | dt [ms] | FWHM |  | m/z | z | dt [ms] | FWHM |
| 2 V | 1877.05 | 5+ | 10.9 | 1.4 |  | n.o. | - | - | - |  | n.o. | - | - | - |
| 5 V | 1877.05 | 5+ | 10.9 | 1.3 |  | 2085.14 | 3+ | 13.6 | 0.9 |  | 1563.86 | 2+ | 12.3 | 0.7 |
| 10 V | 1876.66 | 5+ | 11.0 | 0.8 |  | 2085.18 | 3+ | 13.6 | 0.9 |  | 1564.40 | 2+ | 12.3 | 0.8 |
| 15 V | 1876.86 | 5+ | 11.0 | 0.8 |  | 2085.18 | 3+ | 13.6 | 0.9 |  | 1563.88 | 2+ | 12.3 | 0.7 |
| 20 V | 1876.86 | 5+ | 11.0 | 0.8 |  | 2085.52 | 3+ | 13.6 | 0.9 |  | 1563.88 | 2+ | 12.3 | 0.7 |
| 25 V | n.o. | - | - | - |  | 2085.16 | 3+ | 13.6 | 0.9 |  | 1563.88 | 2+ | 12.3 | 0.8 |
| 30 V | n.o. | - | - | - |  | 2085.16 | 3+ | 13.7 | 0.9 |  | 1563.88 | 2+ | 12.3 | 0.7 |
| 35 V | n.o. | - | - | - |  | 2085.50 | 3+ | 13.7 | 0.9 |  | 1563.88 | 2+ | 12.3 | 0.7 |
| 40 V | n.o. | - | - | - |  | n.o. | - | - | - |  | 1563.88 | 2+ | 12.3 | 0.8 |

1. First measurement series b) n.o. not observed

Table S24: Drift times of Foldon2 trimer, dimer, and monomer ion signals at different collision voltage differences ^a)^.

| ΔCV | trimer ^b)^ | | | |  | dimer ^b)^ | | | |  | monomer ^b)^ | | | |
| --- | --- | --- | --- | --- | --- | --- | --- | --- | --- | --- | --- | --- | --- | --- |
|  | m/z | z | dt [ms] | FWHM |  | m/z | z | dt [ms] | FWHM |  | m/z | z | dt [ms] | FWHM |
| 2 V | 1876.85 | 5+ | 11.0 | 1.5 |  | n.o. | - | - | - |  | n.o. | - | - | - |
| 5 V | 1876.86 | 5+ | 11.0 | 1.2 |  | 2085.52 | 3+ | 13.7 | 0.9 |  | 1564.38 | 2+ | 12.3 | 0.7 |
| 10 V | 1876.86 | 5+ | 11.0 | 0.8 |  | 2085.16 | 3+ | 13.7 | 0.9 |  | 1563.88 | 2+ | 12.4 | 0.7 |
| 15V | 1876.86 | 5+ | 11.0 | 0.8 |  | 2085.18 | 3+ | 13.7 | 0.9 |  | 1563.88 | 2+ | 12.4 | 0.7 |
| 20 V | n.o. | - | - | - |  | 2085.18 | 3+ | 13.7 | 1.0 |  | 1563.88 | 2+ | 12.3 | 0.8 |
| 25 V | n.o. | - | - | - |  | 2085.18 | 3+ | 13.7 | 1.0 |  | 1563.88 | 2+ | 12.3 | 0.8 |
| 30 V | n.o. | - | - | - |  | 2085.18 | 3+ | 13.7 | 1.0 |  | 1563.88 | 2+ | 12.3 | 0.9 |
| 35 V | n.o. | - | - | - |  | 2085.18 | 3+ | 13.7 | 0.9 |  | 1563.88 | 2+ | 12.3 | 0.9 |
| 40 V | n.o. | - | - | - |  | n.o. | - | - | - |  | 1564.40 | 2+ | 12.3 | 0.9 |

1. Second measurement series b) n.o. not observed

Table S25: Drift times of Foldon3 trimer, dimer, and monomer ion signals at different collision cell voltage differences ^a)^.

| ΔCV | trimer ^b)^ | | | |  | dimer ^b)^ | | | |  | monomer ^b)^ | | | |
| --- | --- | --- | --- | --- | --- | --- | --- | --- | --- | --- | --- | --- | --- | --- |
|  | m/z | z | dt [ms] | FWHM |  | m/z | z | dt [ms] | FWHM |  | m/z | z | dt [ms] | FWHM |
| 2 V | 1842.83 | 5+ | 10.8 | 1.2 |  | n.o. | - | - | - |  | n.o. | - | - | - |
| 5 V | 1842.65 | 5+ | 10.8 | 1.1 |  | 2047.15 | 3+ | 13.7 | 1.0 |  | 1535.87 | 2+ | 12.2 | 0.6 |
| 10 V | 1842.65 | 5+ | 10.9 | 1.0 |  | 2047.49 | 3+ | 13.7 | 1.0 |  | 1535.37 | 2+ | 12.3 | 0.6 |
| 15 V | 1842.65 | 5+ | 10.9 | 1.0 |  | 2047.14 | 3+ | 13.7 | 1.0 |  | 1535.37 | 2+ | 12.2 | 0.6 |
| 20 V | n.o. | - | - |  |  | 2047.15 | 3+ | 13.7 | 1.1 |  | 1535.37 | 2+ | 12.2 | 0.7 |
| 25 V | n.o. | - | - | - |  | 2047.15 | 3+ | 13.7 | 1.0 |  | 1535.37 | 2+ | 12.3 | 0.7 |
| 30 V | n.o. | - | - | - |  | 2047.15 | 3+ | 13.7 | 1.0 |  | 1535.37 | 2+ | 12.3 | 0.7 |
| 35 V | n.o. | - | - | - |  | 2047.47 | 3+ | 13.7 | 1.0 |  | 1535.37 | 2+ | 12.3 | 0.7 |
| 40 V | n.o. | - | - | - |  | n.o. | - | - | - |  | 1535.37 | 2+ | 12.3 | 0.8 |

1. First measurement series b) n.o. not observed

Table S26: Drift times of Foldon3 trimer, dimer, and monomer ion signals at different collision voltage differences ^a)^.

| ΔCV | trimer ^b)^ | | | |  | dimer ^b)^ | | | |  | monomer ^b)^ | | | |
| --- | --- | --- | --- | --- | --- | --- | --- | --- | --- | --- | --- | --- | --- | --- |
|  | m/z | z | dt [ms] | FWHM |  | m/z | z | dt [ms] | FWHM |  | m/z | z | dt [ms] | FWHM |
| 2 V | 1842.65 | 5+ | 10.9 | 1.0 |  | n.o. | - | - | - |  | n.o. | - | - | - |
| 5 V | 1842.65 | 5+ | 10.9 | 1.1 |  | 2047.51 | 3+ | 13.8 | 1.0 |  | 1535.35 | 2+ | 12.4 | 0.7 |
| 10 V | 1842.65 | 5+ | 10.9 | 1.1 |  | 2047.51 | 3+ | 13.8 | 1.0 |  | 1535.37 | 2+ | 12.3 | 0.7 |
| 15 V | 1842.65 | 5+ | 10.9 | 1.0 |  | 2047.17 | 3+ | 13.7 | 1.1 |  | 1535.37 | 2+ | 12.3 | 0.7 |
| 20 V | n.o. | - | - | - |  | 2047.17 | 3+ | 13.7 | 1.2 |  | 1535.37 | 2+ | 12.3 | 0.7 |
| 25 V | n.o. | - | - | - |  | 2047.17 | 3+ | 13.8 | 1.1 |  | 1535.37 | 2+ | 12.3 | 0.7 |
| 30 V | n.o. | - | - | - |  | 2047.17 | 3+ | 13.8 | 1.1 |  | 1535.37 | 2+ | 12.3 | 0.7 |
| 35 V | n.o. | - | - | - |  | 2047.51 | 3+ | 13.8 | 1.1 |  | 1535.37 | 2+ | 12.3 | 0.8 |
| 40 V | n.o. | - | - | - |  | n.o. | - | - | - |  | 1535.37 | 2+ | 12.3 | 0.8 |

1. Second measurement series b) n.o. not observed

Table S27: Drift times of Foldon4 trimer, dimer, and monomer ion signals at different collision cell voltage differences ^a)^.

| ΔCV | trimer ^b)^ | | | |  | dimer ^b)^ | | | |  | monomer ^b)^ | | | |
| --- | --- | --- | --- | --- | --- | --- | --- | --- | --- | --- | --- | --- | --- | --- |
|  | m/z | z | dt [ms] | FWHM |  | m/z | z | dt [ms] | FWHM |  | m/z | z | dt [ms] | FWHM |
| 2 V | 1842.81 | 5+ | 10.8 | 1.5 |  | n.o. | - | - | - |  | n.o. | - | - | - |
| 5 V | 1842.81 | 5+ | 10.8 | 1.3 |  | n.o. | - | - | - |  | n.o. | - | - | - |
| 10 V | 1842.63 | 5+ | 10.8 | 1.1 |  | 2046.80 | 3+ | 13.3 | 0.9 |  | 1535.35 | 2+ | 12.2 | 0.6 |
| 15 V | 1842.81 | 5+ | 10.8 | 1.1 |  | 2047.15 | 3+ | 13.4 | 0.9 |  | 1535.35 | 2+ | 12.3 | 0.6 |
| 20 V | 1842.61 | 5+ | 10.8 | 1.1 |  | 2047.13 | 3+ | 13.4 | 0.9 |  | 1535.35 | 2+ | 12.3 | 0.6 |
| 25 V | n.o. | - | - | - |  | 2047.47 | 3+ | 13.4 | 0.9 |  | 1535.35 | 2+ | 12.3 | 0.7 |
| 30 V | n.o. | - | - | - |  | 2047.47 | 3+ | 13.6 | 0.9 |  | 1535.35 | 2+ | 12.3 | 0.7 |
| 35 V | n.o. | - | - | - |  | 2047.13 | 3+ | 13.6 | 0.9 |  | 1535.35 | 2+ | 12.3 | 0.7 |
| 40 V | n.o. | - | - | - |  | n.o. | - | - | - |  | 1535.35 | 2+ | 12.3 | 0.7 |

1. First measurement series b) n.o. not observed

Table S28: Drift times of Foldon3 trimer, dimer, and monomer ion signals at different collision voltage differences ^a)^.

| ΔCV | trimer ^b)^ | | | |  | dimer ^b)^ | | | |  | monomer ^b)^ | | | |
| --- | --- | --- | --- | --- | --- | --- | --- | --- | --- | --- | --- | --- | --- | --- |
|  | m/z | z | dt [ms] | FWHM |  | m/z | z | dt [ms] | FWHM |  | m/z | z | dt [ms] | FWHM |
| 2 V | 1842.63 | 5+ | 10.9 | 1.6 |  | n.o. | - | - | - |  | n.o. | - | - | - |
| 5 V | 1842.65 | 5+ | 10.9 | 1.3 |  | n.o. | - | - | - |  | n.o. | - | - | - |
| 10 V | 1842.65 | 5+ | 10.9 | 1.1 |  | 2047.49 | 3+ | 13.6 | 0.9 |  | 1535.37 | 2+ | 12.3 | 0.7 |
| 15 V | 1842.85 | 5+ | 10.9 | 1.1 |  | 2047.49 | 3+ | 13.6 | 0.9 |  | 1535.37 | 2+ | 12.3 | 0.6 |
| 20 V | 1842.83 | 5+ | 10.9 | 1.2 |  | 2047.49 | 3+ | 13.6 | 0.9 |  | 1535.35 | 2+ | 12.3 | 0.6 |
| 25 V | n.o. | - | - | - |  | 2047.15 | 3+ | 13.6 | 0.9 |  | 1535.37 | 2+ | 12.3 | 0.6 |
| 30 V | n.o. | - | - | - |  | 2047.15 | 3+ | 13.6 | 1.0 |  | 1535.37 | 2+ | 12.3 | 0.6 |
| 35 V | n.o. | - | - | - |  | 2047.15 | 3+ | 13.6 | 0.9 |  | 1535.37 | 2+ | 12.3 | 0.7 |
| 40 V | n.o. | - | - | - |  | n.o. | - | - | - |  | 1535.87 | 2+ | 12.3 | 0.7 |

1. Second measurement series b) n.o. not observed

Table S29: Drift times of Foldon5 trimer. dimer. and monomer ion signals at different collision cell voltage differences ^a)^.

| ΔCV | trimer ^b)^ | | | |  | dimer ^b)^ | | | |  | monomer ^b)^ | | | |
| --- | --- | --- | --- | --- | --- | --- | --- | --- | --- | --- | --- | --- | --- | --- |
|  | m/z | z | dt [ms] | FWHM |  | m/z | z | dt [ms] | FWHM |  | m/z | z | dt [ms] | FWHM |
| 2 V | 1863.27 | 5+ | 10.9 | 0.8 |  | n.o. | - | - | - |  | n.o. | - | - | - |
| 5 V | 1863.06 | 5+ | 11.1 | 1.0 |  | 2069.50 | 3+ | 13.8 | 1.0 |  | 1552.88 | 2+ | 12.4 | 0.6 |
| 10 V | 1863.08 | 5+ | 11.2 | 1.0 |  | 2070.18 | 3+ | 13.8 | 1.0 |  | 1552.4 | 2+ | 12.4 | 0.7 |
| 15 V | 1863.06 | 5+ | 11.2 | 1.1 |  | 2070.53 | 3+ | 13.7 | 1.0 |  | 1552.40 | 2+ | 12.6 | 0.6 |
| 20 V | 1863.08 | 5+ | 11.2 | 1.0 |  | 2070.18 | 3+ | 13.7 | 1.0 |  | 1552.38 | 2+ | 12.6 | 0.7 |
| 25 V | n.o. | - | - | - |  | 2070.18 | 3+ | 13.7 | 1.0 |  | 1552.40 | 2+ | 12.6 | 0.6 |
| 30 V | n.o. | - | - | - |  | 2069.54 | 3+ | 13.8 | 1.1 |  | 1552.38 | 2+ | 12.6 | 0.7 |
| 35 V | n.o. | - | - | - |  | 2070.18 | 3+ | 13.7 | 0.7 |  | 1552.38 | 2+ | 12.6 | 0.6 |
| 40 V | n.o. | - | - | - |  | n.o. | - | - | - |  | 1552.40 | 2+ | 12.6 | 0.7 |

1. First measurement series b) n.o. not observed

Table S30: Drift times of Foldon5 trimer. dimer. and monomer ion signals at different collision voltage differences ^a)^.

| ΔCV | trimer ^b)^ | | | |  | dimer ^b)^ | | | |  | monomer ^b)^ | | | |
| --- | --- | --- | --- | --- | --- | --- | --- | --- | --- | --- | --- | --- | --- | --- |
|  | m/z | z | dt [ms] | FWHM |  | m/z | z | dt [ms] | FWHM |  | m/z | z | dt [ms] | FWHM |
| 2 V | 1863.08 | 5+ | 11.1 | 0.8 |  | n.o. | - | - | - |  | n.o. | - | - | - |
| 5 V | 1862.88 | 5+ | 11.2 | 1.0 |  | 2070.20 | 3+ | 13.7 | 1.0 |  | 1553.41 | 2+ | 12.4 | 0.7 |
| 10 V | 1863.06 | 5+ | 11.2 | 1.1 |  | 2070.20 | 3+ | 13.8 | 0.9 |  | 1552.86 | 2+ | 12.4 | 0.6 |
| 15 V | 1863.31 | 5+ | 11.2 | 1.0 |  | 2070.20 | 3+ | 13.7 | 1.1 |  | 1552.38 | 2+ | 12.6 | 0.7 |
| 20 V | 1862.89 | 5+ | 11.2 | 1.0 |  | 2069.86 | 3+ | 13.8 | 1.0 |  | 1552.90 | 2+ | 12.6 | 0.7 |
| 25 V | n.o. | - | - | - |  | 2069.86 | 3+ | 13.8 | 1.1 |  | 1552.90 | 2+ | 12.6 | 0.7 |
| 30 V | n.o. | - | - | - |  | 2069.84 | 3+ | 13.8 | 0.9 |  | 1552.40 | 2+ | 12.6 | 0.6 |
| 35 V | n.o. | - | - | - |  | 2074.82 | 3+ | 14.0 | 1.1 |  | 1552.38 | 2+ | 12.6 | 0.7 |
| 40 V | n.o. | - | - | - |  | n.o. | - | - | - |  | 1551.88 | 2+ | 12.4 | 0.5 |

1. Second measurement series b) n.o. not observed

Table S31: Drift times of Foldon6 trimer. dimer. and monomer ion signals at different collision cell voltage differences ^a)^.

| ΔCV | trimer ^b)^ | | | |  | dimer ^b)^ | | | |  | monomer ^b)^ | | | |
| --- | --- | --- | --- | --- | --- | --- | --- | --- | --- | --- | --- | --- | --- | --- |
|  | m/z | z | dt [ms] | FWHM |  | m/z | z | dt [ms] | FWHM |  | m/z | z | dt [ms] | FWHM |
| 2 V | 1878.83 | 5+ | 11.2 | 1.5 |  | n.o. | - | - | - |  | n.o. | - | - | - |
| 5 V | 1878.64 | 5+ | 11.3 | 1.4 |  | 2087.17 | 3+ | 13.8 | 1.1 |  | 1566.86 | 2+ | 12.4 | 0.7 |
| 10 V | 1878.43 | 5+ | 11.3 | 1.0 |  | 2087.15 | 3+ | 14.0 | 1.0 |  | 1565.36 | 2+ | 12.6 | 0.8 |
| 15 V | 1878.45 | 5+ | 11.3 | 1.0 |  | 2086.81 | 3+ | 14.0 | 1.0 |  | 1565.36 | 2+ | 12.6 | 0.7 |
| 20 V | 1878.45 | 5+ | 11.3 | 1.1 |  | 2087.15 | 3+ | 14.1 | 1.0 |  | 1565.86 | 2+ | 12.6 | 0.7 |
| 25 V | n.o. | - | - | - |  | 2087.49 | 3+ | 14.0 | 1.0 |  | 1565.86 | 2+ | 12.6 | 0.7 |
| 30 V | n.o. | - | - | - |  | 2087.49 | 3+ | 14.1 | 1.1 |  | 1565.88 | 2+ | 12.6 | 0.8 |
| 35 V | n.o. | - | - | - |  | 2087.15 | 3+ | 14.0 | 1.0 |  | 1565.38 | 2+ | 12.6 | 0.7 |
| 40 V | n.o. | - | - | - |  | n.o. | - | - | - |  | 1565.38 | 2+ | 12.6 | 0.7 |

1. First measurement series b) n.o. not observed

Table S32: Drift times of Foldon6 trimer. dimer. and monomer ion signals at different collision voltage differences ^a)^.

| ΔCV | trimer ^b)^ | | | |  | dimer ^b)^ | | | |  | monomer ^b)^ | | | |
| --- | --- | --- | --- | --- | --- | --- | --- | --- | --- | --- | --- | --- | --- | --- |
|  | m/z | z | dt [ms] | FWHM |  | m/z | z | dt [ms] | FWHM |  | m/z | z | dt [ms] | FWHM |
| 2 V | 1879.06 | 5+ | 11.3 | 1.5 |  | n.o. | - | - | - |  | n.o. | - | - | - |
| 5 V | 1878.66 | 5+ | 11.3 | 1.3 |  | 2087.17 | 3+ | 14.2 | 1.0 |  | 1565.38 | 2+ | 12.7 | 0.7 |
| 10 V | 1878.66 | 5+ | 11.3 | 1.1 |  | 2087.17 | 3+ | 14.1 | 1.0 |  | 1565.37 | 2+ | 12.7 | 0.7 |
| 15 V | 1878.87 | 5+ | 11.5 | 1.1 |  | 2087.17 | 3+ | 14.0 | 1.1 |  | 1565.88 | 2+ | 12.7 | 0.7 |
| 20 V | 1878.26 | 5+ | 11.3 | 1.1 |  | 2087.51 | 3+ | 14.0 | 1.1 |  | 1565.88 | 2+ | 12.7 | 0.7 |
| 25 V | n.o. | - | - | - |  | 2087.51 | 3+ | 14.0 | 1.1 |  | 1565.88 | 2+ | 12.7 | 0.7 |
| 30 V | n.o. | - | - | - |  | 2087.19 | 3+ | 14.0 | 1.1 |  | 1565.88 | 2+ | 12.7 | 0.7 |
| 35 V | n.o. | - | - | - |  | 2087.51 | 3+ | 14.1 | 1.0 |  | 1565.38 | 2+ | 12.7 | 0.8 |
| 40 V | n.o. | - | - | - |  | n.o. | - | - | - |  | 1565.38 | 2+ | 12.7 | 0.7 |

1. Second measurement series b) n.o. not observed
